# Path Similarity Analysis: A Method for Quantifying Macromolecular Pathways

**DOI:** 10.1371/journal.pcbi.1004568

**Published:** 2015-10-21

**Authors:** Sean L. Seyler, Avishek Kumar, M. F. Thorpe, Oliver Beckstein

**Affiliations:** 1 Department of Physics and Center for Biological Physics, Arizona State University, Tempe, Arizona, United States of America; 2 Rudolf Peierls Centre for Theoretical Physics, University of Oxford, Oxford, United Kingdom; University of Illinois, UNITED STATES

## Abstract

Diverse classes of proteins function through large-scale conformational changes and various sophisticated computational algorithms have been proposed to enhance sampling of these macromolecular transition paths. Because such paths are curves in a high-dimensional space, it has been difficult to quantitatively compare multiple paths, a necessary prerequisite to, for instance, assess the quality of different algorithms. We introduce a method named *Path Similarity Analysis* (PSA) that enables us to quantify the similarity between two arbitrary paths and extract the atomic-scale determinants responsible for their differences. PSA utilizes the full information available in 3*N*-dimensional configuration space trajectories by employing the Hausdorff or Fréchet metrics (adopted from computational geometry) to quantify the degree of similarity between piecewise-linear curves. It thus completely avoids relying on projections into low dimensional spaces, as used in traditional approaches. To elucidate the principles of PSA, we quantified the effect of path roughness induced by thermal fluctuations using a toy model system. Using, as an example, the closed-to-open transitions of the enzyme adenylate kinase (AdK) in its substrate-free form, we compared a range of protein transition path-generating algorithms. Molecular dynamics-based dynamic importance sampling (DIMS) MD and targeted MD (TMD) and the purely geometric FRODA (Framework Rigidity Optimized Dynamics Algorithm) were tested along with seven other methods publicly available on servers, including several based on the popular elastic network model (ENM). PSA with clustering revealed that paths produced by a given method are more similar to each other than to those from another method and, for instance, that the ENM-based methods produced relatively similar paths. PSA was applied to ensembles of DIMS MD and FRODA trajectories of the conformational transition of diphtheria toxin, a particularly challenging example. For the AdK transition, the new concept of a Hausdorff-pair map enabled us to extract the molecular structural determinants responsible for differences in pathways, namely a set of conserved salt bridges whose charge-charge interactions are fully modelled in DIMS MD but not in FRODA. PSA has the potential to enhance our understanding of transition path sampling methods, validate them, and to provide a new approach to analyzing conformational transitions.

## Author Summary

Many proteins are nanomachines that perform mechanical or chemical work by changing their three-dimensional shape and cycle between multiple conformational states. Computer simulations of such conformational transitions provide mechanistic insights into protein function but such simulations have been challenging. In particular, it is not clear how to quantitatively compare current simulation methods or to assess their accuracy. To that end, we present a general and flexible computational framework for quantifying transition paths—by measuring mutual geometric similarity—that, compared with existing approaches, requires minimal a-priori assumptions and can take advantage of full atomic detail alongside heuristic information derived from intuition. Using our *Path Similarity Analysis* (PSA) framework in parallel with several existing quantitative approaches, we examine transitions generated for a toy model of a transition and two biological systems, the enzyme adenylate kinase and diphtheria toxin. Our results show that PSA enables the quantitative comparison of different path sampling methods and aids the identification of potentially important atomistic motions by exploiting geometric information in transition paths. The method has the potential to enhance our understanding of transition path sampling methods, validate them, and to provide a new approach to analyzing macromolecular conformational transitions.

This is a *PLOS Computational Biology* Methods paper.

## Introduction

Protein function is intimately linked with the mechanistic nature of conformational transitions—a central problem in computational biophysics is to determine the function of a protein given its 3D structure [1–3]. Proteins such as enzymes, molecular motors and membrane transporters behave much like nano-molecular machines that perform mechanical or chemical work by undergoing conformational transitions between two or more metastable states. Large scale conformational changes comprise the slowest frequency motions of a macromolecule and can take place on the millisecond time scale and beyond. Equilibrium molecular dynamics (MD) is arguably one of the most robust approaches to simulating macromolecular dynamics, in large part due to the availability of full atomistic detail [4]. It has has been the workhorse tool for studying the protein structure–function connection [5]. However, conformational transitions are rare events: crossing events in the transition region of phase space take place much faster than the (waiting) time scales of metastable equilibria, often by several orders of magnitude. Equilibrium simulations thus disproportionately sample metastable states instead of transition events—the so-called sampling problem—greatly limiting their ability to generate conformational transition paths [6].

Enhanced path-sampling methods and other computational approaches have been developed to mitigate the sampling problem inherent to macromolecular transition events, permitting the observation of physics on time- and length-scales inaccessible to equilibrium MD (see [7–12] for reviews). Conformational sampling in trajectory-based (i.e., dynamics-based) methods [13–21] is accelerated by reducing the computational cost per time step and/or by minimizing the total number of time steps needed for sampling [10]. Non-dynamical approaches can be roughly divided into the class of minimum (free) energy path (MEP/MFEP) methods [22–30], including elastic network model (ENM) approaches [31–35], and prior-information/geometry-based algorithms [36–39]. A large number of the aforementioned methods overlap algorithmically or are similar in spirit; many are also directly amenable (or can be adapted) to performing free energy calculations. Presently, however, the full extent to which such coarse-grained (CG) or biased MD approaches can replicate physical transition ensembles is unknown, especially given the diversity of physical assumptions of the various models. Thus, tools aiding more rigorous inspection of the capabilities and effectiveness of path-sampling methods are needed. In a more general sense we need a means to compare the protein motions, i.e. the transition paths, in an unbiased manner that makes use of all the available structural information.

### Approaches to transition path analysis

Conformational transition paths are represented by sequences of (snapshots of) conformers in 3*N*-dimensional configuration space, making it difficult to examine—both visually and quantitatively—their character without resorting to dimensionality reduction in a collective variable (CV) space. Native contacts analysis (NCA), for example, is a general approach frequently used to characterize protein folding pathways [40] and enables dimensionality reduction via a projection onto 2D native contacts (NC) space. NCA has the property that structural contacts are defined without reference to another structure, making NC space projections particularly useful when good reaction coordinates are not known a priori. Another common approach is principal component analysis (PCA), a tool that can be used to visualize conformational dynamics in a lower-dimensional subspace spanned by several principle components (PCs) [41, 42]. An important aspect of PCA is that motion along PCs can be viewed in real space, helping make complicated dynamical motions visually tractable.

Using NCA, PCA or other CV approaches cannot, however, guarantee that important dynamical motions will be captured in the projections—whether (and what) dynamical information is lost depends on the projection itself. It is clear that a quantitative method that can examine a full 3*N*-dimensional trajectory would help mitigate biases inherent to selecting a coordinate projection. We propose a general computational method named *Path Similarity Analysis* (PSA) to quantitatively compare 3*N*-dimensional macromolecular transition paths, which is based on the idea of measuring the geometric similarity between pairs of paths using path similarity metrics. Once distances are assigned to all pairs of paths, trajectories are then clustered by similarity. The structural determinants responsible for the difference between any two trajectories are extracted at the atomic level by exploiting properties of the underlying metric. Here we introduce the PSA approach, examine its suitability, performance, and limitations as a computational approach to quantifying path similarity and apply it to a toy system and conformational transitions of two proteins.

### Path metrics for measuring transition path similarity

Path similarity analysis (PSA) exploits the properties of a (path) metric function, *δ*, that measures a distance between a pair of piecewise-linear or polygonal curves, i.e., an ordered set of vertices connected by edges. A metric *δ* applied to curves *A*, *B*, *C* has the properties

δ(A,B)≥0 (1a)

δ(A,B)=0⇔A=B (1b)

δ(A,B)=δ(B,A) (1c)

δ(A,C)≤δ(A,B)+δ(B,C). (1d)

In particular, Eq 1b, the identity property, is essential since it implies that, given two curves *A* and *B*, if *B* were to be continuously deformed so as to monotonically decrease the distance *δ*(*A*, *B*), then *δ*(*A*, *B*) → 0 as *B* → *A*. That is, two curves must become identical as their mutual distance approaches zero so that decreasing values of *δ* correspond to increasing similarity. The other properties—non-negativity (Eq 1a), commutativity (Eq 1c) and triangle inequality (Eq 1d)—guarantee that *δ* behaves in the same way as any other metric usually used in structural comparisons (such as root mean squared distance) even though it compares whole paths and not just individual conformations.

PSA does not require the use of true metrics and can be used with any path distance function or other dissimilarity measure where only Eqs 1a–1c are satisfied. The triangle inequality (Eq 1d), which is a generalization of the transitive property, says that when two objects, *A* and *B*, in some metric space, are each close to a third object, *C*, in the same space, then *A* is close to *B* in the sense that the triangle inequality, *d*(*A*, *B*) ≤ *d*(*A*, *C*) + *d*(*B*, *C*), provides an upper bound on their distance apart. The triangle inequality is therefore important when comparing more than two objects, which is the common scenario when analyzing many conformational transitions. Although in the following we only consider true metrics, we also explore several distance functions that violate the triangle inequality in S1 Text. In the main part of this study, we consider two candidates for *δ*—the Hausdorff metric [43–45] and the discrete Fréchet metric [46, 47]—and illuminate situations where one might be selected in favor of the other. Given two paths as input, both metrics locate two points, one per path, corresponding to some notion of a maximal deviation between the paths. An important property of these metrics is that they are sensitive only to path geometry; they are insensitive to dynamical motions and associated physical time scales along paths. We provide a brief overview of these two path metrics in the context of conformational transitions.

#### Hausdorff metric

We start with a 3*N*-dimensional configuration space containing two paths *P* and *Q* represented, respectively, as sequences of conformations $\left\{{\left(p_{k}\right)}_{k=1}^{n}|p_{k}\in R^{3N},k=1,\ldots ,n\right\}$ and $\left\{{\left(q_{k}\right)}_{k=1}^{m}|q_{k}\in R^{3N},k=1,\ldots ,m\right\}$. The *Hausdorff distance* is defined as

$$\begin{array}{c} \delta_{H}\left(P,Q\right)=\max\{\delta_{h}\left(P|Q\right),\delta_{h}\left(Q|P\right)\}, \end{array}$$ (2)

where

$$\begin{array}{c} \delta_{h}\left(P|Q\right)=\max_{p\in P}\min_{q\in Q}d\left(p,q\right) \end{array}$$ (3)

is the *directed Hausdorff distance* from *P* to *Q*, and *d* is a distance metric on ℝ^3*N*^ (measuring point distances) [43]; the vertical bar (*P*∣*Q*) emphasizes that *δ*_*h*_(*P*∣*Q*) is not commutative. The function *δ*_*h*_(*P*∣*Q*) selects the point p* ∈ *P*, among all points in *P*, with the most distant nearest neighbor q* ∈ *Q* (as measured by *d*(p*,q*)). In the language of conformational transitions, we interpret *d*(*p*, *q*) as a putative structural similarity measure between conformers *p* and *q*, so that for some conformer *p*_*k*_ ∈ *P*, its structural “nearest neighbor” in *Q* is given by min_*q* ∈ *Q*_ *d*(*p*_*k*_, *q*). Thus, *δ*_*h*_(*P*∣*Q*) is the distance *d* associated with the conformer in *P* having the *most distant* or *least similar* nearest neighbor (in *Q*). The Hausdorff distance between *P* and *Q*, *δ*_*H*_(*P*, *Q*), is therefore the distance associated with the point—*of all points in P and Q*—with the least similar nearest neighbor, and implies that all points have a nearest neighbor that is at most *δ*_*H*_(*P*, *Q*) away.

#### Fréchet metric

Unlike the Hausdorff metric, Fréchet metrics are sensitive to the orientation (i.e., directionality) of paths; real transition paths are inherently directional which in principle makes Fréchet metrics superior to the Hausdorff metric. Informally, the *continuous Fréchet distance* can be visualized by considering a man walking on a path *P* and his dog on another path *Q* [47]. Both start at the initial points of their respective paths, and they are imagined to be connected by an elastic leash that remains taught so as to measure the distance separating them at all times. We then allow the man and dog to move independently on their respective paths under the condition that each progresses in a monotonic fashion (i.e., no backward steps) from start to finish. The Fréchet distance between *P* and *Q* is then defined as the length of the shortest leash necessary for the man and dog to move along their respective paths from beginning to end according to the aforementioned constraints. Formally, for two continuous curves *P* : [*a*_0_, *a*_1_] → ℝ^3*N*^, *a*_0_ < *a*_1_ and *Q* : [*b*_0_, *b*_1_] → ℝ^3*N*^, *b*_0_ < *b*_1_ that are parameterized with a real parameter, the continuous Fréchet distance corresponds to finding two specific continuous and monotonous parameterizations *α*:[0, 1] → [*a*_0_, *a*_1_] and *β*:[0, 1] → [*b*_0_, *b*_1_] (the “schedules” of the man and the dog along their paths) so that the largest point distance *d* for a given set of parameterizations is minimized [47],

$$\begin{array}{c} \delta_{F}\left(P,Q\right)=\min_{\alpha ,\beta }\max_{t\in [0,1]}d(P(\alpha (t)),Q(\beta (t))). \end{array}$$ (4)

Algorithms exist to solve this difficult problem in O(*nm* log *nm*) time for polygonal curves (where *n* and *m* are the number of vertices in each curve) [47] and various faster approximate solutions have been suggested [48, 49].

In this paper, however, we exclusively use the *discrete Fréchet distance*, *δ*_*dF*_, with the algorithm outlined by [50] as it is simpler and faster to compute (in O(*nm*) time) than its continuous counterpart, *δ*_*F*_. The formal definition of *δ*_*dF*_ considers two polygonal curves *P* and *Q* that are defined respectively by *n* and *m* ordered points in a metric space (*V*, *d*) for some metric *d*. Let the corresponding sequence of endpoints of the line segments of *P* and *Q* be respectively defined as *σ*(*P*) = (*p*_1_, …, *p*_*n*_) and *σ*(*Q*) = (*q*_1_, …, *q*_*m*_). In the product space *σ*(*Q*, *P*) ≡ *σ*(*P*) × *σ*(*Q*), we define a *coupling* between two polygonal curves *P* and *Q* as a sequence,

$$\begin{array}{c} C\left(P,Q\right)\equiv \left(p_{a_{1}},q_{b_{1}}\right),\left(p_{a_{2}},q_{b_{2}}\right),\cdots ,\left(p_{a_{L}},q_{b_{L}}\right), \end{array}$$ (5)

of *L* unique pairs of points (i.e., number of links) satisfying the following conditions: (1) The first/last pairs correspond to the first/last points of the respective paths (*a*_1_ = *b*_1_ = 1, *a*_*L*_ = *n* and *b*_*L*_ = *m*); (2) at least one point on a path (for a pair of points, one per path) must be advanced to its successive point, i.e., (*a*_*i*+1_ = *a*_*i*_ and *b*_*i*+1_ = *b*_*i*_ + 1) or (*a*_*i*+1_ = *a*_*i*_ + 1 and *b*_*i*+1_ = *b*_*i*_) or (*a*_*i*+1_ = *a*_*i*_ + 1 and *b*_*i*+1_ = *b*_*i*_ + 1) for all *i* = 1, …, *L*. The largest distance between a pair of points (*p*_*a*_*i*__, *q*_*b*_*i*__) for a given coupling *C* defines the coupling distance

$$\begin{array}{c} ∥C∥\equiv \max_{i=1,\ldots ,L}d\left(p_{a_{i}},q_{b_{i}}\right). \end{array}$$ (6)

Given the space of all possible couplings between *P* and *Q*, Γ_*P*, *Q*_, the *discrete Fréchet distance* between *P* and *Q* is the minimum coupling distance among all couplings in Γ_*P*, *Q*_:

$$\begin{array}{c} \delta_{dF}\left(P,Q\right)=\min_{C\in \Gamma_{P,Q}}∥C∥. \end{array}$$ (7)

**Fig 1:**
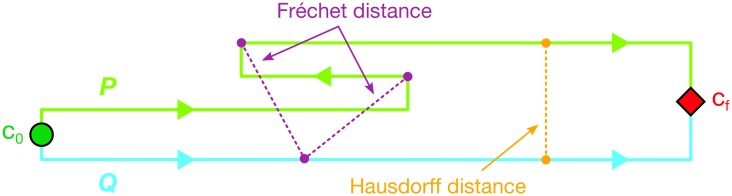
Comparison of Hausdorff and Fréchet distance. Two paths *P* (green) and *Q* (cyan) begin at state *c*_0_ and end at state *c*_*f*_ with directionality indicated by the arrows. The Fréchet distance *δ*_*F*_ and Hausdorff distance *δ*_*H*_ are given by the lengths of the purple and orange lines, respectively. The purple lines are the same length and correspond to the minimally stretched Fréchet “leash”; the orange line spans a pair of points separated by the Hausdorff distance (only one is shown because in this case there are infinitely many pairs of points with the same *δ*_*H*_). Due to the backtracking of path *P* toward state *A*, combined with the monotonicity (no-backward-movement) constraint of the Fréchet metric, *δ*_*F*_ > *δ*_*H*_.

The continuous Fréchet distance constitutes a lower bound on the discrete Fréchet distance, *δ*_*F*_ ≤ *δ*_*dF*_, because *δ*_*F*_ accounts for points along the (straight) edges connecting the vertices, whereas *δ*_*dF*_ only takes the vertices themselves into consideration [50]. Furthermore, if we define the maximum edge length for a polygonal curve *P* to be the largest distance between consecutive points in *P*, *d*_max_(*P*) ≡ max_*i* = 1, …, *p*−1_ *d*(*p*_*i*_, *p*_*i*+1_), we can set an upper bound on *δ*_*dF*_ given two polygonal curves *P* and *Q* so that *δ*_*F*_(P, Q) ≤ *δ*_*dF*_(*P*, *Q*) ≤ *δ*_*F*_(*P*, *Q*) + max{*d*_max_(*P*), *d*_max_(*Q*)} [50]. Thus, *δ*_*dF*_ differs from *δ*_*F*_ by no more than the longest edge among both paths and, to good approximation, *δ*_*dF*_ ≈ *δ*_*F*_ for typical trajectories with regularly spaced conformations. Hereafter we refer to the discrete Fréchet distance as simply the Fréchet metric (distance) with symbol *δ*_*F*_ for brevity. The Fréchet distance is bounded from below by the Hausdorff distance for any given pair of piecewise-linear curves [51] (*δ*_*F*_ ≥ *δ*_*H*_) because for convex polygonal curves the Fréchet and Hausdorff distances are equal [52] while for other path geometries the Fréchet distance can become arbitrarily larger than the Hausdorff distance [48]. In the case of macromolecular trajectories, the case of backtracking appears particularly relevant because of its conceptual link to a random walk and its connection to thermal fluctuations. If one path runs backward along some portion relative to another path, the Fréchet distance will increase with the extent of the backtracking, whereas the Hausdorff distance will be unaffected since it ignores the direction of path traversal (Fig 1).

#### Measuring structural similarity

Both the Hausdorff and Fréchet distances defined in Eq 2 and Eq 7, respectively, are defined in terms of a point metric *d*(*p*, *q*) on 3*N*-dimensional configuration space that measures the distance (i.e., similarity) between conformations *p* and *q*. We employ the root mean square distance (rmsd) defined in the usual way as

$$\begin{array}{c} d_{\text{RMS}}\left(p,q\right)=\sqrt{\frac{1}{N}\sum_{i=1}^{3N}{\left(p_{i}-q_{i}\right)}^{2}}, \end{array}$$ (8)

where *N* is the number of atoms, and ${\left\{p_{i}\right\}}_{i=1}^{3N}$ and ${\left\{q_{i}\right\}}_{i=1}^{3N}$ define the configuration space coordinates of conformations *p* and *q*, respectively.

It should be noted that Hausdorff and Fréchet metrics can be defined in terms of other point metrics to measure and thus emphasize different aspects of macromolecular structure or topology. For example, one could choose to measure the similarity of two protein conformers by quantifying the percentage of shared contacts. Another promising approach may be to integrate information-based metrics used for measuring the similarity of protein ensembles [53]. In this paper, we exclusively used the best-fit rmsd as the point metric due to its simplicity and widespread use, helping to connect with familiar intuitions and avoid obfuscating the examination of the path metrics themselves.

#### Previous studies and alternative approaches

The Hausdorff metric has found applications in image comparison [43], while the orientation-dependent Fréchet metric has been used for handwriting recognition and searching handwritten documents [54], and comparing trajectories of moving objects in geographic information systems [55]. Both metrics have also found applications in biology for protein structure alignment [56, 57] and protein homology analysis [58, 59].

To our knowledge, the Hausdorff and Fréchet metrics have not been widely used as general tools to quantify macromolecular pathways. However, recently two studies employed Fréchet distances to assess convergence of transition paths to an optimal path. Jiang et al. [60] used the same discrete Fréchet metric as used in this study to assess the convergence of a swarms-of-trajectories string method. Dickson et al. [61] employed a variation of the discrete Fréchet distance where the coupling distance was defined as the average distance between all pairs in a coupling (instead of the maximum distance as in Eq 6); this *discrete average Fréchet* distance was used in combination with an adaptive biasing force method to assess the convergence to an optimal path in an a priori CV space and was found to produce easier-to-read results by reducing statistical noise compared to the conventional metric. We explore this distance function in more detail in S1 Text along with a type of average Hausdorff distance. Protein folding pathways have been compared quantitatively but not with Hausdorff or Fréchet metrics. Several such studies utilized native contacts-based path (dis)similarity measures [62–65]. In particular, both Graham et al. [64] and Lindorff-Larsen et al. [65] used dissimilarity scores to assign individual paths to folding pathways using clustering. Different methods to sample conformational transitions were compared by Huang et al. [66], who contrasted the original targeted MD (TMD) algorithm [13] with a harmonic restraint variation of TMD (also known as “steered MD” (SMD) or “restrained TMD” (rTMD) [67])—and biased MD (BMD) approaches, and Ovchinnikov and Karplus [68], who analyzed the free energy profiles along the transition tubes surrounding the paths produced by several TMD variants.

The use of the Fréchet and Hausdorff path metrics on transition paths itself is not new; however, their application as general-purpose tools for quantitatively analyzing and comparing ensembles of transition paths—and extracting the molecular-scale determinants that dictate their differences—is, to our knowledge, novel. A particularly important advantage of the Hausdorff and Fréchet metrics is that they do not require a choice of progress variable, unlike metrics based on binning trajectory snapshots to compute path rmsds. While we emphasize that PSA suggests a general approach to quantitative transition path analysis using different structural and path metrics, we restricted our study to the Hausdorff and Fréchet path metrics implemented with the rmsd (as a structural similarity metric) to demonstrate the viability of a basic approach. We attempted to keep the underlying principles of PSA in view to engender future PSA-based analyses (such as quantifying putative reaction coordinates) and we stress that this study does not purport to exhaust all applications of PSA, nor represent an optimized application. Other path metrics—e.g., Fréchet with speed limits, direction-based Fréchet [69], or Fréchet with shortcuts for the analysis of noisy data [70]—may offer advantages in carrying out various analyses. The Hausdorff distance can be generalized as well to measure, for instance, distances between surfaces (instead of 1D curves) [71]. The multitudinous permutations that can be selected among the various path metrics, structural similarity metrics, clustering algorithms, etc. make PSA a flexible tool for trajectory analysis.

### Model systems

**Fig 2:**
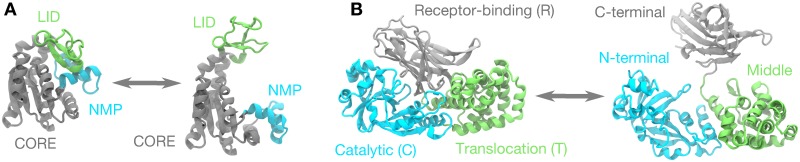
Macromolecular transitions studied with PSA. (A) The closed ↔ open transition for adenylate kinase (AdK) involves the hinge-like motion of the LID (green) and NMP (cyan) domains about the relatively stable CORE (gray). (B) Diphtheria toxin (DT) can exist in two different crystallographic conformations that are connected by a closed ↔ open transition involving the unravelling and swinging-open of the Receptor-binding (R or C-terminal) domain (gray), about the Catalytic (C or N-terminal; cyan) and the Translocation (T or middle; green) domains.

To investigate the applicability of the Hausdorff and Fréchet metrics to the problem of quantifying transition paths, we generated trajectories using an abstract toy system and we simulated conformational transitions of two globular proteins, the enzyme adenylate kinase (AdK) in its ligand-free form and diphtheria toxin (DT). The toy model was designed to gain an intuition for the path metrics and their applicability to highly fluctuating paths in high dimensions. AdK’s closed/open transition (Fig 2A) is a standard test case that captures general, essential features of conformational changes in proteins [12]. Alongside AdK in our analysis of transition ensembles, we also examined closed → open DT transitions (Fig 2B), which serves as a more challenging example due to the difficultly of capturing the putative unfolding and refolding required for conformational change [72].

AdK is divided into three domains: the ATP-binding (or “LID”) domain, residues 122–159 in the mesophilic *Escherichia coli* sequence (AK_eco_), and the AMP-binding (or “NMP” or “AMPbd”) domain, residues 30–59, move relative to the CORE domain [73–77] around conserved hinges [78] (Fig 2A). The conformational change can occur in the ligand-free (apo) state as demonstrated in multiple experimental studies [78–81] and corroborated by computational analyses (reviewed by Seyler and Beckstein [12]). Therefore, the apo AK_eco_ enzyme is a particularly suitable model system for studying general conformational transitions [12]. We produced transition paths between an open conformation of AdK [represented by chain A of PDB id 4AKE [77] from the Protein Data Bank [82] (PDB)], and a closed conformation (chain A of 1AKE [83] with ligand removed).

DT is believed to undergo a transition from an inactive closed conformation to an active open one, which includes a 180° rotation of a mobile domain [84] (Fig 2B). An open conformation was captured in a domain-swapped dimeric structure [85] and compared to the closed monomeric structure [86]. DT is divided into three domains, with the receptor-binding (R) domain, residues 380–535, being responsible for the majority of the opening and unrolling conformational motion about the translocation (T) domain, residues 179–379, and the catalytic (C) domain, residues 1–178. The conformational transition of a DT monomer was simulated previously and considered challenging for simulation methods [39, 72]. We simulated transition pathways of DT between a closed and open conformation based on chain A from the monomeric structure (PDB id: 1MDT [86]) and chain A from the domain-swapped dimeric structure (PDB id: 1DDT [85]), respectively.

## Methods

In the following we define the PSA approach as implemented in this study (using the metrics described in the Introduction), and we also summarize several alternative approaches to analyzing transitions that we employed alongside PSA for comparison. We describe how a range of conformational transition paths were generated to supply a variety of contexts in which to test PSA.

Molecular images were created with VMD [87] and the Bendix plugin [88]. Graphs were plotted with the Python libraries matplotlib [89] and seaborn [90], in particular its implementation of violin plots [91].

### Characterizing transition paths

#### Path similarity analysis (PSA)

The Hausdorff metric, *δ*_*H*_, and the discrete Fréchet metric, *δ*_*dF*_, defined in Eq 2 and Eq 7, respectively, were computed as described in the Introduction. Further details on the numerical implementation are provided in S2 Text. Both metrics are implemented as part of the MDAnalysis Python package [92] in the module MDAnalysis.analysis.psa, which is available as open source at www.mdanalysis.org under the GNU General Public License 2.

To analyze a set of *N* paths, we compute the *N*(*N* − 1)/2 unique pairwise Hausdorff and Fréchet distances. To present the data efficiently, we levied the versatility of hierarchical clustering [93] along with the visual power of a heat map-dendrogram representation to present a quantitative approach to visualizing the similarities of collections of paths. In agglomerative hierarchical clustering, similar objects are linked together in a pairwise fashion to form growing clusters in a bottom-up approach. The similarity between two objects is defined by a metric, while the similarity of clusters (i.e., sets of objects) is uniquely determined by a linkage criterion that computes inter-cluster similarity as a function of the pairwise similarities of the objects comprising each cluster.

Using the Hausdorff and Fréchet metrics as similarity measures, we employed Ward’s method [94] in conjunction with agglomerative hierarchical clustering as implemented in the SciPy Python package [95]. The Ward linkage criterion specifies a minimum variance criterion that minimizes the total intra-cluster variance. In light of the focus of this paper, we restrict our study to hierarchical clustering using primarily Ward linkage—details regarding this restriction are provided in S3 Text in the Supporting Information along with other relevant considerations in using cluster analysis to facilitate PSA.

#### Native contacts analysis (NCA)

For consistency with other methods used in this paper, we define a contact to be a residue pair whose C_*α*_ atoms are separated by a distance smaller than 8 Å. A *native contact* is a contact present in a reference structure. Given a transition path, the fraction of native contacts *Q* [96] is the fraction of contacts in a native structure that are present in a transition structure. We then define Q1 and Q2, for any intermediate conformer in a transition, as the fractions of native contacts with respect to an initial and final structure, respectively. Transition paths are projected onto 2D *Q*_1_-*Q*_2_ (NC) space by parametrically plotting the percentage of contacts relative to the initial and final states.

#### Comparison with a linearly interpolated path

**Fig 3:**
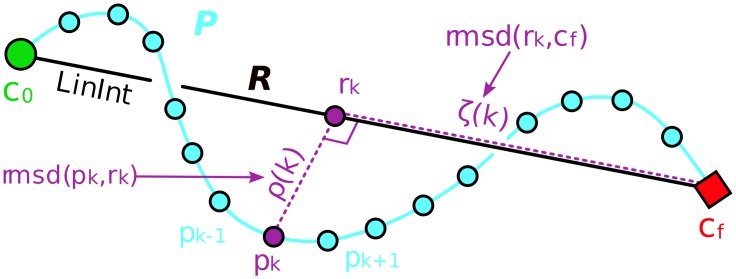
Comparison with a linearly interpolated path. A hypothetical transition pathway *P* (cyan line) in a 3D configuration space composed of a discrete number of conformer snapshots (cyan circles) connects an initial state (green circle), *c*_0_, and final state (red diamond), *c*_*f*_. The reference path *R* (black line) is represented by LinInt. Each snapshot *p*_*k*_ is associated with its projection, *r*_*k*_, on *R*; the progress, *ζ*(*k*), is the rmsd between *r*_*k*_ and *c*_*f*_ (dashed purple line along *R*) and the displacement, *ρ*(*k*), is the rmsd between *p*_*k*_ and *r*_*k*_ (dashed purple line perpendicular to *R*).

A simple way to quantify the geometry of a single transition path is to measure its orthogonal separation, *ρ*, from a reference path as a function of progress, *ζ*, along the reference path (Fig 3). In this way, any transition path can be projected in a 2D space depicting “displacement” (*ρ*) versus “progress” (*ζ*) relative to a reference path. We selected naive linear interpolation (LinInt) to serve as a simple zeroth-order reference. Note that, in comparison with PSA, this approach necessitates defining an explicit progress measure in the form of a reference path—which may not be appropriate beyond relatively simple examples like the AdK transition—and is furthermore not amenable to direct pairwise comparisons among a large ensemble of transition paths.

Given two boundary conformations {*c*_0_, *c_f_*} ∈ ℝ^3*N*^ in 3*N*-dimensional configuration space with reference path *R* embedded in ℝ^3*N*^ (that linearly interpolates *c*_0_ and *c*_*f*_), and a piecewise-linear (transition) path *P* embedded in ℝ^3*N*^ and composed of a sequence of conformations, ${\left(p_{k}\right)}_{k=1}^{m}$, where *m* is the number of time steps, we compute for each *p*_*k*_: (1) the rmsd between *p*_*k*_ and its orthogonal projection onto *R*, *r*_*k*_,

$$\begin{array}{c} \rho \left(k\right)=d_{\text{RMS}}\left(p_{k},r_{k}\right), \end{array}$$ (9)

and (2) the rmsd between *r*_*k*_ and final state *c*_*f*_,

$$\begin{array}{c} \zeta \left(k\right)=d_{\text{RMS}}\left(r_{k},c_{f}\right) \end{array}$$ (10)

(see Fig 3). A transition path can then be projected onto *ζ*-*ρ* space by parametrically plotting *ζ*(*k*) versus *ρ*(*k*) for all values of *k*. For a path beginning at *r*_0_ = *c*_0_, the rmsd to the final structure is given by the rmsd between the initial and final states, *ζ*(0) = *d*_RMS_(*c*_0_, *c*_*f*_), while the rmsd for a path ending at *r*_*m*_ = *c*_*f*_ is *ζ*(*m*) = *d*_RMS_(*c*_*f*_, *c*_*f*_) = 0.

Defining *ρ* using the rmsd permits a close connection with PSA in the following way: the maximal rmsd of a path *P* from LinInt, $\max_{k=1}^{m}\left\{\rho \left(k\right)\right\}$ will be the Hausdorff distance between *P* and LinInt, *δ*_*H*_(*P*,LinInt), when *P* is restricted to the region of configuration space between the boundary conformations (and assuming that structural alignment prior to rmsd measurement was performed identically). Furthermore, when *P* does not “backtrack”, *ζ*(*k*) is monotonically decreasing—indeed, *P* can be said to backtrack (with respect to some reference path) when *ζ*(*k*) is *not* monotone—and the Hausdorff and Fréchet distances coincide: $\max_{k=1}^{m}\left\{\rho \left(k\right)\right\}=\delta_{F}\left(P,\text{LinInt}\right)=\delta_{H}\left(P,\text{LinInt}\right)$.

#### Heuristic collective variables

While dimensionality reduction can be useful for visualizing and identifying functional protein motions, selecting the collective variables that span the projected space and adequately describe a conformational transition is nontrivial [97, 98]. Choosing heuristic coordinates for a given system often requires strong physical intuition, something that may be absent when studying new or complicated transitions. In general, the determination of reaction coordinates and/or order parameters can be guided by quantitative methods, such as principal component analysis or the construction of isocommittor surfaces. In the relatively simple case of AdK’s closed ↔ open transition, several viable order parameters have been used as low-dimensional descriptions [12].

To explicitly illustrate the uses and limitations of heuristic collective variables, and to make a connection with previous work, we examine the AdK closed ↔ open transition (Fig 2A) in 2D angle-angle space [99]. The NMP-CORE angle *θ*_NMP_ is formed by the geometric centers of residues 115–125 (CORE-LID), 90–100 (CORE), and 35–55 (NMP) of *E. coli* AdK. Likewise, *θ*_LID_ is defined as the angle between residues 179–185 (CORE), 115–125 (CORE-hinge-LID), and 125–153 (LID). As many of the methods we studied used C_*α*_-only models, we defined NMP-CORE and LID-CORE angles by exclusively using the C_*α*_ atoms of the residues. The angle-angle space defined by (*θ*_NMP_, *θ*_LID_) quantifies the degree to which NMP and LID are open and the sequence in which they open (close) for the closed → open (open → closed) transition.

### Generating transition paths

We first describe the toy model system used to supply simple transitions for testing purposes. We then summarize the path generation—using a variety of enhanced path-sampling methods—of closed → open transitions of AdK and DT, which serve as more realistic representations of conformational transitions.

#### Toy model: Double-barrel potential

To determine the extent to which the Hausdorff and Fréchet metrics are suitable for measuring transition paths, we constructed a toy system to generate well-defined trajectories driven by a one-way ramp potential and subject to thermal noise; the resulting paths in configuration space can be viewed as thermally-perturbed straight lines. For our purposes, transition progress was measured by the center-of-mass distance of a group of particles moving along the ramp so that a transition was completed once the center-of-mass trajectory crossed a threshold value.

**Fig 4:**
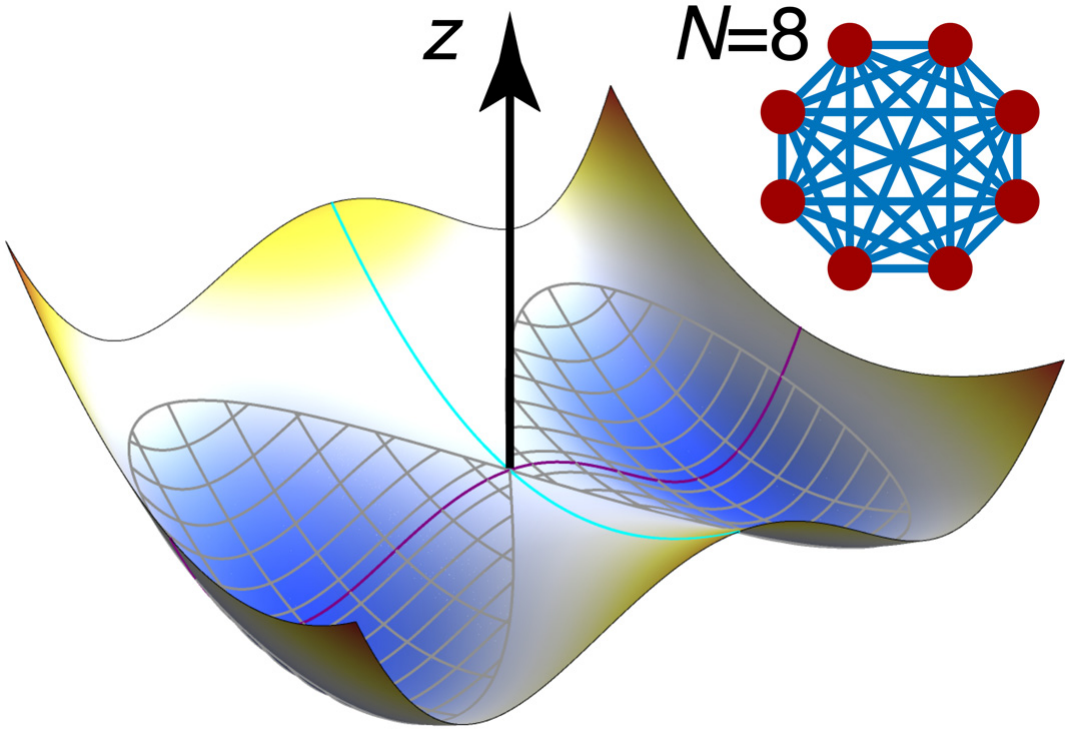
The toy model consists of a cluster of connected particles moving in a double-well potential along the *z*-axis under the influence of a linear ramp potential (not shown). In the cluster for *N* = 8, each particle (red) is connected to every other particle with a harmonic spring (blue) of equilibrium length 0 (cluster not shown to scale.) The potential landscape for constant *z* forms a “double barrel”—red (blue) regions correspond to high (low) energies—is parabolic along the *x*-direction (cyan line), and has a double-well shape in the *y*-direction (purple line), which produces a central barrier separating two “barrels” (gray crosshatching). A saddle point is located at the intersection of the cyan and purple lines. Motion in this landscape is biased toward either of the low-energy barrels, but transitions between barrels are possible at finite temperatures.

The toy system is defined as a group of *N* particles connected by harmonic springs subject to Brownian dynamics in a 3D potential energy landscape (Fig 4). Individual particles were connected in analogy to a complete graph, with vertices and edges respectively representing particles and springs. Spring equilibrium distances were set to zero separation for simplicity. Differing dimensionalities of the configuration space were examined by varying the number of particles *N*. The external potential was given a double-well shape in the *y*-direction and a parabolic shape in the *x*-direction (centered at *x* = *y* = 0), ensuring that particle clusters are confined to one of two “barrels” running along the *z*-direction (Fig 4). The energy barrier between the tubes was set to a height of 2 *k*_*B*_ *T* (∼ 5 kJ/mol) at *T* = 300 K. We set up a ramp potential sloping down toward increasing *z* (i.e., a constant potential energy gradient in the positive *z* direction) to induce large-scale transitions from small to large values of *z*.

To construct a properly coarse-grained system, we required zero-temperature cluster dynamics to be identical for all *N*-particle clusters (given sensibly chosen initial conditions). Spring constants, particle masses and sizes, and the external potentials were scaled so as to preserve the average diffusive behavior of a cluster. Furthermore, spring constants were chosen to be large enough to prevent clusters from splitting themselves across the central barrier (where some particles in the cluster fall to one tube and some fall to the other). Particles comprising a cluster were furthermore initialized at the same location so that zero temperature center-of-mass trajectories would be independent of particle number, *N*. It should be emphasized that this toy model was not intended to replicate a real physical system, but primarily served to build intuition prior to studying conformational pathways in realistic protein systems. More detailed information about the construction of the double-barrel system is provided in S4 Text in the Supporting Information.

#### Simulation methods and systems

The path-sampling methods comparison was performed using the AdK closed → open transition—between the (initial) closed conformation (PDB id 1AKE:A) and final (open) conformation (PDB id 4AKE:A)—as a testbed. We used eight methods available on publicly accessible servers [31, 72, 100–104], two in-house methods (DIMS, dynamic importance sampling [105], and FRODA, Framework Rigidity Optimized Dynamics Algorithm [39]), and targeted MD (rTMD [67]) using local simulation resources (see Tables 1 and 2 for overviews). DIMS and FRODA were additionally used to generate example ensembles of AdK and DT transitions (200 transitions per method per protein, 800 total) for ensemble-based and Hausdorff pairs analysis. In principle, other path-sampling methods could be included in a comparison and, in the future, it would be worth exploring alternative methods such as the finite-temperature string method [106], weighted ensemble dynamics [107, 108], milestoning [109], transition path sampling [110], non-equilibrium umbrella sampling [111] or forward flux sampling [112], to name a few. Key aspects of each method used in this study are summarized below to help connect our results with physical intuition about the models. Each path-sampling method is described in the context of the energetics they model (Table 1) and the schemes by which paths are propagated or generated (Table 2). Additional details about the methods and the corresponding simulation settings that were used can be found in S5 Text.

**Table 1:** Modeling of energetics in tested path-sampling methods.

Res^a^	Name	Force field/potential^b^	Solvent energetics^c^	Mixing function/other energetics^d^
all-atom	DIMS[105]	CHARMM22/CMAP	ACS/ACE2 IS	*T* = 300 K
	rTMD[67]	CHARMM22/CMAP	Generalized Born IS	*T* = 300 K
	MDdMD[100]	bonds/angles: inf. sq-well	Lazaridis-Karplus IS	NBF: simple vdW/electrostatic, *T* = 300 K
	FRODA[39]	stereochemical constraints	hydrophobic contacts*	overlap/angle/H-bond constraints
	Morph[72]	CHARMM/XPLOR^†^	–	energy minimization of intermediate snapshots
	LinInt	–	–	–
C_*α*_-only	GOdMD[101]	bonds: inf. sq-well	–	NBF: Go-like + ENM-MetaD
	ANMP[102]	double-well ANM	–	*E*_mix_ = min{*U*_*i*_, *U*_*f*_}
	iENM[103]	double-well ANM	–	*E*_mix_ = *F*(*U*_*i*_, *U*_*f*_) (arbitrary), collision penalty
	MAP[31]	two ANMs, OM dynamics	overdamped Langevin^‡^	minimum OM action → 2 ODEs+BCs → path
	MENM-SD/SP[104]	double-well ANM	–	*E*_mix_ = *β*^−1^ln{exp[−*β*(*U*_*i*_ + *ϵ*_*i*_)] + exp(−*β*(*U*_*f*_ + *ϵ*_*f*_)]}

All MD-based methods use atomic resolution; Morph and LinInt are the only other methods with greater than C_*α*_ resolution. Except for MAP, ENM-based models define double-well potentials using different mixing functions of each anisotropic network model (ANM) constructed about each native states. MAP uses 2 ODEs, found by minimizing the Onsager-Machlup action for each ANM about the native states, and satisfying continuity conditions for positions and velocities at their interface. MENM-SD/SP assumes weak mixing: *T*_*m*_ = *T* (*β* = 1/*kT*_*m*_, is an adjustable parameter); in the limit of vanishing mixing, *T*_*m*_ → 0^+^, *E*_mix_ = min{*U*_*i*_, *U*_*f*_}, which is the same double-well potential used by ANMP.

^a^Resolution of the model.

^b^inf. sq-well, infinite square well; ANM, anisotropic network model; OM, Onsager-Machlup.

^c^IS, implicit solvent; FRODA does not have a solvent model; MAP assumes overdamped Langevin dynamics in using the Onsager-Machlup action.

^d^NBF, non-bonded forces; vdW, van der Waals potential; ENM-MetaD, elastic network model-based metadynamics; *E*_mix_, mixing function for two-state potential; *U*_*i*_ (*U*_*f*_), potential energy function about the initial (final) native state; OM, Onsager-Machlup; ODEs+BCs, ordinary differential equations plus boundary conditions.

*FRODA does not use a solvent model.

^†^Morph uses CHARMM/XPLOR relaxation to minimize energy of intermediate snapshots.

^‡^MAP assumes overdamped Langevin dynamics in using the Onsager-Machlup action.

**Table 2:** Approach to generating paths in tested path-sampling methods.

Type	Name	Dynamics	Path propagation/biasing^a^	Rev^b^	TS/Stoch^c^	Progress variable
perturbation MD	DIMS[105]	Langevin NVT	SR	N	Y/Y	rmsd-to-target
	rTMD[67]	Langevin NVT	moving harmonic restraint	N	Y/Y	rmsd-to-target
	MDdMD[100]	discrete MD	SR + essential dynamics	N	Y/Y	ssd-to-target^†^
	GOdMD[101]	discrete CG-MD	SR + metadynamics	N	Y/Y	ssd-to-target^†^
geometric targeting	FRODA[39]	–	stepwise-enforced rmsd constraint*	N	Y/(Y/N)	rmsd-to-target
CG-ENM	ANMP[102]	–	SD from SP (cusp min.) to minima	Y	N/N	–
	iENM[103]	–	parametric SP/fixed-point eqn.	Y	N/N	–
	MAP[31]	–	OM minimum action path	Y	N/N	–
	MENM-SD[104]	–	SD from SP to minima	Y	N/N	–
	MENM-SP[104]	–	parametric SP/fixed-point eqn.	Y	N/N	–
adiabatic mapping	Morph[72]	–	linearly interpolated snapshots	Y	N/N	–
linear interpolation	LinInt	–	linearly interpolated snapshots	Y	N/N	–

DIMS, rTMD, MDdMD, and GOdMD are all non-deterministic MD-based methods. DIMS and rTMD employ a conventional force field and Langevin dynamics in the canonical ensemble; the discrete MD algorithms used by MDdMD and GOdMD assume ballistic particle motion until a collision occurs—along with the depth of the interatomic square wells, momentum and energy conservation are used to determine outgoing momenta without explicitly computing forces. FRODA uses a non-physical dynamical algorithm to path-search stereochemically correct regions of configuration space. CG-ENM methods generate transitions by constructing low-energy paths in the potential energy landscape. Morph and LinInt linearly interpolate the position of each atom between the initial and final states.

^a^SR, soft ratcheting; SD, steepest descent; SP, saddle point; OM, Onsager-Machlup.

^b^Is the method exactly reversible?

^c^Is the algorithm based on a (physical or non-physical) time step? Is it stochastic?

*At each step, rmsd reduced by fixed amount while simultaneously enforcing other constraints.

^†^ssd, sum of squared distances to target (includes weighting that varies between MDdMD and GOdMD.

Two of the tested methods are based on MD combined with perturbation techniques (perturbation MD) designed to drive transitions between initial and final states. Two MD methods—**DIMS** MD [15, 105] (implemented in CHARMM c36b2 [113]) and TMD (implemented in NAMD 2.10 [114])—used the all-atom CHARMM22/CMAP force field [115, 116] with Langevin dynamics and implicit solvents (ACE [117] in CHARMM, Generalized Born [118] in NAMD) in the NVT ensemble at 300 K. NAMD’s TMD implementation uses a time-dependent harmonic restraint that moves toward a target conformation with constant velocity [67], instead of the original TMD approach introduced by Schlitter [13] that employed a stepwise holonomic constraint; in accordance with Ovchinnikov and Karplus [68], we refer to the NAMD implementation as *restrained TMD* (**rTMD**) to distinguish it from the original algorithm. DIMS and rTMD transitions were driven using the heavy-atom rmsd to the target structure. The soft-ratcheting DIMS algorithm moves towards the target by taking trial MD steps. Steps toward the target (decreasing rmsd-to-target) are accepted whereas backward steps are rejected with a finite probability; velocities are re-sampled (according to Maxwell-Boltzmann) until the step is accepted. rTMD moves a harmonic restraint to linearly decrease the rmsd-to-target. We generated three rTMD paths using a fast pulling speed and three with slower pulling. rTMD differs from DIMS in that explicit forces are introduced into the system Hamiltonian whereas DIMS effectively introduces an entropic force.

Maxwell-Demon discrete Molecular Dynamics [100] (**MDdMD**) and **GOdMD** [101] are similar in spirit and are the only two methods based on a physical dynamical model among the server-based transition path generation methods. Both are based on discrete MD combined with soft ratcheting and a type of essential dynamics sampling. MDdMD utilizes an atomistic representation and an implicit solvent model; GOdMD uses a C_*α*_ representation and neglects solvent effects. Both methods model bonded forces with infinite square-wells although MDdMD incorporates further detail by using simple potentials to describe van der Waals and electrostatic forces; GOdMD uses a Go-like potential to describe non-bonded forces. Both also include an additional form of biasing to ensure transitions follow essential deformation movements of a protein: MDdMD accepts steps when the transition vector (from the current conformer to the target) overlaps sufficiently with an essential transition vector (defined using eigenvectors from NMA on a Go-like potential about the initial or final state); GOdMD uses an ENM-based metadynamics approach to bias the sampling of essential deformation modes and to ensure that trajectories escape the initial minima.

The geometrical targeting algorithm, **FRODA** [39], is an approach designed to produce stereochemically acceptable transition paths. FRODA moves a structure toward a target conformation by decreasing the rmsd-to-target while enforcing stereochemical constraints such as bond distances and angles, backbone dihedrals, and contact constraints. In particular, FRODA can avoid steric clashes in an all-atom structure, something that may not be achieved by coarse-grained elastic network models (CG-ENMs) or algorithms using simple linear interpolation.

We also generated transitions using five CG-ENM-based methods. These particular models first construct two harmonic potential energy functions, based on anisotropic network models (ANMs), about initial and final native (crystallographic) states, which has the general form

$$U\left(X\right)=\frac{1}{2}\sum_{d_{ij}^{\;0}<R_{c}}C_{ij}{\left(d_{ij}-d_{ij}^{\;0}\right)}^{2}+\Delta U,$$

where the sum is taken over all unique pairs of C_*α*_ atoms separated by less than a specified cutoff distance, *R*_*c*_, and Δ*U* is the energy difference between the two states. For atoms *i* and *j*, *C*_*ij*_ is the force constant, *d*_*ij*_ is the Euclidean distance between them, and $d_{ij}^{\;0}$ is the corresponding distance in the native (crystallographic) structure. Force constants can be determined by fitting to isotropic crystallographic B-factors for instance. A double-well (two-state) potential landscape is constructed by combining the separate potentials. Given a two-state potential, transition paths are generated by connecting the two (end-state) minima along low-energy pathways. The ENM-based methods are distinguished primarily by their two-state energetics (i.e., mixing potential) and method of defining and searching for low-energy transition paths. The cutoff distance, *R*_*c*_, can be adjusted to some degree for all the tested ENM-based approaches, but a couple also enable modification of the force (spring) constants, *C*_*ij*_, and the end state energy difference, Δ*U*.

ANMPathway [102] (**ANMP**) forms a double-well landscape where the energy at each point is taken to be the smaller of the energies specified by the two wells; the wells intersect to form a cusp hypersurface in configuration space. A path is found by locating the minimum along the cusp and performing steepest descent (SD) toward both well minima. The Mixed Elastic Network Model [104] (**MENM**) employs a double-well function with a tunable mixing temperature whose purpose is to modulate the cusp-like intersection to provide a smooth transition in energy between the two wells. The method locates saddle points (SPs), and can use a steepest descent (SD) mode (**MENM-SD**) to generate paths from the SPs to the minima, or it can provide a parametric equation describing an SP path through the fixed points of the landscape. The interpolated Elastic Network Model [103] (**iENM**) is similar to MENM-SP in that it analytically solves for a parametric SP path, although it only requires a general form of a double-well potential function (does not use an explicit mixing function). Unlike the other ENM-based methods, MinActionPath [31] (**MAP**) does not use a mixing function. Instead, a path is generated by minimizing the Onsager- Machlup (OM) action—which assumes overdamped Langevin dynamics—with the two separate ANMs for the native states to derive two one-dimensional differential equations describing the minimum action paths in the region of each ANM. A unique transition path between the initial and final states is found by satisfying continuity boundary conditions in the positions and velocities at the interface.

To provide a point of comparison to one of the most simple approaches, we used the Yale morph server [72, 119] (**Morph**), which combines linear interpolation and optional energy minimization of the intermediate snapshots (i.e., adiabatic mapping), and we also used explicit linear interpolation to generate a single path between the end states (**LinInt**).

The main thrust of the path-sampling methods comparison is to demonstrate PSA’s viability and not necessarily to directly evaluate the performance of the sampling algorithms. As such, adjustable parameters for all simulations were left at their default values unless explicitly stated. Transitions were produced using the highest allowable resolution, i.e., using all non-hydrogen atoms when possible or only C_*α*_ atoms otherwise. For each method, three unique paths were generated by either re-running those with stochastic algorithms or, for the deterministic ones, by adjusting a single parameter; in the case of rTMD, six total simulations were performed [three each for fast (∼ 1 Å/ps) and slow (∼ 0.01 Å/ps) pulling speed; see S5 Text for further details]. DIMS, FRODA and MDdMD simulations, which produce a unique trajectory every run, were run three times each without altering initial settings. Three GOdMD runs were performed by changing the relaxation window (20, 50 and 100). Distinct trajectories for the deterministic, ENM- based algorithms were obtained by varying spring cutoff distances: one transition at the default value and two by decreasing/increasing the cutoff. Morph trajectories were produced by toggling energy minimization and structural pre-alignment settings, and a single LinInt trajectory was included as a zeroth-order reference. All other simulation settings were left at default values where possible. Simulations and analyses performed in this study are summarized in Table 3. Furthermore, as half of the methods were limited to C_*α*_ structures as inputs—the coarsest representation among the methods—all analyses were restricted to C_*α*_ trajectory representations to provide a lowest common denominator. Trajectories were also aligned to a common reference structure generated by aligning and averaging the CORE C_*α*_ coordinates of the 1AKE:A and 4AKE:A structures (see S6 Text in the Supporting Information for a description of the structural alignment procedures).

**Table 3:** Summary of simulations, calculations, and analyses.

Assessment	System	Transition	Path generation	# path samples	Analysis methods^†^
(1) Intuition and viability	double-barrel	*z*: 0 → 4 nm	Brownian + ramp	4×(2 ICs)	PSA (*δ*_*F*_), *δ*_*F*_ -*δ*_*H*_ distr/corr*
(2) Methods comparison	AdK	closed → open	various methods	3×(11 methods)	PSA (*δ*_*F*_/*δ*_*H*_ *), NCA, *ζ*-*ρ*, AA
(3) Transition ensembles	AdK	closed → open	DIMS, FRODA	200×(2 methods)	PSA (*δ*_*F*_ *), *δ*_*F*_-*δ*_*H*_ distr/corr*
	DT	closed → open	DIMS, FRODA	200×(2 methods)	PSA (*δ*_*F*_), *δ*_*F*_-*δ*_*H*_ distr/corr*
(4) Atomic detail from PSA	AdK	closed → open	DIMS, FRODA	200×(2 methods)	PSA (*δ*_*H*_-pairs)

*Result in Supporting Information

^†^Analysis methods: PSA, path similarity analysis; *δ*_*F*_, Fréchet distance; *δ*_*H*_, Hausdorff distance; *δ*_*F*_-*δ*_*H*_ distr/corr, Fréchet -Hausdorff distribution/correlation analysis; NCA, native contacts analysis; *ζ*-*ρ*, progress vs. displacement along path of linear interpolation; AA, angle-angle space.

## Results and Discussion

We subdivided our study in four parts to show how PSA can be used to answer a range of questions about macromolecular transition paths and pathways (see Table 3): (1) The path metrics were able to distinguish and categorize simple trajectories in a toy system, taking thermal motion and varying number of particles into account. (2) PSA could be used to compare different path-sampling methods and, when combined with more traditional low-dimensional projections on collective variables, provide insights into similarities and differences between different methods. (3) PSA was able to analyze path ensembles, opening the door to analyzing dynamical simulations with statistical approaches. (4) PSA enabled us to extract the molecular structural determinants responsible for differences in paths, thus linking the general analysis of high-dimensional transition paths to the specific molecular detail.

### Path similarity analysis of toy model transitions

**Fig 5:**
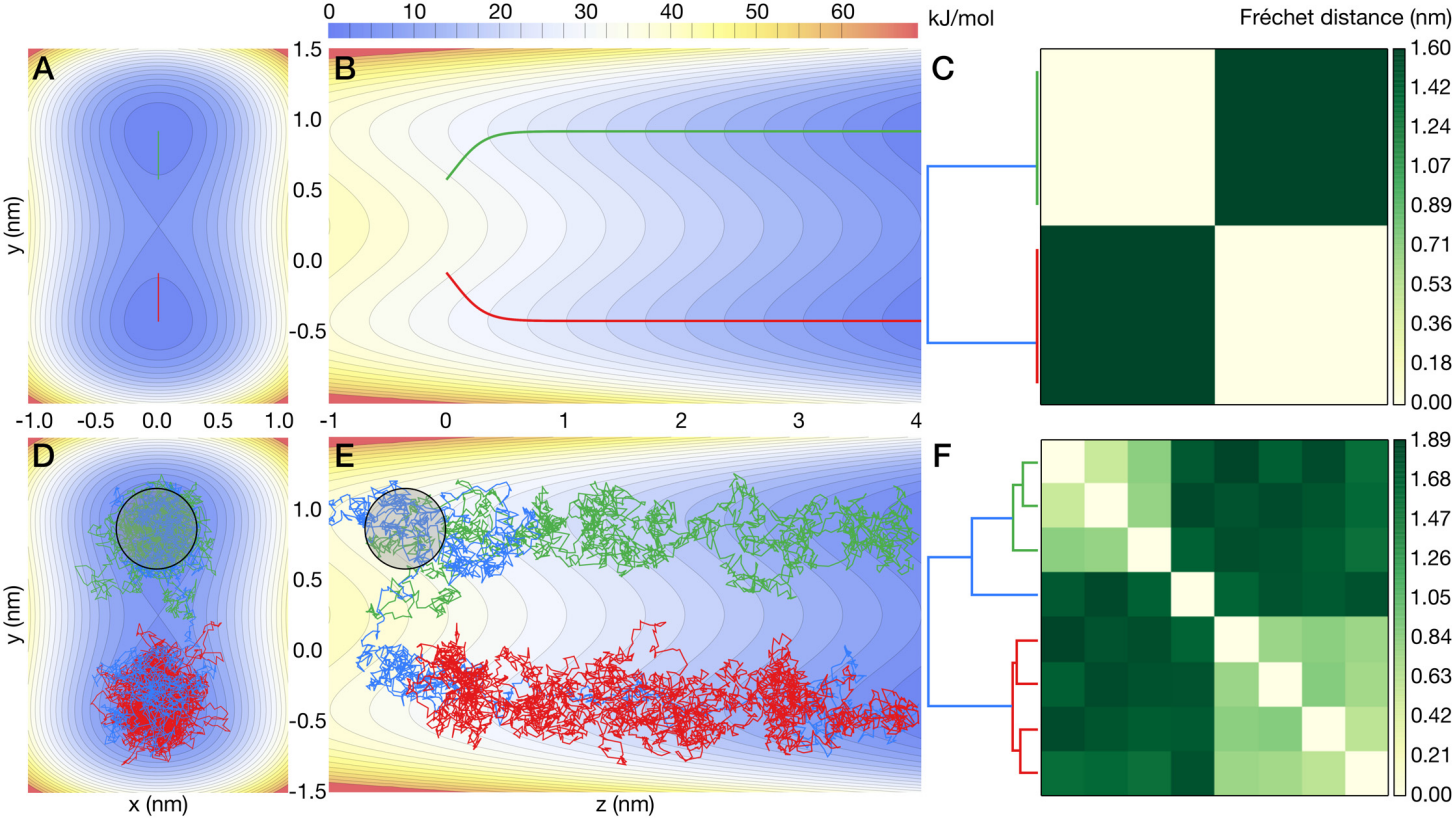
Double-barrel potential energy landscape projected onto the *xy*-plane and *yz*-plane. Groups of point masses (clusters) mutually connected by harmonic springs move under the influence of a transition-inducing ramp potential in the positive *z* direction and the two low-energy minima of the “barrels” at *y* = ±0.8. Colored lines depict the center of mass trajectories for each cluster. (A–C) trajectories at *T* = 0 K. (D–F) trajectories at *T* = 250 K. (A, D) Projection of paths onto the *xy*-plane together with the double-barrel potential. (B, E) Projection of paths onto the *yz*-plane. (C, E) Clustered heat maps summarize the Fréchet distances for all pairs of trajectories; dendrograms record cluster distances according to the Ward criterion. Trajectory colors in each row match the corresponding path(s) in the dendrogram. The trajectory-averaged radius of gyration for clusters at finite temperature is 0.35 (black circles).

We simulated one- and eight-particle cluster transitions in the double-barrel potential energy landscape between a starting state (defined as a center-of-mass location below *z* = 0*nm*) and a final state (*z* ≥ 4*nm*). Eight-particle simulations at zero and 250 K are shown in Fig. 5. The particles were weakly confined to one of two potential energy barrels separated by a 2 *k*_*B*_*T* barrier at 250 K (Fig 5A and 5D) and evolved under the influence of thermal diffusion and drift due to a linearly decreasing ramp potential in the *z* direction (Fig 5B and 5E). Simulations were run at temperatures between 0 K and 600 K in 50 K increments, with eight runs at each temperature. Trajectories were initialized such that two distinct groups of paths would be produced at zero temperature: for each temperature, we initialized half of the simulations to one side of the central barrier at (*x*_0_, *y*_0_) = (0 nm, 0.4 nm) and the other half at (0 nm, −0.4 nm).

At zero temperature, trajectories initiated at the same point progressed along identical paths due to the absence of thermal diffusion. Two trajectory groups were formed (Fig 5A and 5B), consistent with what was expected from the initial conditions. A clustered heat map of the Fréchet distances between the *T* = 0 K trajectories clearly showed two well-defined clusters (Fig 5C), containing four trajectories each, in both the structure of the dendrogram as well as the color division in the heat map. Due to thermal perturbations, higher-temperature trajectories exhibited substantial wandering (Fig 5D and 5E) and even produced a transition across the central barrier (blue trajectory in Fig 5E). In contrast with the zero temperature case, both the number of clusters and the clusters themselves were much more vaguely defined. Two clusters with four trajectories per cluster (red and green/blue trajectories, Fig 5D–5F) were still formed, although the blue trajectory, which underwent a barrier-crossing transition near *z* = −0.5 nm, is an outlier in the cluster with the three green trajectories.

Trajectory categorization for the toy model with PSA did not depend strongly on the dimensionality (cluster size) as thermal noise alone appeared to have a much more substantial influence (S1 Fig). In particular, we could not discern meaningful differences in the center of mass motions between one- and eight-particle clusters from the data. Furthermore, in the eight-particle case at 250 K, performing PSA using the full (24-dimensional) configuration space trajectories did not produce a different clustering than PSA applied only to the center of mass trajectories. The same analysis as above was carried out with the Hausdorff distance instead of the Fréchet distance to assess their relative discriminative powers. Both metrics produce similar results at temperatures below 300 K, each identifying two distinct pathways (S2 Fig). Between 350 K and 500 K, however, Hausdorff and Fréchet distance measurements started to become substantially uncorrelated (S3 Fig). This effect is likely due in part to the sensitivity of the Fréchet metric to backtracking (Fig 1), which may be amplified when the typical energy of thermal perturbations become comparable to the height of a potential barrier (2*k*_*B*_*T* at 300 K). High-temperature simulations (≥300 K) began to explore both tubes as if they were a single pathway (S2 Fig and S4 Fig).

Taken together, PSA was able to distinguish groups of paths in the presence of stochastic thermal motions as long as the thermal energy was lower than the energy scale of distinguishing features in the underlying energy landscape. The dimensionality of the problem did not appear to be an important factor. Fréchet and Hausdorff distances discriminated paths equally well with some small differences at high temperatures that likely reflect trajectory backtracking.

### Comparing enhanced path-sampling methods

In order to compare a selection of fast transition path sampling methods, three distinct trajectories were generated for the closed → open AdK transition as described in Methods.

#### Direct comparison using PSA

**Fig 6:**
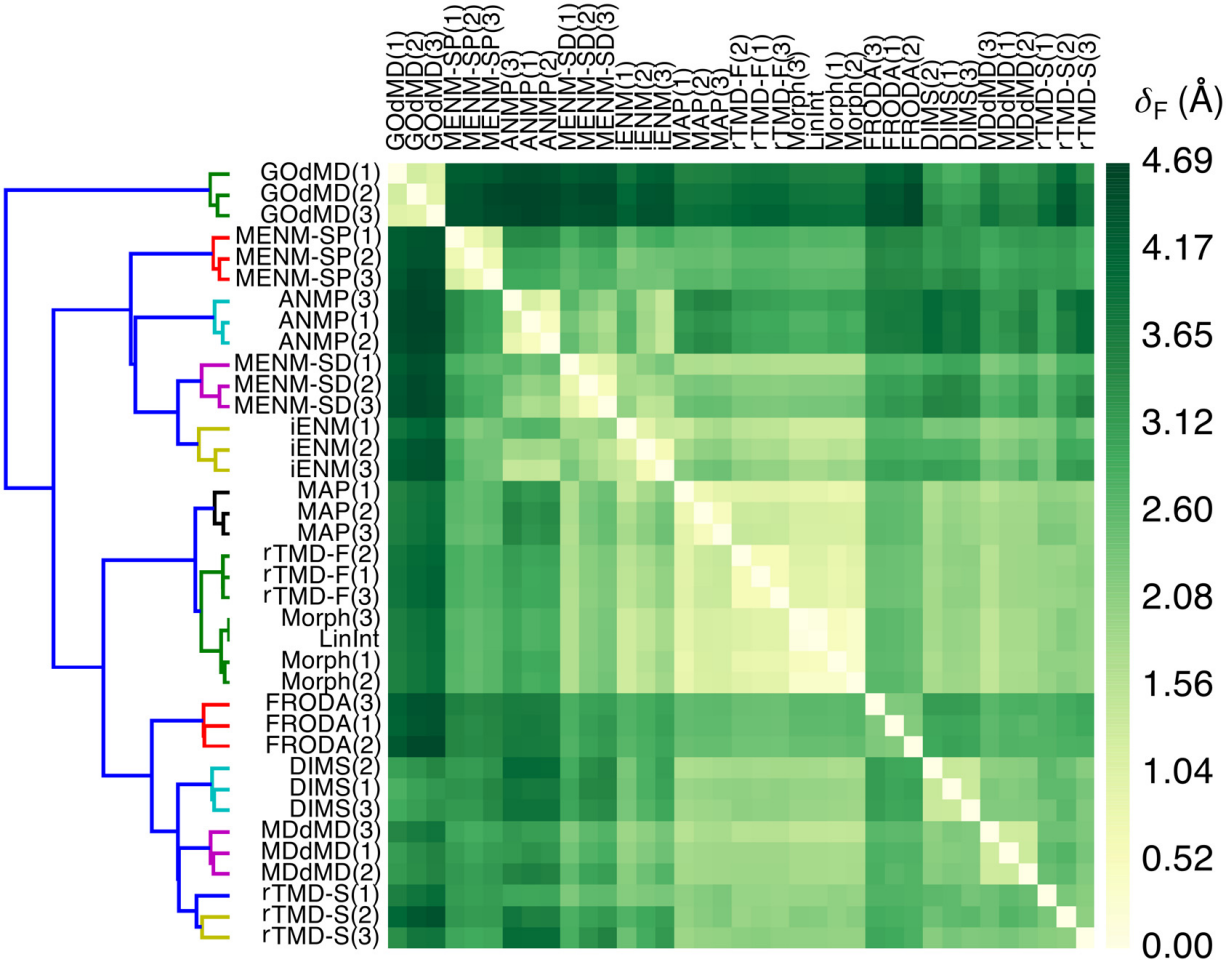
Path similarity analysis of trajectories generated by different path-sampling methods. The AdK closed → open transition was sampled three times (except LinInt) with different methods (see text). Smaller distances indicate transition paths with greater similarity. The dendrogram depicts a hierarchy of clusters where smaller node heights of parent clusters indicate greater similarity between child clusters. Fréchet distances *δ*_*F*_ are in Å and correspond to a structural rmsd in accordance with the rmsd point metric. See text for a description of the methods. S5 Fig contains the same data annotated with numerical values of *δ*_*F*_.

A total of 37 paths (eleven methods, three paths per method with the exception of six paths for rTMD, plus one LinInt path) were analyzed by computing the Fréchet distance between all possible pairs and clustering of the resulting (symmetric) distance matrix (S5 Fig). Using the same approach as with the toy model, the clustered distance matrix was translated to a heat map-dendrogram representation (Fig 6).

Paths from a given method were more similar to other paths from the same method than to those produced by a different method, as indicated by well-defined 3 × 3 squares along the heat map diagonal. Methods based on similar physical models tended to sample relatively similar pathways, while algorithmically distinct approaches appeared less likely to produce similar paths. For instance, Morph and LinInt both implement linear coordinate interpolation. Their paths are essentially identical (*δ*_*F*_ ≤ 0.5 Å), which indicates that additional features implemented in Morph, such as checking for steric overlaps, may not be relevant for the AdK transition. Another cluster was formed by the two MD-based importance sampling methods, DIMS and MDdMD, together with MD-based rTMD at slow pulling velocity (“rTMD-S”; Fréchet distance 2.1 Å ≤ *δ*_*F*_ ≤ 2.7 Å). In other cases, similarities and differences did not always follow an immediately obvious pattern. FRODA, which satisfies rigidity constraints during a transition but does not employ a potential energy function, nevertheless formed a cluster with DIMS, MDdMD, and rTMD-S (2.6 Å ≤ *δ*_*F*_ ≤ 3.1 Å). The grouping of FRODA with DIMS/MDdMD/rTMD-S appears, however, less strong than, for instance, the clustering of DIMS with MDdMD because for other choices of the linkage criterion FRODA is more distantly associated with the DIMS/MDdMD/rTMD-S cluster and a robust cluster of MAP/Morph/LinInt trajectories (see S6B–S6D Fig and further discussion in S3 Text). The fast-pulling rTMD (“rTMD-F”) and MAP trajectories were strikingly similar to the Morph paths (*δ*_*F*_ ≈ 1 Å), even though rTMD-F performs MD with an atomistic physics-based force field, whereas MAP’s energy function is based on an elastic network model and the path is generated via minimization of Onsager-Machlup action (and not just linear interpolation). Interestingly, the MAP/rTMD-F/Morph sub-cluster was grouped with the cluster formed by four of the dynamical algorithms (DIMS, MDdMD, rTMD-S, FRODA). The other four ENM algorithms—iENM, MENM-SD/SP, and ANMP—produced their own cluster, with MENM-SD and iENM being the most similar to each other. A careful examination of the heat map revealed that although MAP, rTMD-F, and Morph paths somewhat resembled iENM and MENM-SD paths (*δ*_*F*_ ≤ 2.5 Å), their overall patterns of Fréchet distances were very similar to DIMS/MDdMD/rTMD-S (as seen in the similar overall striping in the shading of the heat map) so that the “Morph-like cluster” rather clustered with these dynamical methods than with the “ENM cluster”. The GOdMD paths formed their own outlier cluster, appearing substantially different from all other methods (*δ*_*F*_ > 3 Å).

The classification of trajectories was found to be robust against use of different linkage functions in the clustering algorithm, provided that the linkage primarily assessed the *dissimilarity* of clusters (such as Ward’s criterion in Fig 6 and the complete/average/weighted linkage in S6B–S6D Fig) instead of similarity (single linkage in S6A Fig). Using the Hausdorff metric instead of the Fréchet metric did not change the clustering either and the Pearson correlation coefficient between *δ*_*H*_ and *δ*_*F*_ was very close to unity (S7 Fig). In S1 Text, alternative distance definitions, namely averaged Fréchet and Hausdorff distances (which are, however, not proper metrics), reduced the amount of detail in the clustering and resulted in an amalgamation of clusters into one large “dynamical methods cluster” (TMD-S, DIMS, MDdMD, GOdMD, FRODA), a “Morph-like cluster” (Morph, LinInt, TMD-S, MAP), and an “ENM cluster” (ANMP, iENM, MENM-SP/SD).

Without any input except the trajectories themselves, PSA produced distinct clusters that appeared to broadly distinguish between dynamical and non-dynamical path sampling methods. With the help of more specialized analyses to be described next we sought to further rationalize the observed pattern of clustering.

#### Native Contacts Analysis

**Fig 7:**
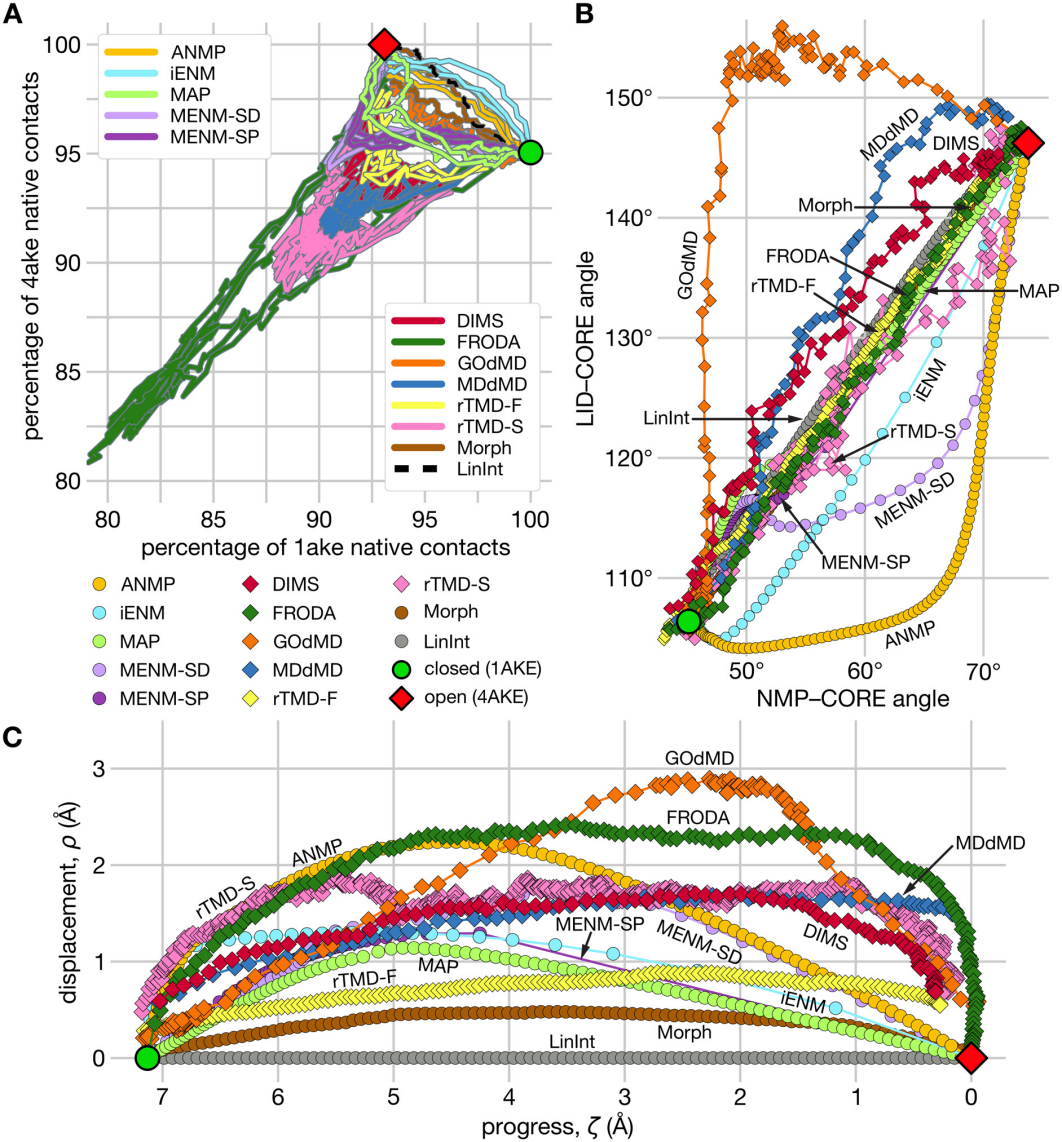
Projections of trajectory 2 of the AdK closed → open transition from each path-sampling method onto low-dimensional collective variables. The location of the initial structure is shown in each plot by the green circle, while the final structure is represented by the red diamond. (A) Projection of all pathways from the various path-sampling methods onto NC space. The horizontal axis corresponds to the percentage of contacts (of a transition snapshot) shared with the initial 1AKE:A structure (green circle) and the percentage of contacts in common with the final 4AKE:A structure (red diamond) is displayed on the vertical axis. The top-left legend identifies EN-based methods; the other methods are listed in the bottom legend. The LinInt path is shown for reference as a broken black curve. (B) Projection on NMP angle (*θ*_NMP_) *vs* LID angle (*θ*_LID_). In B and C, trajectories generated by the dynamical methods (DIMS, rTMD, FRODA, MDdMD, GOdMD) are plotted with diamonds and non-dynamical method trajectories with circles. (C) *ζ*-*ρ* space projection using LinInt as the reference path. Trajectory progress in *ζ*-*ρ* space is from left to right from higher to lower values of the progress variable *ζ*. MDdMD terminates at 1.5 Å C_*α*_ rmsd from 4AKE (red diamond); DIMS MD terminates at 0.5 Å heavy atom rmsd.

We performed two dimensional NCA on trajectories by measuring (for each conformer snapshot) the fraction of native contacts relative to the closed starting state (*Q*_1ake_) and to the open target conformation (*Q*_4ake_) as collective variables (Fig 7A). Using the NC trajectories, we examined the dynamic relationship of contact formation and breaking for each method. In general, the closed → open trajectories began on or near the right vertical axis, corresponding to the first conformers of the paths having (nearly) 100% of their contacts in common with the closed structure and around 95% of open state contacts. Most trajectories terminated at the top horizontal axis with the final conformers containing close to 100% of the final, open 4AKE:A structure contacts and about 93% of 1AKE:A contacts. The starting conformers of the DIMS NC paths only contained 96% of the contacts seen in the 1AKE crystal structure (*Q*_1ake_ = 0.96), which is to be expected given that the initial closed structure was energy-minimized and equilibrated prior to performing MD.

The five dynamical methods—DIMS, rTMD, FRODA, MDdMD, and GOdMD—produced somewhat noisy paths where the fluctuations took place along a positively sloping direction in NC space. A positive slope implies that contacts were simultaneously formed or broken relative to both native structures, which can be taken to be indicative of passage through a transition state that is distinct from either end state conformation. DIMS trajectories did not exactly reach the target structure (*Q*_4ake_ ≤ 0.98) as DIMS simulations were considered complete as soon as a conformer was within 0.5 Å heavy atom (non-hydrogen) rmsd from the target crystal structure 4AKE. MDdMD paths partly overlapped with DIMS paths during contact breaking but failed to reform them (*Q*_4ake_ < 0.94); as with DIMS, transition completion is determined by a cutoff—manually set to 1.5 Å C_*α*_ rmsd—due to the difficulties of convergence to a target using the soft-ratcheting biasing approach in MDdMD. DIMS and MDdMD broke a similar number of contacts relative to both states (around 8–9% and 9–10%, respectively). rTMD-S showed qualitatively similar behavior but broke up to about 12% of native contacts. The closely-knit cluster of DIMS, MDdMD and rTMD-S paths produced by PSA reflects the qualitative similarity of their NC trajectories. DIMS, MDdMD and FRODA all generated noisy, V-shaped NC pathways suggestive of a transition region, which supports the picture from PSA where these three methods form a loose cluster apart from the non-dynamical methods. FRODA clustered somewhat apart from the other three, correlating with the observation that FRODA trajectories in NC space exhibited the greatest contact breaking (*Q*_1ake_ = 0.82, *Q*_4ake_ = 0.80) of all methods tested. This behavior is not unexpected because FRODA achieves random motion by randomly displacing and rotating rigid units of the protein at the sub-amino acid level at each step prior to re-enforcing geometric constraints. As such, C_*α*_ fluctuations and, thus, native contact dynamics that would be prohibited by conventional potentials are permitted by the geometric model although constraints on the overall sequence and structure would nevertheless limit dramatic perturbations to the C_*α*_ rmsd. GOdMD paths, though quite noisy, followed a path more closely resembling those from the non-dynamical methods, particularly MAP and Morph.

Morph, LinInt, and two of the five ENM-based methods (ANMP and iENM) produced the shortest NC trajectories progressing directly to the target conformation with relatively little wandering, whereas the six MENM paths deviated noticeably toward the DIMS and FRODA trajectories in the latter half of the transition; MAP paths were also nearer the MENM pathways in location and shape than to the paths from the other ENM-based methods. The MENM paths and two MAP paths were unique among the non-dynamical methods in that they each contained a V-shaped, cusp-like feature where extra 4AKE:A contacts were broken (*Q*_4ake_ ≈ 0.91, 0.91 and 0.92, respectively) that were subsequently reformed toward the end of the transition. The rTMD-F NC paths were situated in an intermediate position between the other dynamical methods and the non-dynamical methods. Initially, only 1AKE:A contacts that do not exist in 4AKE:A were broken. Then the missing 4AKE:A native contacts were formed. The Morph, LinInt, ANMP and iENM paths, which were divided between two clusters in PSA, exhibited progress along negatively sloped NC space trajectories during which 4AKE:A contacts were formed while 1AKE:A contacts were simultaneously broken. However, the close structural correspondence between MAP, rTMD-F, and Morph paths in PSA was not recapitulated in NCA. On the other hand, the ANMP paths, which were reasonably similar to iENM in PSA (1.4 Å ≤ *δ*_*F*_ ≤ 2.7 Å in Fig 6) but fairly different from Morph (2.8 Å ≤ *δ*_*F*_ ≤ 3.1 Å), appeared fairly similar to both iENM and Morph in NC space.

Comparison of the NC projections of rTMD-S and rTMD-F indicated that the pulling velocity in rTMD directly affected the degree to which native contacts were broken and reformed. Consequently, different transition pathways were followed, as indicated by PSA, where rTMD-S clustered with the other dynamical methods and rTMD-F was most similar to LinInt and Morph.

NCA identified the same general division between the dynamical and non-dynamical methods as PSA, while some subdivisions within the dynamical/non-dynamical dichotomy are also borne out by both analyses, such as the closer grouping of MDdMD/rTMD-S/DIMS than FRODA/DIMS or FRODA/MDdMD. However, the cusp-like feature and overall qualitative similarity of the MENM and MAP trajectories in NC space that set them apart from the other non-dynamical methods is not obviously captured by PSA. The NC projection did not offer a clear hint as to why ANMP, iENM, and MENM-SD/SP were subdivided as they were in PSA and why GOdMD appeared as an outlier—two questions addressed by the following analysis of the transition paths projected onto *ζ*-*ρ* and angle-angle coordinates.

#### Projections into *ζ*-*ρ* and angle-angle space

PSA and NCA are both general transition path analysis methods that do not require knowledge of any system-specific order parameters or collective variables. We employ the *ζ*-*ρ* projection (distance from and progress along the path of linear interpolation) in order to resolve the remaining apparent discrepancies between PSA and NCA. Because good collective variables are known for the AdK transition [12], we also use a 2D projection onto domain angles [99] to connect the conclusions derived from the general analyses to visually intuitive structural motions of the closed → open transition.

In the *ζ*-*ρ* space projection (Fig 7C), the dynamical methods tended to obtain the greatest distance from the LinInt reference path near the end of the transition (*ζ* ⪅ 3.5 Å) whereas the non-dynamical methods peaked nearer the beginning. Thus, the dynamical/non-dynamical method dichotomy previously observed in both NCA and PSA was also present in *ζ*-*ρ* space. The structural interpretation of this behavior is, based on the projection into angle-angle space (Fig 7B), that the dynamical methods favored a pathway during which first the LID domain opens, followed by the NMP domain. Non-dynamical methods produced either NMP-opening-first paths or paths with brief LID-opening motions. In *ζ*-*ρ* space, however, dynamical methods produced paths with a greater average and peak (orthogonal) displacement from LinInt than non-dynamical methods (which cannot be discerned by apparent displacements in angle-angle space), further corroborating the clusterings from PSA.

Fast-pulling rTMD (rTMD-F), as a dynamical method, appeared as an exception to the dynamical/non-dynamical method dichotomy. However, both the projection onto domain angles and especially the *ζ*-*ρ* projection clearly showed that the rTMD-F path was very similar to LinInt (*ρ* < 1 Å in Fig 7C). rTMD with very high pulling velocities of the restraint potential moves the system almost exlusively in the direction of the restraint force. For an rmsd restraint, the gradient points exactly along the LinInt path. Therefore, rTMD-F functions more like LinInt or Morph and less like equilibrium MD with an additional bias potential and hence PSA clustered rTMD-F with LinInt and Morph (Fig 6).

MENM-SP was the most distant member in the cluster of the four ENM-based methods in PSA (Fig 6). Careful inspection of both angle-angle space (Fig 7B) and *ζ*-*ρ* (Fig 7C) revealed that the MENM-SP path contained a very large gap in the trajectory snapshots; the penultimate conformer was located in the first half of the transition (*ζ* > 4 Å), while the final snapshot was the open crystal structure end state. Such a big gap in the path affects the discrete Hausdorff/Fréchet distances because the distance between two MENM-SP paths with well-aligned gaps is unaffected whereas the distance between an MENM-SP path and one without gaps tends to be somewhat larger due to large point distances originating from the latter’s conformers in the portion of the transition where the gap occurs. ANMP was also somewhat of an outlier within the ENM cluster (Fig 6), which can be traced to its path being much farther away from the LinInt reference than any other ENM/Morph method (*ρ* ≈ 2.2 …2.3 Å versus *ρ* ≈ 1.3 Å; Fig 7C). Structurally, the NMP domain opened nearly all the way before much of the LID motion took place, in contrast with every other method (Fig 7B).

GOdMD produced the path with the greatest peak displacement (*ρ* ≈ 2.8 Å; Fig 7C), corresponding to complete LID opening before substantial NMP movement occured (Fig 7B). The results from GOdMD are unlike any of the other methods and therefore GOdMD is well-classified as an outlier by PSA (Fig 6).

PSA was able to group fast transition path sampling methods into distinct clusters. These groupings could be rationalized by employing projections on more specialized collective variables. An important observation was that transition paths were most similar to other transition paths generated by the same method. This conclusion was, however, based on a small sample of three paths per method. We then sought to extend our analysis to larger ensembles of paths that would provide a statistically more meaningful comparison.

### Comparing DIMS and FRODA transition ensembles

We applied PSA to transition path ensembles containing hundreds of trajectories to highlight several approaches to handling the statistical nature of dynamical path-sampling methods and illustrate the portability of our analyses to other systems. Ensembles of the AdK and DT closed → open transitions were analyzed. DT was selected in part to make contact with a previous study by Farrell et al. [39] as well as provide a more challenging example to demonstrate the ease with which PSA can filter erroneous trajectories from an ensemble. We focused on two methods, DIMS MD and FRODA, because they differ fundamentally in their energetic considerations yet still share several salient features: Heavy-atom representations were used for both methods for both AdK and DT. Both methods can generate path ensembles by employing a form of stochastic dynamics, and they both drive transitions (toward a target structure) with similar rmsd-to-target progress variables (DIMS uses the heavy-atom rmsd-to-target for the soft-ratcheting coordinate; FRODA attempts to gradually decrease the C_*α*_ rmsd to the target). Furthermore, our in-house implementations of DIMS MD methods allowed us to efficiently generate large numbers of transitions. Four unique ensembles and 800 total trajectories were generated: 200 pathways per method per protein. Details about trajectory alignment for both AdK and DT are provided in S6 Text of the Supporting Information.

For AdK, transition path trajectories generated with DIMS formed one cluster that was distinct from a second cluster containing all FRODA trajectories (see S8 Fig in the Supporting Information). The mean Fréchet distance 〈*δ*_*F*_〉 between DIMS and FRODA trajectories was 2.9 ± 0.1 Å, significantly higher than the mean within the FRODA (2.2 ± 0.1 Å) and DIMS ensemble (1.4 ± 0.2 Å). DIMS generated paths with smaller Fréchet distances among themselves than FRODA, while paths produced by a given method were notably more similar among themselves than when compared with paths from the other method, with no difference between Fréchet and Hausdorff distance (S10A Fig). These observations imply that while FRODA produced paths that sampled a larger region of AdK’s configuration space than DIMS, each method generated a unique pathway that can be viewed as a tube in configuration space whose diameter was smaller than the typical distance between the tubes.

**Fig 8:**
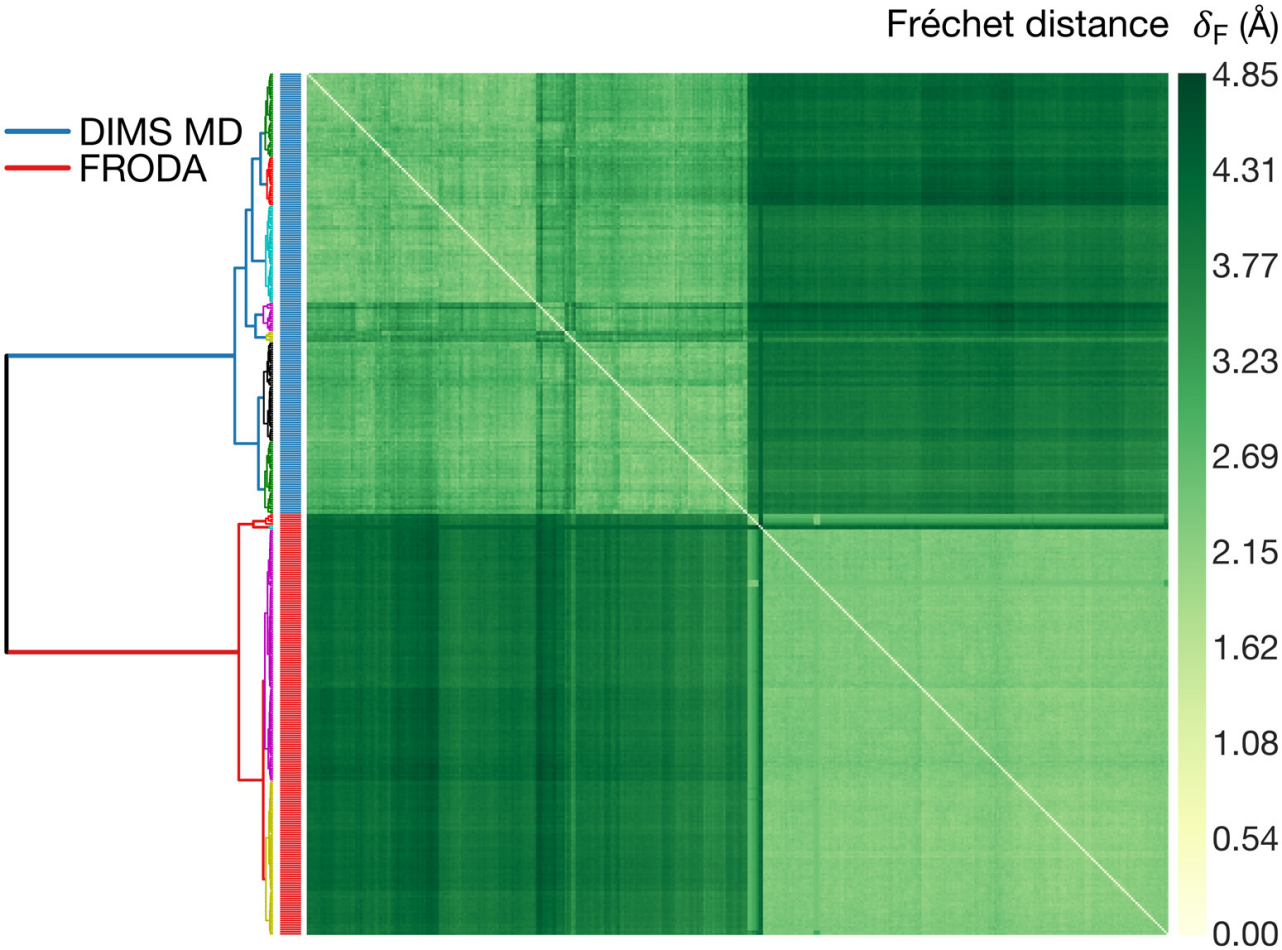
Clustered heat map comparing ensembles of diphtheria toxin (1MDT to 1DDT) transition pathways produced by DIMS (blue bars) and FRODA (red bars) using the Fréchet distance *δ*_*F*_. All clusterings are produced using the Ward’s criterion in ascending distance order; incomplete trajectories were filtered and not displayed (see text).

While the AdK analysis was relatively straightforward, the DT heat map immediately revealed nine erroneous FRODA trajectories producing Fréchet distances upwards of 5 Å from any other path (see S9 Fig for the original clustering). Erroneous paths were removed by specifying a distance cutoff and re-clustering using the trimmed FRODA ensemble. Visual inspection of the omitted trajectories confirmed that they either stopped short of the target or that they came somewhat near the target but continued to dramatically wander in its vicinity. All the DIMS trajectories and all of the FRODA trajectories formed two large, separate clusters (Fig 8).

It is not immediately obvious how one would tune one particular path-generating algorithm to increase its likelihood to produce a path characteristic of another algorithm, although we already observed that the variation of the pulling speed in the rTMD method led to quantitatively (Fig 6) and qualitatively (Fig 7) different paths. In particular, fast pulling (rTMD-F) generated paths similar to linear interpolation (LinInt), whereas slow pulling (rTMD-S) paths were more similar to DIMS and MDdMD. Thus, although we do not yet understand the general relationship between the pathways sampled by different algorithms, PSA appears to be a useful tool to tackle this question.

The ultimate goal is, of course, to find a method that reliably samples transitions realized in the real system. The analyses presented here should also aid in identifying any overlap between different sampling methods and experimental data (e.g. from femtosecond structural biology experiments) when such data become available.

### Hausdorff pairs connect PSA to molecular detail

PSA is a general approach that can operate on the full 3*N*-dimensional trajectories without requiring any system-specific knowledge. It provides a very broad means to categorize transitions as distinct from one other. But as described so far, it is difficult to relate the global PSA analysis to physically relevant differences at the molecular level. To address this question we introduce the new concept of “Hausdorff pairs” (or “Fréchet pairs”) that allows us to pinpoint conformations that may be more likely to exhibit geometric (structural) features relevant to conformational change.

By construction, the Hausdorff and the Fréchet distances identify a point-wise distance between two particular conformers, one on each path, as the global distance between the paths. The path metrics therefore induce a map between a conformer on one path to a conformer on another whose separation distance is, in some sense, a maximal deviation between the paths. We term such a pair of conformers a *Hausdorff pair* (*δ*_*H*_-pair) or a *Fréchet pair* (*δ*_*F*_-pair). These conformers can be examined at the molecular or atomic level to reveal the specific structural discrepancies that give rise to large deviations in configuration space between pairs of paths.

**Fig 9:**
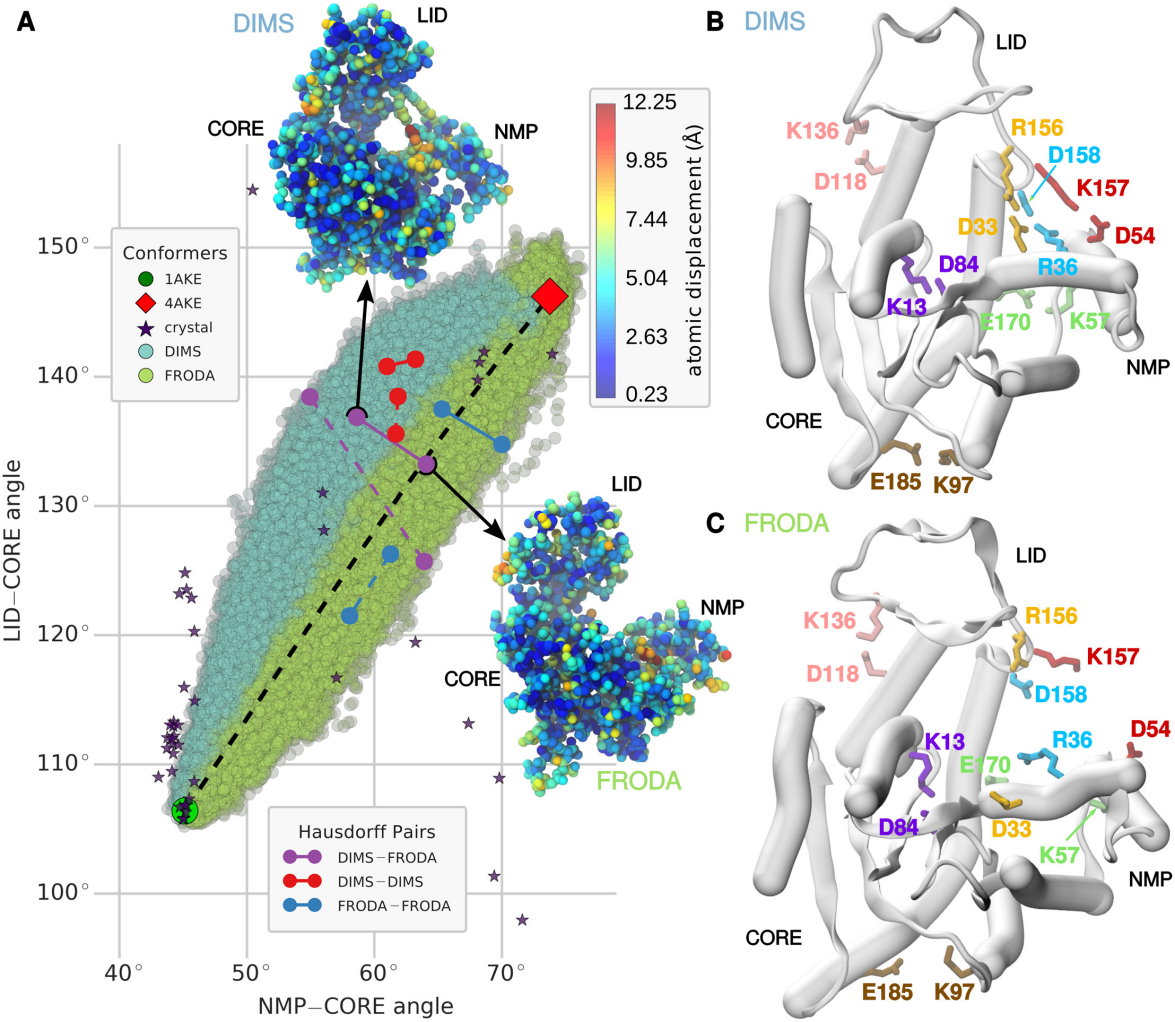
“Hausdorff pairs” (*δ*_*H*_-pairs) analysis using 200 DIMS (cyan) and 200 FRODA (light green) trajectories projected into AA space. Hausdorff distances were computed for all unique path pairs. (A) Conformer pairs—corresponding to the *δ*_*H*_-pairs with the median and maximum Hausdorff distances (solid and dashed lines, respectively)—are projected onto the domain angle space for the following comparisons: DIMS–FRODA (purple), DIMS–DIMS (red), and FRODA–FRODA (blue). Experimental crystal structures, including some intermediates, are shown as stars [99], with further details available in S1 Table. Insets: Two heavy-atom representations are shown for the median *δ*_*H*_-pair between a DIMS path and FRODA path, corresponding to snapshots from the respective trajectories. The magnitude of the displacement vector between the two conformations is projected onto each atom. Color bar units for atomic displacement are in Ångström. The initial and final conformations (green circle and red diamond, respectively) are shown along with the linear interpolation path LinInt—black dashed line) for reference. (B,C) Salt bridges in the DIMS and FRODA conformers from the DIMS-FRODA median Hausdorff pair. Three LID-NMP salt bridges (R156-D33, D158-R36, and K157-D54) and a CORE-NMP salt bridge (E170-K57) are intact in the DIMS structure (B) that are broken in the FRODA structure (C). The residues responsible for these salt bridges tug on the NMP domain more substantially than their counterparts in the LID domain, which are located toward the base of the LID.

As an explicit example, we identified three Hausdorff pairs for the DIMS and FRODA closed → open AdK transition ensembles and projected them in AA space (Fig 9A). We first segregated the full set of Hausdorff distance measurements into: (1) mutual distances among DIMS paths, (2) mutual distances among FRODA paths, and (3) inter-method distances measured between a DIMS and a FRODA path. A total of *N*(*N*−1)/2 = 79800 *δ*_*H*_-pairs were identified for the ensemble of *N* = 400 paths. In order to present representative data for the whole ensemble, we identified the two *δ*_*H*_-pairs associated with the median and maximum Hausdorff distances for each comparison (1), (2), and (3) as defined above.

As a typical example we explicitly examined the median *δ*_*H*_-pair identified for the inter-method comparisons and projected the atomic displacements onto each structure to locate regions of large deviation (see structures in Fig 9A). It became apparent that the NMP domain in the DIMS structure was closer to the LID domain because a number of evolutionary conserved salt bridges (D33–R156, R36–D158, D54–K157) persisted late into the transition due to the strong electrostatic interaction between the acidic and basic moieties [99] (Fig 9B). FRODA, on the other hand, operating on purely geometric principles and neglecting Coulomb interactions, does not account for the influence of salt bridges on the transition and the associated *δ*_*H*_-pair structure exhibited broken salt bridges in the inter-LID/NMP region (Fig 9C). It is therefore not surprising that the FRODA trajectory did not show the “salt-bridge zipper” [99], which manifested itself as discerning difference between the DIMS and FRODA trajectories. With salt bridges located across the NMP domain but primarily on the side of the LID, the LID is relatively free to move to an open configuration, whereas the NMP domain is prevented from fully opening until the salt bridges are broken. These considerations are consistent with the tendency of DIMS paths to primarily favor a LID-opening pathway (Fig 9A, blue circles), while FRODA paths (Fig 9A, green circles) sampled the region around LinInt (Fig 9A, black dashed line) corresponding to simultaneous LID/NMP-opening.

A Hausdorff pair describes the two frames at which the two trajectories in question differ most. Additionally, the regions where trajectories differ to varying degrees from each other might also be of interest. This kind of information is provided by the set of nearest neighbor distances along a path. Eq 3 defines the *nearest neighbor distance* of point *p*_*k*_ on path *P* from path *Q* as *δ*_*h*_(*k*; *P* ∣ *Q*) ≔ *δ*_*h*_(*p*_*k*_ ∣ *Q*) ≔ min_*q* ∈ *Q*_ *d*(*p*_*k*_, *q*) and the nearest neighbor distance of point *q*_*k*_ on path *Q* from path *P* as *δ*_*h*_(*k*; *Q* ∣ *P*). In general, these two distances are not symmetric, i.e. *δ*_*h*_(*k*; *P* ∣ *Q*) ≠ *δ*_*h*_(*j*; *Q* ∣ *P*) for any conformations *j*, *k*. When *δ_h_*(*k*(*ξ*); *P* ∣ *Q*) and *δ_h_*(*j*(*ξ*); *Q* ∣ *P*) are plotted against a suitable common order parameter *ξ*, the regions of large and small differences between trajectories can be quantified. For example, in S11 Fig, the nearest neighbor distances of the three pairs of trajectories corresponding to the median Hausdorff pairs in Fig 9A showed that the DIMS and FRODA trajectories primarily differed in the first ∼ 60% of the transition, which corresponds to LID-opening in DIMS and simultaneous LID/NMP-opening in FRODA. The DIMS trajectories differed almost uniformly along the whole path by only ⪅ 1.3 Å, suggesting that they follow a similar path perturbed by thermal fluctuations. The FRODA trajectories differed by ∼ 2 Å during the middle half of the transition but practically coincided at beginning and end, showing that FRODA can accurately connect two given endpoint structures even with its stochastic component enabled.

The Hausdorff-pair and nearest neighbor distance analysis naturally followed from the formulation of PSA. Even though only C_*α*_ atoms were used to distinguish DIMS from FRODA trajectories hence the level of detail of PSA was primarily restricted to conformational differences in the protein backbone, atomic-scale analysis of Hausdorff-pairs was able to reveal the molecular determinants responsible for the structural differences.

### Conclusions

#### Summary

We have developed a flexible and quantitative framework for analyzing macromolecular transition paths using path metrics as a means to measure the mutual similarity of paths in configuration space, potentially using the full 3*N*-dimensional configuration space information. As far as we are aware, there is currently no standard procedure for quantitatively analyzing and characterizing transition paths. After comparing a set of transitions from a variety of path-sampling algorithms and analyzing transition ensembles of two sets of dynamical, stochastic trajectories, the viability of PSA (Path Similarity Analysis) as a tool to quantitatively compare transition paths appears promising.

In particular, PSA demonstrated that for the adenylate kinase closed → open transition, fast path sampling methods generated paths that were more similar to other paths produced by the same method than to any other paths, suggesting that among these methods there is currently no real consensus for what a realistic conformational transition looks like. Hierarchical clustering in combination with a heat map representation indicated broad patterns whereby dynamical methods tended to be clustered with each other while non-dynamical ones (especially most of the elastic network-based ones) were more similar to each other than to methods such as dynamic importance sampling (DIMS) or the Framework Rigidity Optimized Dynamics Algorithm (FRODA). The clustering was qualitatively confirmed by a range of low-dimensional projections on collective variables, which can be used synergistically with PSA once additional knowledge about the system of interest is available.

The ensemble analysis also clearly showed that at least for large scale macromolecular transitions, the Fréchet and the Hausdorff distance are equally appropriate measures for path similarity, with the Hausdorff distance being cheaper to compute.

A key advantage of PSA is its generality in that no system specific knowledge is required and all trajectory data can easily be used. That generality might, however, obscure the physical and biological detail that is important for a mechanistic understanding of protein function. The new concept of Hausdorff (or Fréchet) pairs within the context of PSA suggests an effective approach to identifying the molecular determinants responsible for differences in paths between sampling methods, which could be related to variations in protein function, differences in path-sampling algorithms/models, or some combination of both (as demonstrated for the comparison between DIMS and FRODA).

#### Future directions

The rmsd proved a useful choice for the point metric to measure structural similarity as its preponderance in the literature helps to connect path metric distance measurements to familiar intuitions although its interpretation is not entirely obvious across disparate contexts [120]. A further study would, for instance, benefit from a revised definition of native contacts where consecutive alpha carbons (within the cutoff distance) would be ignored. As such, a revised native- or self-contacts measure could be used as a point metric instead of rmsd. We also plan to employ k-medoids clustering—used to identify a “median” element (i.e., a medoid) in a set—as one possible approach to identifying representative transition paths, bringing us a step closer to identifying transition tubes or candidates for reaction coordinates.

Furthermore, PSA can aid the assessment of enhanced path-sampling algorithms and their performance by quantifying the degree of similarity to gold-standard transition paths (for instance, equilibrium MD transitions or—when available—experimental time-resolved structural data). Such quantitative comparisons would be key to assessing the physical plausibility of transitions generated by enhanced sampling methods. However, comparison of such transition paths to true equilibrium paths will require the development of a method to identify the actual transition events in the equilibrium data and to distinguish them from residency in (meta) stable states, ideally without introducing problem-specific order parameters. Possible approaches may include an analysis of the distribution of Hausdorff nearest neighbor distances or discrete Fréchet couplings or techniques to match subtrajectories [121, 122] but identifying barrier crossing events from coordinate trajectories in a general manner alone remains an open problem. By extending the analysis of Hausdorff pairs we should also be able to better pinpoint key structural events or mutations that affect the function of biomolecules and so improve our understanding of the connection between protein structure, dynamics and function.

## Supporting Information

S1 Text Alternative distance functions: average Hausdorff and discrete average Fréchet. This text explores variations of the Hausdorff and discrete Fréchet metrics based on averages rather than maxima, which are intended to reduce sensitivity to outliers. Definitions are provided and are shown, via example, to violate the triangle inequality. We repeat the path-sampling methods comparison and discuss the behavior of these distance functions in the context of PSA.
(PDF)

S2 Text Comments on the numerical implementation of path metrics. We informally discuss and comment on the numerical aspects of computing transition path similarity and the algorithms used to calculate Hausdorff and Fréchet distances.
(PDF)

S3 Text Comments on the selection and validation of clustering algorithms. In this text we mention qualitative and quantitative considerations in selecting the Ward linkage criterion for hierarchical clustering, in the context of other linkage criteria. Some comments on potential data interpretation pitfalls when performing general cluster analyses are provided with a view toward viable approaches to PSA cluster and data validation.
(PDF)

S4 Text Mathematical details for the energetics and dynamics of the double-barrel model. Mathematical details are provided for the simulation of the double-barrel model. The system assumes overdamped Langevin dynamics (Brownian motion) and numerical integration was performed using a first-order scheme in time. The construction of the model permits consistent coarse-graining with respect to the number of particles in a cluster, allowing tuning of the number of degrees of freedom, or the dimensionality of the configuration space.
(PDF)

S5 Text Expanded overview of the transition path generating algorithms used in this study. Here we provide a short review—for reader convenience—of the transition path generating algorithms used in the comparison of sampling methods. We summarize the key aspects of each of the physical models and path generating algorithms to help lay the groundwork for connecting algorithmic/model differences to differences between the respective transition paths that were produced.
(PDF)

S6 Text Details of structural alignment procedures for protein alignment prior to path similarity analysis. This text summarizes the considerations involved in structurally aligning conformer snapshots prior to running path similarity analysis on a set of transition paths. We provide specific details and motivations as to the alignment procedures used for AdK and DT trajectories.
(PDF)

S1 Fig Effect of temperature and dimensionality on the distribution of path metrics. Violin plots [91] show the distributions of discrete Fréchet distances for double-barrel simulations of one particle (orange) and eight particles (purple) for temperatures ranging between 0 and 500 in 50 increments (panels A–K) and at 600 (panel L). Black points correspond to individual Fréchet distance measurements, with distance units in nm rmsd. A kernel density estimate (kde) is shown for each *N*, *T* pair to qualitatively emphasize the behaviors of the distributions across the entire temperature range; the bandwidth for each pair is explicitly set to produce two distinct distributions at low temperatures and gradually increased to generate smooth, single distributions at high temperatures. The separated distributions at low temperatures merge between 300 K to 450 K, with the eight-particle simulations merging toward higher temperatures relative to the one-particle simulations.
(PDF)

S2 Fig Correlation analysis of Fréchet and Hausdorff distances in the toy model. Regression analyses examining the correlation between corresponding Fréchet (horizontal axes) and Hausdorff (vertical axes) distance measurements are plotted along with the joint distributions plots for double-barrel simulations of one particle (orange points) and eight particles (purple) for temperatures ranging between 0 and 500 K in 50 K increments (panels A–K) and at 600 K (panel L). Scatter points correspond to individual Fréchet distance measurements in nm rmsd and are plotted with the line produced by linear regression. The shading about the regression lines correspond to a 95% confidence interval. Kernel density estimates (kde) are shown for each *N*, *T* pair and are computed using the same set of bandwidth constants specified in S1 Fig. The separated distributions at low temperatures merge between 300 K to 450 K, with a notable narrowing of the range of distance measurements occuring between 400 K to 450 K.
(PDF)

S3 Fig Effect of temperature and dimensionality on the correlation between Fréchet and Hausdorff distance. Coefficients of the Pearson correlation between Hausdorff and Fréchet distances for one- and eight-particle simulations plotted as a function of temperature. Path distances remain well correlated up to 300 K and are least correlated at 500 K, with the one-particle simulations exhibiting a substantially larger drop in correlation. At the highest temperature the central barrier becomes negligible and the simulations start to equally sample a single tube dominated by the steep repulsive walls. Therefore, the paths start becoming more similar between the *N* = 1 and *N* = 8 clusters and the correlation coefficient increases.
(PDF)

S4 Fig Temperature-dependent transition from two to one distinct paths in the toy model. The means and standard deviations of the discrete Fréchet (blue) and Hausdorff (red) distances for double-barrel simulations of one particle (A) and eight particles (B) are shown as a function of temperature. Measurements for simulations at 250 K and below were divided into an upper and lower distribution by separating distance measurements above and below a 1.25 nm cutoff. Above the temperature cutoff, all measurements were treated as part of the same distribution. Both the Fréchet and Hausdorff metric lose the ability to distinguish between the two barrels as the paths begin to wander out of well-defined pathways when the temperature is on the order of the equivalent energy of the central barrier (2*k*_*B*_ *T* at 300 K). At higher temperatures, thermal perturbations become large relative to the barrier, permitting particle clusters to explore the full width of the potential spanning both barrels so as to generate trajectories confined to a single, unified pathway.
(PDF)

S5 Fig Annotated Fréchet distance matrix of AdK transition trajectories generated by different path-sampling methods. The Fréchet distance matrix from Fig 6 is shown with the numerical values of *δ*_*F*_ (rounded to one decimal) superimposed. Due to the size of the distance matrix, the high resolution image is provided as a simple means for online data exploration with the help of the zoom function of an image viewer.
(PDF)

S6 Fig Influence of the linkage algorithm on the clustered PSA comparison of different path sampling methods. Different linkage algorithms were used to cluster the Fréchet distances produced by path-sampling methods for the AdK closed → open transition. Smaller distances (in units of Å rmsd) indicate transition paths with greater similarity. Dendrograms for each heat map correspond to the hierarchical clustering produced by the single (A), complete (B), average (C), and weighted (D) linkage algorithms, and depict a hierarchy of clusters with smaller node heights of parent clusters indicating greater similarity between child clusters.
(PDF)

S7 Fig PSA comparison of different path sampling methods based on the Hausdorff distance. (A) Heat map for path-sampling methods for the AdK closed → open transition of Hausdorff distances produced using the Ward algorithm. Clusters are identical to the Ward clustering for Fréchet distances in Fig 6. (B) Correlation and joint distributions between discrete Fréchet versus Hausdorff distance measurements (in Å rmsd) for the AdK closed → open methods comparison. Strong linear correlation indicated by the scatter plot, with a Pearson correlation coefficient very close to unity, indicates that either metric could have been used to perform the path-sampling methods analysis with essentially identical results. A slight deviation of the scatter points below the line of unity slope is consistent with the fact that Fréchet distances are bounded from below by corresponding Hausdorff distances.
(PDF)

S8 Fig Clustered PSA heat map of AdK transition ensembles. Clustered heat map comparing path ensembles of adenylate kinase (1AKE:A to 4AKE:A) transition paths produced by DIMS (red bars) and FRODA (blue bars) using the discrete Fréchet distance *δ*_*F*_ are summarized by heat map cluster analysis. Clustering was produced using the Ward algorithm in ascending distance order.
(PDF)

S9 Fig Clustered PSA heat map of raw DT transition ensembles. Clustered heat map comparing the raw path ensembles of diphtheria toxin (1MDT:A to 1DDT:A) transition paths produced by DIMS (red bars) and FRODA (blue bars) using the discrete Fréchet distance *δ*_*F*_. Clustering was produced using the Ward algorithm in ascending distance order. Nine erroneous FRODA paths (orange cluster) were very distant from all other paths—all nine were removed from the ensemble and to produce the heat map dendrogram in Fig 8.
(PDF)

S10 Fig Correlation between Fréchet and Hausdorff distance in ensemble comparisons. Correlations and joint distributions of discrete Fréchet versus Hausdorff distance measurements (in Å rmsd) of the AdK (A) and DT (B) ensemble analyses are shown. Measurements are divided into three separate distributions: (1) mutual distances among DIMS paths (red), (2) mutual distances among FRODA paths (green), and (3) inter-method distances measured between a DIMS and a FRODA path (blue). Both scatter plots show strong correlation between the path metrics for all the distributions, with Pearson correlation coefficients equal to unity and p-values equal to zero to two decimal places, indicating that either metric could have been used to perform the path-sampling methods analysis to obtain essentially identical results. A slight deviation of the scatter points below the line of unity slope is consistent with the fact that Fréchet distances are bounded from below by corresponding Hausdorff distances. DIMS simulations exhibited less variation than FRODA in the AdK transition, but had a larger average variation in the DT transition. In both cases, inter-method DIMS-FRODA comparisons were substantially larger than comparisons of pairs of paths produced within a single method.
(PDF)

S11 Fig Nearest neighbor distances along trajectories for the median Hausdorff pairs in the AdK ensemble comparison. The nearest neighbor distances *δ*_*h*_(*k*; *Q* ∣ *P*) (solid line, ——) and *δ*_*h*_(*k*; *P* ∣ *Q*) (dashed line, ----) between pairs of paths *P*/*Q* belonging to the three median Hausdorff pairs in the AdK ensemble comparison (Fig 9A) are shown for DIMS/FRODA (purple), DIMS/DIMS (blue), and FRODA/FRODA (green). The largest value max_*k*, *j*_(*δ_h_*(*k*; *Q* ∣ *P*), *δ_h_*(*j*; *P* ∣ *Q*)) is the actual Hausdorff distance. For illustration purposes, nearest neighbor distances are plotted as a function of frame number *k* normalized to the interval [0, 1] (i.e., *k*/∣*P*∣), where 0 (1) corresponds to the first (last) frame. In general, an appropriate one-dimensional order parameter should be chosen in order to plot nearest neighbor distances for structurally corresponding trajectory frames.
(PDF)

S1 Table Numerical values for domain angles for experimental crystal structures of AdK. The NMP-CORE angle and LID-CORE angle for *E. coli* EcoAdK or models of EcoAdK based on crystal structures of homologous proteins [99] was computed from the C_*α*_ atoms in the NMP, LID and CORE domain as described in Methods. The angles are plotted in Fig 9A.
(PDF)
