# Correction: Path Similarity Analysis: A Method for Quantifying Macromolecular Pathways

**DOI:** 10.1371/journal.pcbi.1007136

**Published:** 2019-06-17

**Authors:** Sean L. Seyler, Avishek Kumar, M. F. Thorpe, Oliver Beckstein

## Notice of Republication

This article was republished on June 11, 2018, to correct errors throughout the manuscript that were introduced due to a technical error made during the plotting of the data. The editors confirm the major conclusions of the manuscript remain accurate. Please download this article again to view the correct version.

## Supporting information

S1 File Republished, corrected article. (PDF)
