# Supplementary material for: Path Similarity Analysis: A Method for Quantifying Macromolecular Pathways
Source: PLoS Comput Biol. 2015 Oct 21;11(10):e1004568. doi: 10.1371/journal.pcbi.1004568 (PMC4619321; doi:10.1371/journal.pcbi.1004568)
Supplement: S1 Text — This text explores variations of the Hausdorff and discrete Fréchet metrics based on averages rather than maxima, which are intended to reduce sensitivity to outliers. Definitions are provided and are shown, via example, to violate the triangle inequality. We repeat the path-sampling methods comparison and discuss the behavior of these distance functions in the context of PSA. (PDF) [file pcbi.1004568.s001.pdf]

Supporting Information

S1 Text. **Exploring alternative distance functions: average Hausdorff and discrete average Fréchet**

This text briefly explores variations of the Hausdorff and discrete Fréchet metrics that are based on averages rather than maxima. Straightforward definitions are provided for these average-type path *distance functions*. We walk through an example where they violate the triangle inequality to show that they do not define proper metrics. Both average-type path distance functions are applied in the context of the path-sampling methods comparison (using Ward hierarchical clustering) and their behavior is discussed. Future studies may serve to explore other possible definitions and the extents of their application.

Definitions and implementation

The Hausdorff and discrete Fréchet metrics are both sensitive to path outliers—even when the majority of points in two paths are spatially proximate, a single point that deviates substantially can generate large path distance measurements. Indeed, Hausdorff and discrete Fréchet may not be suitable for calculating an "overall" similarity along the entire lengths of two paths. There exist many possible definitions for path distance functions based on measures of central tendency, such as variations based on average or median distances; we consider two variations of the Hausdorff and discrete Fréchet distance functions based on averages rather than maxima.

We recall that a metric must satisfy the following properties:

$$\delta(A,B) \geq 0 \tag{1a}$$

$$\delta(A,B) = 0 \iff A = B \tag{1b}$$

$$\delta(A,B) = \delta(B,A) \tag{1c}$$

$$\delta(A,C) \leq \delta(A,B) + \delta(B,C). \tag{1d}$$

A *distance function* may be said to satisfy the first three properties, whereas the triangle inequality (Eq. 1d) may be violated. Below, we provide explicit definitions for average-type Hausdorff and discrete Fréchet distance functions and show that they are *not* metrics using an example where the triangle inequality is not satisfied.

Average Hausdorff distance

We define the average Hausdorff distance between two paths to be the sum of the nearest neighbor distances for all points (on both paths) divided by the total number of points. We define the one-sided summed Hausdorff distance from path P to path Q as

$$\delta_h^{\text{sum}}(P \mid Q) = \sum_{p \in P} \min_{q \in Q} d(p,q), \tag{2}$$

so that the (symmetric) average Hausdorff distance is the total sum normalized by the total number of points:

$$\delta_H^{\text{avg}}(P,Q) = \frac{1}{|P|+|Q|}\left[\delta_h(P \mid Q) + \delta_h(Q \mid P)\right], \tag{3}$$

where $|P|$ and $|Q|$ are the cardinalities of (the number of points comprising) P and Q, respectively. A similar definition was examined by Eiter and Mannila [1], the only difference being that they normalized by the number of paths ($\frac{1}{2}$) rather than the total number of points in the paths ($\frac{1}{|P|+|Q|}$). A sensible alternative is a weighted average Hausdorff distance,

$$\delta_H^{\text{wavg}}(P,Q) = \frac{1}{2}\left[\frac{1}{|P|}\delta_h(P \mid Q) + \frac{1}{|Q|}\delta_h(Q \mid P)\right], \tag{4}$$

which normalizes the contribution of each one-sided sum so that each path contributes equally irrespective of the number of constituent points. This prevents paths with relatively many points from "diluting" the overall average when compared to those with fewer points. Weighted average Hausdorff should thus minimize sensitivity to the number of points used to parametrize a path, whereas the average Hausdorff distance will tend to discount distance contributions from paths with relatively few points.

In Eq. 3, the average is over $|P| + |Q|$ total points, while in Eq. 4, separate averages over $|P|$ and $|Q|$ points are averaged together with equal weights. These definitions are consistent with the usage of "average" and "weighted average" for hierarchical clustering linkages (see S2 Text). For brevity, we focus on the weighted average Hausdorff distance (Eq. 4) in this text.

Discrete average Fréchet distance

As with average-type Hausdorff distances, several definitions for a discrete average Fréchet distance function are possible. We use a definition identical to that employed by Dickson et al. [2] where the coupling distance is defined as the average link length (in the coupling), which is defined in the subsequent discussion.

Following the description of the conventional discrete Fréchet distance in the main paper, we consider two polygonal curves P and Q, each with n and m ordered points (respectively), in a metric space (V,d) for some metric d. The sequence of line segments of P and Q are respectively defined as $\sigma(P) = (p_1, \ldots, p_n)$ and $\sigma(Q) = (q_1, \ldots, q_m)$. The coupling (in the product space $\sigma(Q,P) \equiv \sigma(P) \times \sigma(Q)$) between P and Q is

$$C(P,Q) \equiv (p_{a_1}, q_{b_1}), (p_{a_2}, q_{b_2}), \ldots, (p_{a_L}, q_{b_L}), \tag{5}$$

of L unique pairs of points (i.e., number of links) and satisfies the following conditions: (1) The first/last pairs correspond to the first/last points of the respective paths ($a_1 = b_1 = 1$, $a_L = n$ and $b_L = m$); (2) at least one point on either of the paths must be advanced to its successive point, i.e., ($a_{i+1} = a_i$ and $b_{i+1} = b_i + 1$) or ($a_{i+1} = a_i + 1$ and $b_{i+1} = b_i$) or ($a_{i+1} = a_i + 1$ and $b_{i+1} = b_i + 1$) for all $i = 1, \ldots, L$.

The definitions up to this point are identical to conventional Fréchet; however, the coupling distance, C, is now defined as an average distance over all pairs of points in a coupling:

$$\|C\| \equiv \frac{1}{L}\sum_{i=1}^{L} d(p_{a_i}, q_{b_i}). \tag{6}$$

We now consider, as usual, the set of all possible couplings between P and Q, $\Gamma_{P,Q}$, and take the *discrete average Fréchet distance* between P and Q to be the minimum coupling distance:

$$\delta_{dF}^{\text{avg}}(P,Q) = \min_{C \in \Gamma_{P,Q}} \|C\|. \tag{7}$$

Since the average is taken over the number of links in the coupling, the normalization factor for two paths depends not only on the numbers of points in the paths, but the optimal coupling between them. We can generate a coupling between P and Q with the possible fewest links by jumping simultaneously along both paths ($a_{i+1} = a_i + 1$ and $b_{i+1} = b_i + 1$) until an end point on one path is reached, then stepping along the rest of the points on the other path. Quantitatively, we have

$$L_{\min} = \min\{|P|, |Q|\} + |(|P| - |Q|)| = \max\{|P|, |Q|\}. \tag{8}$$

On the other hand, the maximum number of links is generated when a step is taken on only one path at a time, i.e., no simultaneous jumps occur ($a_{i+1} = a_i$, $b_{i+1} = b_i + 1$ or $a_{i+1} = a_i + 1$ and $b_{i+1} = b_i$):

$$L_{\max} = |P| + |Q| - 1. \tag{9}$$

In general, the normalization factor for discrete average Fréchet will be smaller than that for average Hausdorff, and greater than or equal to the number of points in the larger of two paths:

$$\max\{|P|, |Q|\} \leq L \leq |P| + |Q| - 1. \tag{10}$$

Are these distance functions also metrics?

Motivated by Buchin's examination of summed and average Fréchet distances [3], we construct three polygonal paths whose mutual average Hausdorff and discrete average Fréchet distances violate the triangle inequality. We consider three paths P, Q, and R whose direction of traversal along each path is from left to right

Average Hausdorff violates the triangle inequality

In Fig. 1, we consider the nearest neighbor distances for each pair of paths among P, Q, and R. As with the usual Hausdorff distance, the average Hausdorff distance, δ_H^{wavg}, will be invariant to the ordering of points. Following the illustration in Fig. 1A, the distance between P and Q, $\delta_H^{\mathrm{wavg}}(P, Q)$, is computed by considering the nearest neighbors in Q for all points in P and those in P for all points in Q.

Procedure:

1. For each point in P, locate its nearest neighbor (the nearest point) in Q and record the distance.

2. Compute the average nearest neighbor distance over all points in P, normalizing by the number of points in P (five).

3. Repeat the process for Q (same as in previous step by symmetry).

4. Average the two average nearest neighbor distances for P and Q to compute the (weighted) average Hausdorff distance.

Summing the distances explicitly, we have

$$\delta_H^{\mathrm{wavg}}(P, Q) = \frac{1}{2}\left[\frac{1}{5}(2l + l + 2l + l + 0) + \frac{1}{5}(2l + l + 2l + l + 0)\right] = \frac{6}{5}\,l. \tag{11}$$

The distance between P and R is calculated analogously from Fig. 1B:

$$\delta_H^{\mathrm{wavg}}(P, R) = \frac{1}{2}\left[\frac{1}{5}(l + 0 + l + 0 + 0) + \frac{1}{6}(0 + 0 + \frac{l}{2} + \frac{l}{2} + 0 + l)\right] = \frac{11}{30}\,l. \tag{12}$$

By symmetry, we also have $\delta_H^{\text{wavg}}(P,R) = \delta_H^{\text{wavg}}(Q,R)$, so we have the relationship

$$\frac{11}{30}l + \frac{11}{30}l < \frac{6}{5}l \tag{13}$$

$$\delta_H^{\text{wavg}}(P,R) + \delta_H^{\text{wavg}}(Q,R) < \delta_H^{\text{wavg}}(P,Q), \tag{14}$$

which does not satisfy the triangle inequality. It can be easily shown that the (unweighted) average Hausdorff distance (Eq. 3) also violates the triangle inequality for the paths in Fig. 1.

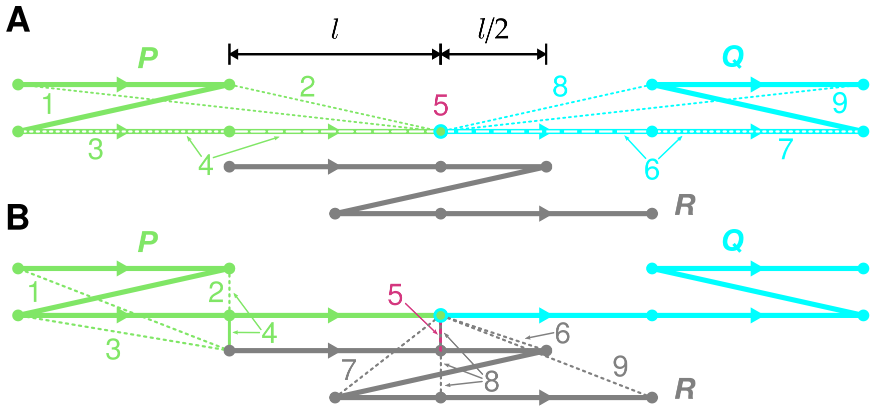

Figure 1: Computing the average Hausdorff distance between three discretized paths P, Q, and R. The vertical direction is expanded for the purpose of illustration and does not represent actual separation; each curve is imagined to lie on the same horizontal axis. Numbered points have a corresponding dashed line representing their nearest neighbor (on the other path). The average Hausdorff distance is computed by averaging the horizontal lengths of dashed lines of a given color and then averaging the two averages for a given pair of paths. (A) Nearest neighbors depicted for P (green) and Q (cyan). (B) Nearest neighbors depicted for P and R (gray).

Discrete average Fréchet violates the triangle inequality

The schematic in Fig. 2 explicitly shows the links comprising the optimal couplings between path pairs among P, Q, and R. As with the usual Fréchet distance, the discrete average Fréchet distance, δ_F^{avg}, is sensitive to the ordering of points; arrows on the paths indicate directionality. The illustration in Fig. 2A depicts the sequence of links (i.e., coupling) with the minimal average link length; the distance between P and Q, $\delta_F^{\text{avg}}(P,Q)$, is computed from the depicted coupling.

Procedure:

1. Begin at the starting (leftmost) points in P and Q connected by the first link.

2. Step along P only (staying at the initial point on Q) until the fifth link (shown in magenta, which has zero length) is reached.

3. Movement along P is completed; step along Q to its last point.

4. Compute the average link length—sum the lengths of all links (in the coupling) and divide by the total number of links—to compute the discrete average Fréchet distance.

Averaging the link lengths explicitly, we have

$$\delta_F^{\text{avg}}(P,Q) = \frac{1}{9}(2l + l + 2l + l + 0 + l + 2l + l + 2l) = \frac{12}{9}l. \tag{15}$$

The distance between P and R is calculated from the coupling shown in Fig. 2B; we remark that the second, fourth, fifth, and eighth (vertical) links each have zero length since P, Q, and R all lie on the same horizontal line:

$$\delta_F^{\text{avg}}(P,R) = \frac{1}{9}\left(l + 0 + l + 0 + 0 + \frac{l}{2} + \frac{l}{2} + 0 + l\right) = \frac{4}{9}l. \tag{16}$$

Again by symmetry, $\delta_F^{\text{avg}}(P,R) = \delta_F^{\text{avg}}(Q,R)$, so we have the relationship

$$\frac{4}{9}l + \frac{4}{9}l < \frac{12}{9}l \tag{17}$$

$$\delta_F^{\text{avg}}(P,R) + \delta_F^{\text{avg}}(Q,R) < \delta_F^{\text{avg}}(P,Q), \tag{18}$$

which violates the triangle inequality.

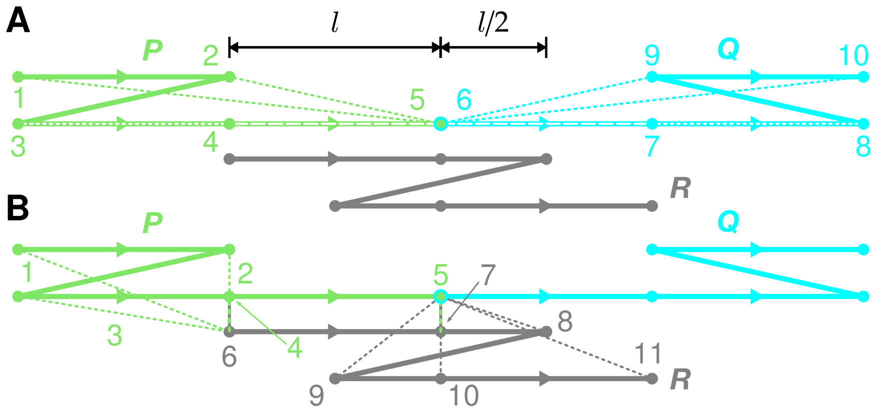

Figure 2: Computing the discrete average Fréchet distance between three discretized paths P, Q, and R. The vertical direction is expanded for the purpose of illustration and does not represent actual separation; each curve is imagined to lie on the same horizontal axis. The (optimal) couplings producing the minimal average link length (i.e., the discrete average Fréchet distance) are shown for P and Q (A), and P and R (B). Links are represented by dashed lines, colored according to the path along which a step is taken, and numbered sequentially for the given coupling; at step 5 in panel (A) and (B), the link length is zero, progress along P is completed, and the remaining movement is solely along the other path.

Summary and additional considerations

Our analyses demonstrate that the average-type Hausdorff distance functions (Eq. 3 and Eq. 4), and the discrete average Fréchet distance (Eqs. 5–7), do not generally satisfy the triangle inequality and are therefore *not* proper path metrics. We note that there may be some problems where the *relaxed* triangle inequality,

$$\delta(A,C) \leq \kappa\left[\delta(A,B) + \delta(B,C)\right], \tag{19}$$

which scales the upper bound on $\delta(A,C)$, normally set by the full triangle inequality, by a finite constant κ, may be sufficient [3]. However, by modifying the paths in Figs. 1 and 2 (by continually increasing the number of “zig-zags” in P, Q, and R), it can be shown that κ becomes arbitrarily large (see Ch. 6 in [3])—the relaxed triangle inequality is also violated by the path distance functions.

While neither PSA nor hierarchical clustering require the use of true metrics, the triangle inequality is a useful property in that it is an intuitive extension of the transitive property. That is, when two objects, A and B, in some metric space are close to a third object, C, in the same space, then A can be considered close to B in the sense that their maximal separation is bounded from above by the

triangle inequality: $d(A,B) \leq d(A,C) + d(B,C)$. In order to preserve commonsense intuition about the pairwise relationships between paths, the main text discusses PSA exclusively in the context of metrics.

Methods comparison using average-type distance functions

Although it was shown above that average Hausdorff and discrete average Fréchet are not metrics, they may still prove to be useful distance functions. We generated heat map dendrograms for the path-sampling methods comparison (using the definitions in Eq. 4 and Eq. 7) to get a feel for their behavior under familiar circumstances.

Weighted average Hausdorff

Average Hausdorff distances substantially smaller than conventional Hausdorff (Fig. 3A), with the largest average Hausdorff distance (2.84 Å) being about 1.5 Å smaller than the largest Hausdorff distance (4.67 Å). In terms of clustering, the primary differences were that both GOdMD and ANMP clustered with the rest of the dynamical methods. The MENM methods formed their own cluster, which was in turn grouped with iENM. DIMS, rTMD-S, MDdMD, and FRODA clustered very similarly to Hausdorff; this was also the case with the rTMD-F, Morph, MAP and LinInt cluster, although Morph groups with MAP instead of rTMD-F in the average Hausdorff heat map. In Fig. 3B, points fall noticeably below the diagonal (of unity slope), indicating that the magnitudes of average Hausdorff distances are both bounded from above by the conventional Hausdorff distance. The Pearson correlation was also weaker (0.868 versus 0.977) and the average Hausdorff distance distribution, though qualitatively similar to that of conventional Hausdorff, is skewed toward smaller values.

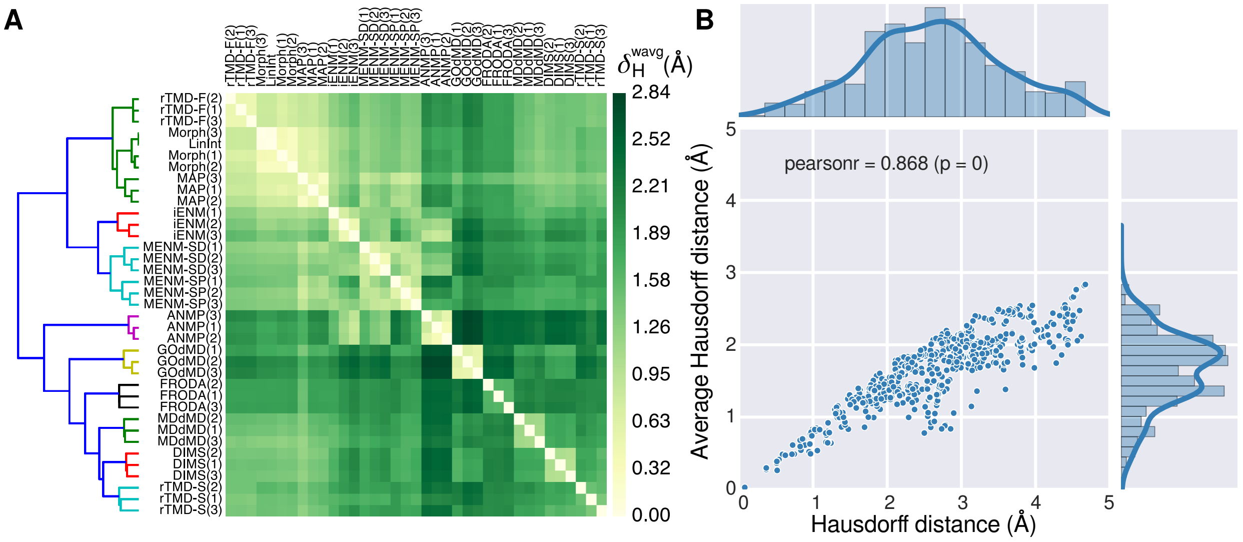

Figure 3: (A) Path-sampling methods comparison for AdK closed → open transition of (weighted) average Hausdorff distances using Ward linkage. (B) Correlation and joint distributions between Hausdorff and (weighted) average Hausdorff distances (in Å rmsd) for the AdK closed → open methods comparison. Reasonably strong linear correlation indicated by the scatter plot, with a Pearson correlation coefficient close to unity.

Discrete average Fréchet

On the other hand, discrete average Fréchet generates a different clustering (Fig. 4A) than the other distance functions and, on the whole, exhibits much smaller distances. GOdMD clustered with the rest of the dynamical methods (with the exception of TMD-F), while all of the elastic network models were grouped along with the cluster of TMD-F, Morph, and LinInt; this is the reverse of what was produced by both Hausdorff distance functions and conventional Fréchet, where GOdMD ended up with the ENMs and the TMD-F, Morph, and LinInt cluster was grouped with the dynamical methods. The majority of points in Fig. 4B fall substantially below the diagonal and are more scattered compared with Fig. 3B; the marginal distribution of discrete average Fréchet closely resembles the distribution produced by average Hausdorff, though the Pearson correlation was slightly stronger (0.892 versus 0.868).

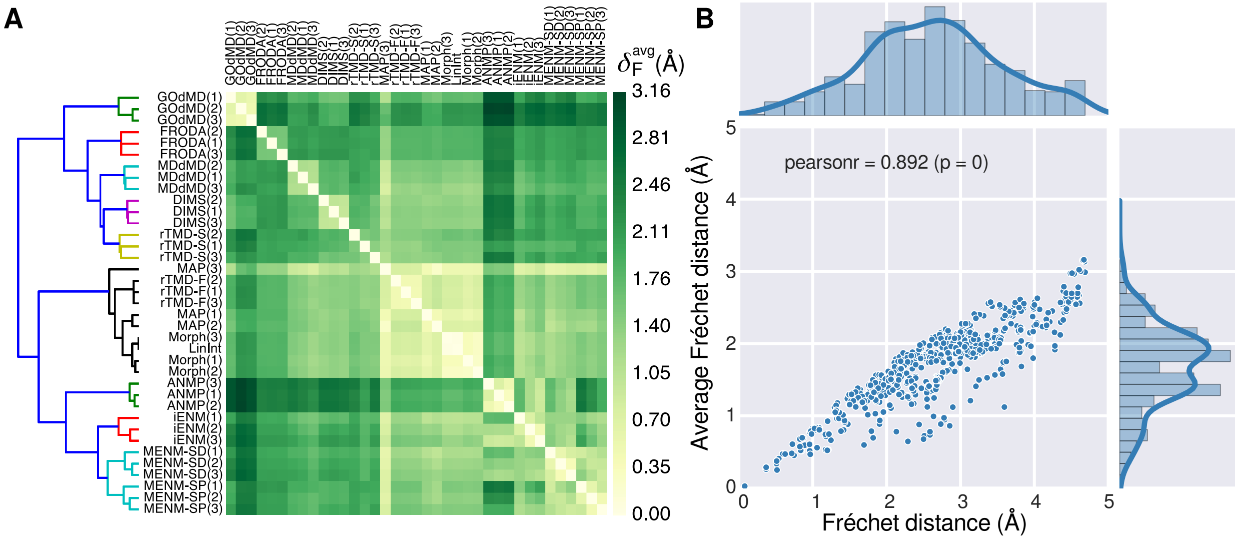

Figure 4: (A) Path-sampling methods comparison for AdK closed → open transition of discrete average Fréchet distances using Ward linkage. (B) Correlation and joint distributions between Fréchet and (weighted) average Fréchet distances (in Å rmsd) for the AdK closed → open methods comparison. Noticeably weaker linear correlation is indicated by the Pearson correlation and the scatter points show that discrete average Fréchet produces distances substantially smaller than conventional discrete Fréchet.

Discussion

From a qualitative standpoint, Figs. 3A and 4A are satisfactory in distinguishing between the most obvious patterns among the path-sampling methods. In particular, both clusterings generate one group containing DIMS, FRODA, MDdMD, and TMD-S, another group with MENM-SD/SP, iENM, and ANMP, and a third group with MAP, Morph, TMD-F, and LinInt. The clustering within each of these groups are also quite similar, with the second and third MENM and iENM paths being closer than the first paths. Interestingly, ANMP clusters with the methods based on dynamical algorithms when using average Hausdorff, whereas average Fréchet places it with the other ENM-based approaches. Meanwhile, the average Hausdorff and discrete average Fréchet distances from the GOdMD paths and MAP(3), to paths from other methods, are substantially smaller than those generated by conventional Hausdorff and Fréchet; the average Hausdorff distances of MAP(3) also correspond to a relatively light band, although it is less pronounced than in the average Fréchet heat map.

In conclusion, the path distance functions based on simple averages (rather than maxima) considered in this supplement are not true metrics as they violate the triangle inequality. We

acknowledge that there may be other path metrics based on measures of central tendency, or at least more robust against outlier points, that would be worth exploring in the future. Furthermore, we did not examine whether the triangle inequality was (or would likely to be) satisfied for a typical path comparison where one may be dealing with a restricted class of curves. Situations may arise where average-type path distance functions behave as metrics for a specific problem. PSA is also not limited to the use of proper path (and point) metrics and it was seen in Figs. 3A and 4A that our average-type Hausdorff and Fréchet distance functions both generated qualitatively acceptable distance measurements. In light of these results, our main study focuses exclusively on the conventional Hausdorff and (discrete) Fréchet metrics because they are satisfactory measures of path similarity that also respect common intuitions about notions of closeness or dissimilarity.

References

[1] Eiter T, Mannila H. Distance measures for point sets and their computation. Acta Inform. 1997;34:109–133.

[2] Dickson BM, Huang H, Post CB. Unrestrained computation of free energy along a path. J Phys Chem B. 2012 13 Sep;116(36):11046–11055.

[3] Buchin M. On the Computability of the Fréchet Distance Between Triangulated Surfaces. Institut für Informatik Freie Universität Berlin; 2007.
