# Supplementary material for: Path Similarity Analysis: A Method for Quantifying Macromolecular Pathways
Source: PLoS Comput Biol. 2015 Oct 21;11(10):e1004568. doi: 10.1371/journal.pcbi.1004568 (PMC4619321; doi:10.1371/journal.pcbi.1004568)
Supplement: S2 Text — We informally discuss and comment on the numerical aspects of computing transition path similarity and the algorithms used to calculate Hausdorff and Fréchet distances. (PDF) [file pcbi.1004568.s002.pdf]

Supporting Information

S2 Text. **Comments on the numerical implementation of path metrics.**

We informally discuss and comment on the numerical aspects of computing transition path similarity and the algorithms used to calculate Hausdorff and Fréchet distances.

Numerical considerations for a transition path similarity measure

To measure the similarity between two paths, we must extend familiar notions of distance (between points) to a distance between ordered *sets of points*, i.e., paths or *curves*, in some metric space. A transition paths is a series of conformer snapshots represented by a sequence of points in $3N$-dimensional configuration space. Formally, a transition path can be defined as a polygonal curve P in a metric space C that has a discrete mapping $P : [0, n] \to C$, where $\{n \in \mathbb{Z} : n \geq 0\}$. If we additionally endow the configuration space with a distance function for conformations (points) in configuration space, then it becomes a metric space (C, d) for point distance metric d, where $d : p \times q \to \mathbb{R}$ and $p, q \in C$ are two conformations (configurations). In this context, d is a structural similarity metric for conformations p and q.

Explicitly define two transition paths, which are polygonal curves $P, Q \in (C, d)$, as explicit conformational sequences, $\{(p_k)_{k=1}^{n} \mid p_k \in \mathbb{R}^{3N}, k = 1, \ldots, n\}$ and $\{(q_k)_{k=1}^{m} \mid q_k \in \mathbb{R}^{3N}, k = 1, \ldots, m\}$. We now seek a reasonable similarity measure between P and Q, i.e., a metric $\delta : P \times Q \to \mathbb{R}$ on C. The Hausdorff, δ_H, and discrete Fréchet, δ_F, distances behave as proper metrics when they are defined in terms of a point metric d on the configuration space C, that is, δ_H and δ_F are path metrics on C when d is also a metric on C.

Outline of the Hausdorff distance calculation

The Hausdorff distance is straightforward to compute numerically as the algorithm follows directly from its mathematical definition. Given an appropriate metric, d, measuring distances between points, the general procedure for measuring the Hausdorff distance between a path P and another path Q with p and q total points, respectively, goes as follows:

1. Locate the first point on path P.
2. Measure the distance to each point in path Q using a point distance function, d.
3. Store the smallest distance from the set of distance measurements obtained in step (2).
4. Move sequentially to the next point in P.
5. Repeat steps (2) through (4) until all points in P have been visited.

6. Repeat the process between steps (1) and (5) with P and Q swapped, so that for each point in Q, find the distance to the nearest point in P.

7. Find the maximum value among all the set of all stored (minimum) distance measurements, which should be a collection of length $p+q$, where p and q are the number of points in P and Q, respectively. This value is defined as the Hausdorff distance.

Alternatively, the set of all unique distances between points in P and points in Q can be represented as a matrix with p rows and q columns, for a total of pq distance measurements. One can then find the Hausdorff distance by calculating minimum distance along each row for all p rows and the minimum for each of the q columns, and then taking the maximum distance among the $p+q$ minima. The computational complexity for computing the Hausdorff distance by either of these approaches is $\mathrm{O}(pq)$. We implemented the algorithm using the NumPy package from the Python programming language.

Comments on calculating the discrete Fréchet distance

Procedures for computing the continuous Fréchet distance are significantly more involving and computationally expensive than discrete Fréchet. As such, we chose to focus explicitly on the discrete Fréchet metric for our analyses. While the continuous Fréchet metric linearly interpolates between consecutive snapshots along a transition path, the discrete Fréchet metric requires that "moves" along a pair of polygonal curves be confined to discrete jumps between successive points—the edges connecting successive points are ignored. Computing the discrete Fréchet distance is somewhat less straightforward than the Hausdorff distance, requiring calculation of a coupling distance for all possible couplings between two paths. We implemented the recursive dynamic algorithm outlined by Eiter and Mannila [1] in Python. For paths P and Q of lengths p and q, respectively, at least pq distances must be computed—redundant calculations are avoided by the dynamic programming procedure [1]—so that the algorithm is $\mathrm{O}(pq)$. While distance calculations can be done quickly due to NumPy vectorization, the recursive part of the routine, used to enumerate all possible couplings between two paths, can make discrete Fréchet calculations substantially slower than Hausdorff calculations when the dimensionality and lengths of the input paths becomes large. Indeed, recursion in Python is expensive and, as the recursion depth grows proportionally to $p+q$, can easily become the bottleneck of the discrete Fréchet algorithm. As currently implemented, the discrete Fréchet algorithm can make ensemble-based analyses or measuring long MD trajectories particularly expensive. Future work will improve the implementation by converting the recursive algorithm to an iterative one.

References

[1] Eiter T, Mannila H. Computing Discrete Fréchet Distance. Wien: Christian Doppler Laboratory for Expert Systems, Technische Universität Wien; 1994.
