# Supplementary material for: Path Similarity Analysis: A Method for Quantifying Macromolecular Pathways
Source: PLoS Comput Biol. 2015 Oct 21;11(10):e1004568. doi: 10.1371/journal.pcbi.1004568 (PMC4619321; doi:10.1371/journal.pcbi.1004568)
Supplement: S3 Text — In this text we mention qualitative and quantitative considerations in selecting the Ward linkage criterion for hierarchical clustering, in the context of other linkage criteria. Some comments on potential data interpretation pitfalls when performing general cluster analyses are provided with a view toward viable approaches to PSA cluster and data validation. (PDF) [file pcbi.1004568.s003.pdf]

Supporting Information

S3 Text. **Comments on the selection and validation of clustering algorithms.**

In this text we mention qualitative and quantitative considerations in selecting the Ward linkage criterion for hierarchical clustering, in the context of other linkage criteria. Some comments on potential data interpretation pitfalls when performing general cluster analyses are provided with a view toward viable approaches to PSA cluster and data validation.

Hierarchical clustering and linkage criteria

Since we use PSA to generate a distance (proximity) matrix (of all path pairs), cluster analysis is directly amenable as a mode of exploratory data visualization. One approach that requires little external input is agglomerative hierarchical clustering, which generates a binary tree (dendrogram) depicting the relationships between objects and clusters.

The similarity between one object (a singleton cluster) and another is specified directly by the distance matrix, while a *linkage* defines a general inter-cluster distance as a function of the pairwise distances of the individual objects (in the clusters). A given linkage will emphasize certain features of the distribution(s) underlying the objects and choosing a good linkage may depend on a confluence of factors. In general, a linkage should generate a clustering that corresponds well with the original distance matrix while highlighting potentially relevant patterns in the data. We summarize below—in the context of transition paths—several linkage algorithms that we applied. The linkages were implemented using the SciPy clustering package and are also described in the SciPy Reference Guide online [1]. Xu et al. [2] also provides alternative definitions and descriptions of the linkages, as well as a general overview of hierarchical clustering.

Ward linkage

Ward's (minimum variance) method is motivated by the statistical analysis of variance between distributions. A candidate object will be grouped with the cluster whose sum of squared errors increases the least upon the object's inclusion [2]. The inter-cluster distance for Ward linkage between clusters u and v, where u is constructed from two sub-clusters, s and t, is

$$d(u,v) = \sqrt{\frac{n(s)+n(v)}{N}d^2(s,v) + \frac{n(t)+n(v)}{N}d^2(t,v) + \frac{n(v)}{N}d^2(s,t)}, \tag{1}$$

where d is the inter-cluster distance, $n(*)$ is the cardinality of (numbers of paths in) cluster $*$, and $N = n(u)+n(v) = n(s)+n(t)+n(v)$ is the total number of paths. The definition is recursive in that the distance between two clusters is defined in terms of distance between their constituent clusters.

The first two terms under the square root give the fractional contributions of paths in sub-clusters s and t to the overall distance. The third term is a distance "penalty" that is proportional to the relative size of v, $n(v)/N$, and the "spread" of u, $\delta^2(s,t)$, which is the squared distance between its sub-clusters. Under Ward linkage, the clustering procedure, which during each pass seeks the two clusters with the smallest distance, will tend to agglomerate small, compact clusters [2, 3].

Other linkages

Single linkage. Also called the Nearest Point Algorithm, single linkage defines the distance between two clusters of paths as

$$d(u,v) = \min\left(\delta(u_i, v_j)\right), \tag{2}$$

u and v, and u_i and v_j are the ith and j^{th} paths in clusters u and v, respectively.

Complete linkage. Similar to single linkage, complete linkage (Farthest Point Algorithm) defines inter-cluster distance as

$$d(u,v) = \max\left(\delta(u_i, v_j)\right). \tag{3}$$

Average linkage. Also called the Unweighted Pair Group Method Average (UPGMA), average linkage defines the distance between clusters as

$$d(u,v) = \frac{1}{n(u)\cdot n(v)} \sum_{i=1}^{n(u)} \sum_{j=1}^{n(v)} \delta(u_i, v_j), \tag{4}$$

In other words, the distance between u and v is the average distance between a path in u and a path in v. Average linkage can also be defined recursively in terms of two sub-clusters, s and t, comprising u:

$$d(u,v) = \frac{1}{n(s)+n(t)} \left[n(s)d(s,v) + n(t)d(t,v)\right]. \tag{5}$$

The contributions of s and t to the distance, $d(u,v)$, between u and v are proportional to their relative sizes so that each path contributes equally to the cluster distance, d.

Weighted average linkage. The Weighted Pair Group Method Average (WPGMA) or *weighted average* linkage is defined recursively as

$$d(u,v) = \frac{1}{2}\left[d(s,v) + d(t,v)\right]. \tag{6}$$

Weighted average linkage gives equal importance to sub-clusters s and t, regardless of the number of paths in each. Thus, if s contains half the number of paths in t, a path in s will have twice the weight as a path in t in contributing to the inter-cluster distance, $d(u,v)$.

Examining cluster quality

Although analyzing in depth the consequences of using different linkages was out of the scope of this study, we produced four additional Fréchet heat map dendrograms (see Fig. S5) for the path-sampling methods analysis using the single, complete, average, and weighted average linkages to perform a basic comparison. The structure of each dendrogram is a function of the linkage algorithm, although it can be difficult (and tricky) to draw strong conclusions about the underlying data.

Quantitative measures

The *cophenetic distance* is a measure of correlation between the original distance matrix and the distances between objects according to the inter-cluster distances assigned by a given linkage. To obtain an approximate measure of the quality of cluster divisions, we examined the *inconsistency coefficients* for clusters containing more than two children (i.e., ignoring singleton clusters and clusters with vanishing inconsistency coefficients). We also computed the maximum inconsistency coefficients for each linkage, where the coefficient for a given cluster is the largest of the coefficients between itself and its children [1, 3].

We computed cophenetic distances for the clusterings produced by each linkage. To concisely examine the inconsistency coefficient data, we averaged the (nonzero) values for each linkage; we likewise computed the average of the maximum inconsistencies for each linkage. There were eleven clusters with inconsistency coefficients of zero among the non-singleton clusters, which were excluded from both averages. The three values computed for each linkage are summarized in Table 1.

Table 1. Summary of the computed clustering-quality measures for each linkage for the methods comparison of Fréchet distances.

Linkage	Cophenetic Distance	Inconsistency Coefficient Statistics	
		Average*	Maxima average†
Ward	0.78	0.86	0.93
single	0.85	0.75	0.80
complete	0.82	0.88	0.92
average	0.85	0.85	0.91
weighted	0.86	0.83	0.91

A cophenetic distance near zero indicates that the overall clustering poorly reflects the actual pairwise distances between the paths, while values close to unity indicate a plausible—but not necessarily good—clustering. All linkages produced clusterings with adequate correspondence. A large inconsistency coefficient indicates that the height of the link corresponding to a cluster is large compared to the average link heights of its children, which in turn indicates that the two child clusters joined at this level are dissimilar. Clusters (links) having larger inconsistency coefficients have more distinct child clusters.
*Average of the nonzero inconsistency coefficients computed for non-singleton clusters.
†For each non-singleton cluster along with its descendents, find the maximum inconsistency coefficient, take the average of the nonzero maxima.

Selecting a linkage criterion

Inter-cluster distances will tend to be smaller for a single linkage than a complete linkage, since the former defines inter-cluster distance as the minimum (rather than maximum) inter-point distance. As a result, fluctuations due to measurement uncertainty will be relatively larger for single linkage, making it more susceptible to noise and a "chaining" effect that can produce stretched clusters [2]; the single linkage (see Fig. S5A) in the methods comparison produced a large (yellow) cluster resembling this effect. The other four (Ward, average, weighted average, complete), on the other hand, generated qualitatively viable clusterings. We note that the average and weighted average linkages merged MDdMD with the Morph/MAP cluster, then merged the result with DIMS, then finally again with FRODA, while the other ENM methods still formed their own cluster. The complete linkage, however, while having placed MDdMD and DIMS in their own cluster, merged FRODA with the ENM methods! It is difficult at this point to determine why FRODA transitions are subject to such different clusterings, although It appears that FRODA paths, being somewhat uniformly different from all others, are subject to being clustered by their dissimilarity, in some

sense, rather than their similarity to other paths.

To decide which linkage is “best” at this stage corrupts the purpose of exploratory data analysis. A more productive view is that each linkage emphasizes different aspects of the data and can be used to tease out specific types of information. A sound general approach is likely to involve the integration of results from a variety of linkages as a means to identify common features and patterns. If a particular cluster, say, Morph/MAP/LinInt, were to appear as a motif across several linkages, it would be a more robust indication that its constituent paths were truly similar (relative to other paths). This approach can be expanded to include other clustering algorithms, such as partitional clustering approaches (e.g., the K-means algorithm).

In light of the previous discussion and the quantitative results in Table 1, the Ward, complete, average, and weighted average linkages perform adequately for the purposes of this study. Both Ward and complete linkage produced particularly well-defined clusters, which was reflected by their overall large inconsistency coefficients. Arguments can easily made in favor of the average-type linkages as well. To keep the focus of the study on the presentation of PSA, we elected to concentrate on one—the Ward linkage–for clarity of presentation.

References

[1] Jones E, Oliphant T, Peterson P, et al.. SciPy: Open source scientific tools for Python; 2001–. [Online; accessed 2015-05-13]. Available from: `http://www.scipy.org/`.

[2] Xu R, Wunsch D. Clustering. IEEE Press Series on Computational Intelligence. John Wiley & Sons; 2008.

[3] Statistics and Machine Learning Toolbox: User’s Guide (R2015a). Natick, Massachusetts: The MathWorks; 2015.
