# Supplementary material for: Path Similarity Analysis: A Method for Quantifying Macromolecular Pathways
Source: PLoS Comput Biol. 2015 Oct 21;11(10):e1004568. doi: 10.1371/journal.pcbi.1004568 (PMC4619321; doi:10.1371/journal.pcbi.1004568)
Supplement: S4 Text — Mathematical details are provided for the simulation of the double-barrel model. The system assumes overdamped Langevin dynamics (Brownian motion) and numerical integration was performed using a first-order scheme in time. The construction of the model permits consistent coarse-graining with respect to the number of particles in a cluster, allowing tuning of the number of degrees of freedom, or the dimensionality of the configuration space. (PDF) [file pcbi.1004568.s004.pdf]

Supporting Information

S4 Text. **Mathematical details for the energetics and dynamics of the double-barrel model.**

Mathematical details are provided for the simulation of the double-barrel model. The system assumes overdamped Langevin dynamics (Brownian motion) and numerical integration was performed using a first-order scheme in time. The construction of the model permits consistent coarse-graining with respect to the number of particles in a cluster, effectively allowing tuning of the number of degrees of freedom, or the dimensionality of the configuration space.

Simulating dynamics in the double-barrel model

In the double-barrel model, we consider the dynamics of clusters of particles in an external potential energy landscape. A cluster of size N (an N-cluster) is a collection of N identical, interconnected particles of mass m, where each particle interacts with the other $N-1$ particles through springs with a force constant k. To produce a simple model, we assume that (1) particles in a cluster only interact through their springs, (2) particles in a cluster are identical smooth spheres moving in the Stokes' flow regime of a Newtonian fluid of uniform viscosity, and (3) each particle obeys a Langevin equation in the overdamped regime (Brownian motion).

We use assumption (1) to neglect complicated particle-particle interactions, such as hard-sphere potentials. Using assumption (2), individual particles are subject to identical viscous Stokes' flow drag forces via immersion in a uniform solvent bath. Under all three assumptions, all particles in a cluster are then subject to the same (Brownian) dynamics, where each particle interacts identically with a solvent acting as a thermal bath, and where inter-particle interactions enter only through the external force term in Langevin equation. All particles in an N-cluster thus have identical collision frequencies and, therefore, friction/diffusion coefficients. Furthermore, assumption (3) implies that the dynamics are Markovian, so that particles do not perturb the velocity of the surrounding fluid, and are thus not subject to memory effects due to the motion of nearby particles—the model ignores interactions between particles through the solvent bath.

Particle dynamics

Individual particles within a cluster were subject to the equation of motion for Brownian dynamics [1],

$$\dot{\mathbf{r}}_i = \frac{1}{\zeta}\boldsymbol{\nabla}_i U(\mathbf{r}(t)) + \hat{\mathbf{W}}_i(t), \tag{S3.1}$$

where $\mathbf{r} = (\mathbf{r}_1, \ldots, \mathbf{r}_N)$ is a $3N$-dimensional vector of all particle positions, $\mathbf{r}_i$ is the position of particle i, $-\boldsymbol{\nabla}_i U(\mathbf{r}(t)) = -\partial U(\mathbf{r})/\partial \mathbf{r}_i$ is the non-thermal force on particle i as a function of all

particle positions at time t, ζ is the viscous damping constant, and $\hat{\mathbf{W}}(t)$ is a stochastic process. The stochastic term, $\hat{\mathbf{W}}(t)$, is a delta-correlated, zero-mean, stationary Gaussian process,

$$\left\langle \hat{W}_\alpha(t)\,\hat{W}_\beta(\tau)\right\rangle = 2D\delta_{\alpha\beta}\delta(t-\tau), \tag{S3.2}$$

where $D = k_B T/\zeta$ is the diffusion coefficient, and α and β run over all spatial dimensions.

Numerical simulation

The trajectory of particle i in a cluster was generated using a first-order integration scheme (cf. Ch. 3 of the GROMACS Manual [1]),

$$\mathbf{r}_i^{n+1} = \mathbf{r}_i^n + \frac{\Delta t}{\zeta}\boldsymbol{\nabla}_i U(\mathbf{r}^n) + \sqrt{2D\Delta t}\,\hat{\mathbf{G}}_i^n, \tag{S3.3}$$

where a superscript n denotes a time $t = n\Delta t$, and $-\boldsymbol{\nabla}_i U(\mathbf{r}^n)$ is the non-thermal force on particle i at time step n; $\hat{\mathbf{G}}_i^n$ is a random variable representing the nth realization of the stochastic process—for each spatial component of particle i at each time step n, it was generated with a Gaussian random number generator having zero mean and unit variance:

$$\left\langle \hat{W}_{i,\alpha}^n\,\hat{W}_{i,\beta}^m\right\rangle = 2D\Delta t\left\langle \hat{G}_{i,\alpha}^n\,\hat{G}_{i,\beta}^m\right\rangle = 2D\Delta t\,\delta_{\alpha\beta}\delta_{nm}, \tag{S3.4}$$

where α and β run over all spatial dimensions, and n and m are the nth and m^{th} time steps, respectively.

Double-barrel potential energy landscape

It is prudent to reiterate that the double-barrel model's purpose is to generate "large-scale" transitions that are induced by an external ramp potential, particularly so that certain aspects of path similarity analysis (PSA) can be examined. The total potential energy in Eq. (S3.3) consists of an external potential and particle interaction terms: $U = U^{(\text{ext})} + U^{(\text{int})}$, respectively. The external potential is broken into a ramp potential and double-barrel potential, where, for particle i (in a cluster), located at position $\mathbf{r}_i \doteq (x_i, y_i, z_i)$,

$$U^{(\text{ext})}(\mathbf{r}_i) = U_{\text{ramp}}(\mathbf{r}_i) + U_{\text{barrel}}(\mathbf{r}_i) \tag{S3.5}$$

$$U^{(\text{ramp})} = F\,z_i, \tag{S3.6}$$

$$U^{(\text{barrel})} = Ax_i^2 + B\,y_i^2\left(y_i^2 - 2C^2\right), \tag{S3.7}$$

where A controls the strength of confinement in the x-direction, B and C control the shape of the barrels with $y = \pm C$ defining the barrel minima in nanometers, and F dictates the ramp steepness or transition force. The inter-particle potential is modeled by Hookean springs:

$$U^{(\text{int})}(\mathbf{r}) = \frac{1}{2}\frac{k_s}{2}\sum_{j\neq i}^{N}(\mathbf{r}_i - \mathbf{r}_j)^2\,, \tag{S3.8}$$

k_s is the inter-particle spring constant.

The ramp potential generates directed progress along the z-coordinate that replicates a large-scale transition. (Note that although these transitions were produced by forcing center-of-mass translation in the positive z-direction, one should keep in mind that real transitions do not necessarily correspond to center-of-mass translations of the system, though they may be present.) The double-barrel potential operates as a means to produce two distinct pathways at sufficiently low temperatures. The inter-particle springs are independent of the coarse-graining level and serve to confine particles into compact clusters.

Conditions and parameter selection for consistent coarse-graining

As PSA can take advantage of the full configuration space, the double-barrel model was designed in part to test PSA when a system is coarse-grained to reduce the degrees of freedom. Transitions generated by the double-barrel model should therefore be consistent in some sense across clusters of varying size. To achieve a reasonable coarse-graining scheme, (1) the transition rate and (2) net diffusivity of a cluster should be independent of its size. In particular, condition (1) demands that if all particles in a cluster are initialized at precisely the same location under zero-temperature conditions—so they all move identically under the external potential—they must transition at a rate independent of the number of particles in the cluster.

As discussed above, we assume all particles in an N-cluster are identical spheres subject to Stokes' flow in the same solvent bath. The Stokes' (viscous) drag force on a particle of radius r in a fluid of dynamic viscosity η is

$$F_{\text{drag}} = 6\pi\eta r U_{\infty}, \tag{S3.9}$$

where U_{∞} is the uniform (far-field) velocity of the surrounding fluid [2], which we treat as unperturbed by neighboring particles. For the above fluid at temperature T, the Einstein relation gives the diffusion coefficient,

$$D = \frac{k_B T}{m\gamma} = \frac{k_B T}{\zeta} = \frac{k_B T}{6\pi\eta r}, \tag{S3.10}$$

in terms of, γ, the collision frequency, and $\zeta = 6\pi\eta r$, the viscous damping coefficient [3, 4]. The last equality in Eq. (S3.10) follows directly from Eq. (S3.9). In the analysis that follows, we consider an N-cluster, where each particle, i, is subject to the dynamics in Eq. (S3.1) in the cases of zero and finite temperature.

Constraints on the friction and potential from zero-temperature dynamics

We first examine conditions for constancy of the transition rate at zero temperature given identical initial conditions of all particles in an N-cluster (i.e., $\mathbf{r}_i(t) = \mathbf{r}^0$ for $i = 1, \ldots, N$). Under these conditions, the stochastic term vanishes and Eq. (S3.1) reduces to

$$\dot{\mathbf{r}}_i = \frac{1}{\zeta}\boldsymbol{\nabla}_i U(\mathbf{r}^0). \tag{S3.11}$$

Since a transition takes place by traversing a set length of the barrels along the z-coordinate, the transition rate depends only on the z-component of the velocity in Eq. (S3.11). Furthermore, the velocity will depend only on the ratio of the magnitude of the gradient of the external potential and the friction coefficient, ζ. Thus, under coarse-graining, we are permitted to change ζ and U for the CG particle so long as they are scaled by the same factor.

One may appeal to the necessity of conserving mass under coarse-graining so that the CG particle mass is the sum of the masses in the N-cluster. In this case, the friction coefficient of the CG particle should be N-times larger than those of the individual N-cluster particles. However, the diffusion coefficient for the CG particle will *decrease* by a factor of N, which will change the dynamics at finite temperature when the stochastic term is non-vanishing. We keep the general analyses above in view before deciding on an appropriate scaling factor for ζ (or, equivalently, D) as we proceed to the finite-temperature case.

Diffusion constraints from finite-temperature dynamics

To derive more general CG constraints at finite temperature, we further require the net diffusive behavior of the CG particle to match its N-cluster. Indeed, if the diffusion coefficient in Eq. (S3.3),

which is proportional to the average squared displacement, is preserved, then the average velocity and, thus, average transition rate will also be preserved.

We consider replacing an N-cluster of total mass M by a CG particle positioned at the center of mass of the cluster, where

$$\bar{\mathbf{r}}(t) = \frac{1}{M}\sum_{i=1}^{N} m_i \mathbf{r}_i(t) = \frac{1}{N}\sum_{i=1}^{N} \mathbf{r}_i(t), \tag{S3.12}$$

is the center of mass, and

$$\dot{\bar{\mathbf{r}}}(t) = \frac{1}{N}\sum_{i=1}^{N} \dot{\mathbf{r}}_i(t) \tag{S3.13}$$

is the center-of-mass velocity. We formulate our constraint on the transition rate to be equivalent to requiring that the diffusion of the CG particle be identical to the net diffusion of center of mass of the N-cluster it replaces.

To see how coarse-graining modifies the dynamics, we take Eq. (S3.1), sum over all particles in the cluster, and divide by N, to obtain the center-of-mass equation of motion for an N-cluster:

$$\dot{\bar{\mathbf{r}}}(t) = \frac{1}{\zeta}\frac{1}{N}\sum_{i=1}^{N} \boldsymbol{\nabla}_i U(\mathbf{r}(t)) + \frac{1}{N}\sum_{i=1}^{N} \sqrt{2D}\,\hat{\mathbf{G}}_i(t), \tag{S3.14}$$

where ζ and D are the friction and diffusion coefficients of each particle, and $\sqrt{2D}\,\hat{\mathbf{G}}_i(t) = \hat{\mathbf{W}}_i(t)$. We can simplify the equation of motion by working individually with each term on the right-hand side.

Potential term. The external potential, $U^{(\mathrm{ext})}$, is a linear function of the positions and takes the same form for each particle, but the inter-particle potential, $U^{(\mathrm{int})}$, includes nonlinear cross terms. To simplify the analysis, we begin by assuming the particles in the N-cluster are initialized at a single point and are rigidly connected. Then $\mathbf{R}(t) = \mathbf{r}_1(t) = \cdots = \mathbf{r}_N(t)$ for all t. We furthermore allow the spring potential to remain constant in the limit as $k_s \to \infty$ and $(\mathbf{r}_i - \mathbf{r}_j)^2 \to 0$ for all i and j. Under these conditions, U and its gradient become linear functions of the positions, so that the force on each particle i,

$$\boldsymbol{\nabla}_i U(\mathbf{r}) = \mathbf{F}_i(\bar{\mathbf{r}}), \tag{S3.15}$$

depends only on the center-of-mass coordinate; the potential term in Eq. (S3.14) then becomes

$$\frac{1}{\zeta}\frac{1}{N}\sum_{i=1}^{N} \boldsymbol{\nabla}_i U(\mathbf{r}) = \frac{1}{\zeta}\frac{1}{N}\sum_{i=1}^{N} \mathbf{F}_i(\bar{\mathbf{r}}) = \frac{1}{\zeta}\,\bar{\mathbf{F}}(\bar{\mathbf{r}}), \tag{S3.16}$$

where $\bar{\mathbf{F}}(\bar{\mathbf{r}})$ is the average of the individual forces acting on the particles, which acts at the center of mass of the N-cluster. We consider scenarios where the particles have arbitrary locations and nonzero inter-particle forces in the subsequent section.

Thermal noise term. The second term on the right hand side of Eq. (S3.14) can be viewed as the average of N realizations of the stochastic process $\hat{\mathbf{G}}_i$. Alternatively, it is the average of the partial sum of N independent, identically distributed (iid) random variables, $\hat{\mathbf{S}}_N = \sum_{i=1}^{N} \hat{\mathbf{G}}_i$. The mean and variance of $\hat{\mathbf{S}}_N$ is the sum of the means and sum of the variances, respectively, of the $\hat{\mathbf{G}}_i$. Furthermore, since the $\hat{\mathbf{G}}_i$ are iid Gaussian random variables, $\hat{\mathbf{S}}_N$ and $\hat{\mathbf{M}}_N = \hat{\mathbf{S}}_N/N$ are also a Gaussian random variables, with zero mean and respective variances N and $1/N$. Using the central limit theorem, we replace the stochastic term in Eq. (S3.14) with a new stochastic process,

$$\hat{\mathbf{M}}(t) = \frac{1}{N}\sum_{i=1}^{N} \hat{\mathbf{G}}_i(t), \tag{S3.17}$$

where

$$\left\langle \hat{M}_\alpha(t)\, \hat{M}_\beta(\tau) \right\rangle = \frac{1}{N} \left\langle \hat{G}_\alpha(t)\, \hat{G}_\beta(\tau) \right\rangle = \frac{2D}{N}\, \delta_{\alpha\beta}\delta(t-\tau). \tag{S3.18}$$

Therefore, the center-of-mass motion of an N-cluster due to N independent, identical stochastic processes acting on the particles is statistically equivalent to a single stochastic process with a rescaled diffusion coefficient, $D^{(\mathrm{com})} = D/N$, acting on the center of mass of the particles.

Coarse-grained equation of motion. By Eq. (S3.18), we can write the stochastic term in terms of $\hat{\mathbf{G}}_i(t)$ with a rescaled diffusion coefficient. Combining this with Eq. (S3.16), the expression for the center-of-mass dynamics of a rigid N-cluster becomes

$$\dot{\bar{\mathbf{r}}}(t) = \frac{1}{\zeta}\, \bar{\mathbf{F}}(\bar{\mathbf{r}}(t)) + \sqrt{2\left(\frac{D}{N}\right)}\, \hat{\mathbf{G}}_i(t). \tag{S3.19}$$

We see that the center-of-mass diffusion is smaller than the individual particle diffusion by a factor of N. If one were to replace the cluster with a CG particle at the center of mass of an N-cluster using the same diffusion coefficient D, as for each of the particles, the CG particle would diffuse too quickly. The CG particle's diffusion coefficient should thus be scaled by N so that $D^{(\mathrm{CG})} = D^{(\mathrm{com})} = D/N$ to preserve the rate of diffusion and, thus, the transition rate.

To maintain the proper relationship between the friction and diffusion coefficients through Eq. (S3.10), the new friction coefficient must also be increased by a factor of N: $\zeta^{(\mathrm{CG})} = N\zeta$. However, the external force must in turn be increased by a factor of N to ensure the external force on the CG particle is the same as the net force on the corresponding N-cluster. Thus, if we begin with an N-cluster under forces $F_i(\mathbf{r})$, with diffusion coefficient D and friction ζ, the equation of motion for the CG particle should be

$$\dot{\mathbf{R}}(t) = \frac{1}{\zeta^{(\mathrm{CG})}} \mathbf{F}^{(\mathrm{CG})}(\mathbf{R}(t)) + \sqrt{2D^{(\mathrm{CG})}}\, \hat{\mathbf{G}}_1(t), \tag{S3.20}$$

where $\mathbf{R}(t)$ is the position of the CG particle at time t, $D^{(\mathrm{CG})} = D/N = k_B T/\zeta^{(\mathrm{CG})}$, and $\mathbf{F}^{(\mathrm{CG})}(\mathbf{R}) = \mathbf{F}_1(\mathbf{R})$ (where we picked $i = 1$ to denote terms identical to quantities for any single particle in the cluster). The new friction coefficient, $\zeta^{(\mathrm{CG})}$, is additionally consistent with the previous consideration of conserving particle mass under coarse-graining:

$$\zeta^{(\mathrm{CG})} = N\zeta = (Nm)\gamma = m^{(\mathrm{CG})}\gamma, \tag{S3.21}$$

where the mass of the CG particle, $m^{(\mathrm{CG})} = Nm = M$, is the sum of the masses of the individual (identical) particles. Furthermore, we see that the collision frequency, γ, can be held fixed across simulations at all levels of coarse-graining.

Deviations from rigid-cluster coarse-grained behavior

It is clear that $\mathbf{F}^{(\mathrm{CG})}(\mathbf{R}) = \mathbf{F}_1(\mathbf{R}) = \bar{\mathbf{F}}(\mathbf{R})$ (averaged over all particles i) in the case where all particles are constrained to the same point for all time. This assumption guarantees the linearity of the potential energy function. Our simulation system on the other hand has finite inter-particle spring constants and an N-cluster will tend to have its constituent particles wandering to some degree. If the single-point, rigid-cluster assumption is relaxed, we can then ask under what conditions the force on the center of mass is well-approximated by the average of the individual particle forces.

If all particles are located at different positions, then they will see different parts of the external potential and will also have nonzero inter-particle forces between them. However, due to Newton's third law these inter-particle forces sum to zero and do not contribute to a force acting on the

center of mass. On the other hand, the sum of the external forces on all particles will *not* generally be equal to the gradient of the external potential evaluated at their center of mass. Now consider replacing an N-cluster with a CG particle that feels N times the force of a regular particle at the same point: $F^{(\mathrm{CG})}(\mathbf{r}^*) = NF_1(\mathbf{r}^*)$. In this case, the net force on the N-cluster (at its center of mass) is, in general, different than the force on the CG particle due to the external potential (at the center of mass):

$$\mathbf{F}^{(\mathrm{CG})}(\mathbf{R}) = -N\boldsymbol{\nabla}_{\mathbf{R}}U^{\mathrm{ext}}(\mathbf{R}) \neq \sum_{i=1}^{N}\mathbf{F}_i(\mathbf{r}) = -\sum_{i=1}^{N}\boldsymbol{\nabla}_i U^{\mathrm{ext}}(\mathbf{r}_i) \tag{S3.22}$$

In particular, Eq. (S3.22) will occur when the external potential U^{ext} has non-vanishing second (or higher) derivatives, i.e., the external force is not a constant function of position.

These statements can be made more precise by first transforming to coordinates relative to the center of mass and writing the positions as

$$\mathbf{r}_i = \Delta\mathbf{r}_i + \mathbf{R}, \tag{S3.23}$$

where $\Delta\mathbf{r}_i$ is the displacement vector of the ith particle relative to the center of mass. The total external force,

$$\mathbf{F}^{(\mathrm{total})} = \sum_{i=1}^{N}\mathbf{F}_i(\mathbf{r}_i) = \sum_{i=1}^{N}\mathbf{F}_i(\Delta\mathbf{r}_i + \mathbf{R}), \tag{S3.24}$$

can then be expressed, for small displacements in $\Delta\mathbf{r}_i$, as a Taylor series expansion about the center of mass,

$$\sum_{i=1}^{N}\mathbf{F}_i(\Delta\mathbf{r}_i + \mathbf{R}) = \sum_{i=1}^{N}\left[\mathbf{F}_i(\mathbf{R}) + \frac{\partial\mathbf{F}_i}{\partial\mathbf{r}_i}\cdot\Delta\mathbf{r}_i + \mathrm{O}\left(\Delta\mathbf{r}_i^2\right)\right]. \tag{S3.25}$$

For sufficiently small displacements, the product $\frac{\partial\mathbf{F}_i}{\partial\mathbf{r}_i}\cdot\Delta\mathbf{r}_i$ can be dropped, giving

$$\mathbf{F}^{(\mathrm{total})} \sim \sum_{i=1}^{N}\mathbf{F}_i(\mathbf{R}). \tag{S3.26}$$

In the case that the (external) forces are derivable from a potential function, Eq. S3.26 holds when the second derivatives of the potential are small.

If we assume the particles are sufficiently proximate at time t such that $\boldsymbol{\nabla}_i U(\mathbf{r}(t))$ can be treated as approximately constant across the space occupied by the N-cluster, then coarse-graining according to the above prescription will approximately preserve net diffusion to first order in the displacements, $\Delta\mathbf{r}_i$, of the particles. Under this condition, the total force on the cluster can be approximated by the external force on the CG particle. For the form of the potential in Eq. (S3.7), we expect diffusive behavior orthogonal to the z-coordinate for an N-cluster to deviate from that of the CG particle since the potential is nonlinear along those directions. However, as our transition rate is determined by the constant-gradient potential in the z-direction in Eq. (S3.6), the transition rate will be statistically identical for CG particles if the same identical initial conditions as the N-clusters are used. This is the key result that assures the average transition rate is unchanging with respect to N.

Double-barrel simulation parameters

We emphasize that the double-barrel system was designed to produce noisy, non-trivial trajectories to test PSA and the path metrics. The parameter values used for the double-barrel simulations,

while guided by realistic physical systems, were determined in part by our coarse-graining constraints and practical considerations (i.e., numerical stability of the integrator, simulation time, number of simulation steps, etc.).

Table 1 summarizes the parameters used for single- and eight-particle simulations. The potential was constructed to produce potential energy changes on the order of (or less than) 10 $\mathrm{kJ \cdot mol^{-1} Å^{-1}}$ with a time step then chosen as large as allowed by the numerical stability of the integrator. The friction coefficient, γ, was heuristically chosen to reflect typical values using water as a solvent, which can range from 0.1–100 ps to depending on the solute of interest [5–8]. Spring constants, which approximate bond strength, are usually on the order of 0.1–10 $\mathrm{kJ \cdot mol^{-1} \cdot Å^{-2}}$ [9–11] and were set to within an order of magnitude of values used in the literature.

Table 1. Parameters for double-barrel simulations of one- and eight-particle clusters.

	Potential landscape parameters					Dynamical parameters		
N	A^* (kJ/mol/nm^2)	B^* (kJ/mol/nm^4)	C (nm)	F^* (kJ/mol/nm)	k_s (kJ/mol/nm^2)	$\gamma^\dagger$ (ps^{-1})	m^* (Da)	$\Delta t^\ddagger$ (fs)
1	30.0	12.18	0.8	7.5	—	50.0	10.0	0.5-2.5
8	3.75	1.523	0.8	0.9375	41.84	50.0	1.25	0.5-2.5

Simulations were performed in 50 K steps from 0 K to 500 K, and also at 600 K. Values for $N = 8$ are either unchanged from the $N = 1$ case or scaled down by a factor of eight to produce (zero-temperature) center-of-mass dynamics corresponding to single-particle dynamics. A controls x-direction confinement, B and C control the barrel shape, with $y = \pm C$ being the barrel minima in nanometers, and F is the ramp steepness. The collision frequency, γ, was the same for all systems, but particle masses (and friction coefficient, $\zeta = m\gamma$) were scaled to keep total cluster mass constant.
*Value of parameter was scaled inversely proportionally to N.
†Constant collision frequency; friction coefficient, $\zeta = m\gamma$, scales proportionally to the particle mass, m, and particle number, N.
‡Time steps were chosen to mitigate simulation time while maintaining integrator stability for a given temperature. Values for $N = 8$ are either unchanged from the $N = 1$ case or scaled down by a factor of eight to produce (zero-temperature) center-of-mass dynamics corresponding to the dynamics of one-particle systems. Simulations were performed in 50 K steps from 0 K to 500 K, and also at 600 K. Time steps were chosen heuristically at each temperature to balance simulation length and stability of the integrator.

References

[1] Abraham MJ, van der Spoel D, Lindahl E, Hess B, and the GROMACS development team. Gromacs User Manual version 5.0.4; 2014.

[2] Batchelor GK. An Introduction to Fluid Dynamics. Cambridge Mathematical Library. Cambridge University Press; 2000.

[3] Einstein A. Fürth D, Cowper AD, editors. 956, Investigations on the Theory of Brownian Movement. Dover; 1926.

[4] Peskir G. On the Diffusion Coefficient: The Einstein Relation and Beyond. Stoch Models. 2003;19(3):383–405.

[5] Cerutti DS, Duke R, Freddolino PL, Fan H, Lybrand TP. Vulnerability in Popular Molecular Dynamics Packages Concerning Langevin and Andersen Dynamics. J Chem Theory Comput. 2008 14 Oct;4(10):1669–1680.

[6] Loncharich RJ, Brooks BR, Pastor RW. Langevin dynamics of peptides: the frictional dependence of isomerization rates of N-acetylalanyl-N'-methylamide. Biopolymers. 1992 May;32(5):523–535.

[7] Widmalmt G, Pastor RW. Equilibrium and Dynamics of Ethylene Glycol in Water. J Chem Soc Faraday Trans. 1992;88(13):1747–1754.

[8] Pastort RW, Karplus M. Parametrizatlon of the Friction Constant for Stochastic. J Phys Chem. 1988;92:2636–2641.

[9] Tirion MM. Large Amplitude Elastic Motions in Proteins from a Single-Parameter, Atomic Analysis. Phys Rev Lett. 1996 26 Aug;77(9):1905–1908.

[10] Romo TD, Grossfield A. Validating and improving elastic network models with molecular dynamics simulations. Proteins. 2011 Jan;79(1):23–34.

[11] Leioatts N, Romo TD, Grossfield A. Elastic Network Models are Robust to Variations in Formalism. J Chem Theory Comput. 2012 10 Jul;8(7):2424–2434.
