# Supplementary material for: Path Similarity Analysis: A Method for Quantifying Macromolecular Pathways
Source: PLoS Comput Biol. 2015 Oct 21;11(10):e1004568. doi: 10.1371/journal.pcbi.1004568 (PMC4619321; doi:10.1371/journal.pcbi.1004568)
Supplement: S5 Text — Here we provide a short review—for reader convenience—of the transition path generating algorithms used in the comparison of sampling methods. We summarize the key aspects of each of the physical models and path generating algorithms to help lay the groundwork for connecting algorithmic/model differences to differences between the respective transition paths that were produced. (PDF) [file pcbi.1004568.s005.pdf]

Supporting Information

S5 Text. **Expanded overview of the transition path generating algorithms used in this study.**

Here we provide a short review—for reader convenience—of the transition path generating algorithms used in the comparison of sampling methods. We summarize the key aspects of each of the physical models and path generating algorithms to help lay the groundwork for connecting algorithmic/model differences to differences between the respective transition paths that were produced.

Overview

The sampling methods used to generate paths were selected primarily among those available on publicly accessible servers. DIMS-MD [1, 2] and FRODA [3] trajectories were generated in-house to produce large AdK and DT path ensembles, which was not feasible for the other methods through their web server interfaces. Table 1 summarizes the primary references and web server locations for each method. We urge the reader interested in greater detail to refer to the original articles published on each method for complete descriptions and explanations.

DIMS, MDdMD, and GOdMD are all non-deterministic dynamical algorithms that implement importance sampling in the form of a soft-ratcheting Metropolis condition, although MDdMD and GOdMD are hybrid methods that also incorporate additional biasing based on normal mode information derived from coarse-grained potentials. The FRODA algorithm is dynamical and deterministic, but can be made stochastic by turning on a random motion setting that performs a random displacement/rotation of each rigid subunit prior to each step. The five ENM-based methods (counting MENM-SD and MENM-SP separately) are all based on double-well anisotropic network models, but use different approaches to generating a single (mixed) potential, and how they generate a minimum energy path. MAP is unique in that it solves for the path that minimizes the Onsager-Machlup action subject to Brownian dynamics boundary conditions, whereas the other ENMs find steepest descent or saddle point paths.

Transition path methods details

DIMS MD [1, 2] employs an informational criterion to bias a transition toward a target structure. Here we use DIMS MD with the soft ratcheting algorithm together with Langevin dynamics and the Analytical Continuum Electrostatics (ACE) model to generate stochastic MD steps in an implicit solvent bath, as described previously [12]. A 1 fs time step was used with a collision frequency (damping coefficient) of 25 $p^{-1}s$. Steps toward the target (measured using heavy-atom rmsd-to-target as the progress variable) are always accepted, while steps increasingly farther away from the target are accepted with decreasing probability according to a Boltzmann distribution of the square of

Table 1. Primary references and public web servers (if available) for path the path generation methods that were employed in the study.

Name	Reference	Web server*	Adjusted parameter[†]
DIMS	Perilla et al. [2]	–	re-run
FRODA	Farrell et al. [3]	`pathways.asu.edu`	re-run
MDdMD	Sfriso et al. [4]	`mmb.irbbarcelona.org/MDdMD/`	re-run
GOdMD	Sfriso et al. [5]	`mmb.irbbarcelona.org/GOdMD/`	relax: 20,50,80 ps
rTMD	Ferrara et al. [6]	–	k, pull speed, re-run
ANMP	Das et al. [7]	`anmpathway.lcrc.anl.gov/anmpathway.cgi/`	cutoff: 12,15,18 Å
MAP	Franklin et al. [8]	`lorentz.dynstr.pasteur.fr/joel/traj_newpath_submission.php`	cutoff: 7,10,13 Å
MENM	Zheng et al. [9]	`enm.lobos.nih.gov/start_path.html`	cutoff: 8,10,12 Å
iENM	Tekpinar and Zheng [10]	`enm.lobos.nih.gov/start_ienm.html`	cutoff: 8,10,12 Å
Morph	Krebs and Gerstein [11]	`molmovdb.org/cgi-bin/submit.cgi`	fit/min, fit, none
LinInt	–	–	–

The methods used to generate transition paths are given with their primary references and corresponding URL for submitting jobs. Three different transitions were generated per method (except LinInt) by adjusting a single parameter to three different values; Morph transitions were produced by varying input structure superimposition and energy minimzation settings.

*Web server: URL of job submission page

[†]Adjusted parameter: re-run, initial conditions automatically randomized; relaxation, relaxation window in picoseconds; k, spring constant; pull speed, speed of equilibrium position of moving restraint; cutoff, spring cutoff distance in Ångström; fit, superimposition of input structures; min, energy minimization (adiabatic mapping).

the order parameter. Soft ratcheting has emerged as a robust approach to ensure overall progress toward the target structure while retaining the capability to back out of energetic dead ends [2, 12].

FRODA [3] advances proteins toward a target structure by incrementally reducing the rmsd while enforcing steric constraints. Random paths are produced by introducing random perturbations to the orientation of one side chain per simulation step. Contact constraints shared by the initial and final conformations were preserved throughout the simulations, while non-common contacts were allowed to be broken and/or formed. Contacts were defined as atom pairs falling within an 8 Å cutoff radius.

Both **MDdMD** [4] and **GOdMD** [5] employ deterministic discrete MD to quickly sample large regions of configuration space; for each simulation a sub-sequence of configurations is dynamically constructed by selecting conformer snapshots on the fly according to a Metropolis-like Monte Carlo (MC) procedure combined with informational criteria. The sub-sequences represent physically plausible transition paths. In both approaches, progress is driven toward a final state using an soft ratcheting-like algorithm. MDdMD further selects pre-accepted snapshots based on the degree of eigenvector overlap with the essential transition vector. The essential transition vector is computed (via NMA of a Gō-like potential) using primarily the initial state. GOdMD differs in that it employs an ENM-metadynamics method to bias proteins away from the initial energetic well.

The MDdMD method employs simplified or multi-step square potentials for bonded interactions at the atomic level. Solvent, van der Waals, and electrostatic forces are modeled using two-step square wells for attractive interactions and soft barriers for repulsion. GOdMD is based on a C_α representation. Square wells define the physical chemistry while a multi-well Gō-like potential describes non-bonded interactions. For both methods the initial velocities are randomized so that separate runs generate distinct trajectories. The three GOdMD trajectories were produced, however, by varying the relaxation window: 20 ps, 50 ps and 100 ps.

The **rTMD** [6] (restrained TMD) method resembles the original TMD algorithm which was based on a time-dependent holonomic constraint that moves linearly toward a target structure; in the rTMD variation, a moving harmonic restraint is used instead. As with DIMS, the rTMD

method requires a progress variable in the form of an atom selection for biasing. rTMD simulations were set up to mimic DIMS simulations as closely as possible: the heavy-atom rmsd-to-target progress variable was used for biasing and rmsd fitting; we used Langevin dynamics with an identical time step and collision frequency, and the Generalized Born implicit solvent as implemented in the NAMD simulation code [13] with a matching time step, collision frequency. During each time step, the equilibrium position of the harmonic restraint is moved a fixed amount by decreasing the rmsd to the target conformation. We generated six transitions in total, three for each of the following settings (using velocities randomly sampled from the Maxwell-Boltzmann distribution): three *fast*-pulling simulations with $k = 4.184 \times 10^5$ kJ/mol/Å and ~ 1 Å/ps pulling speed; three *slow*-pulling simulations with $k = 4.184 \times 10^3$ kJ/mol/Å and ~ 0.01 Å/ps pulling speed.

Using the C_α positions of an initial and final structure, the **CG-EN** models examined here construct two energetic potentials around the respective states. The separate potentials are then combined (e.g. through a heuristic mixing rule) to form a double-well potential that models the energy landscape of the configuration space spanning the two states. A path is generated from the double well potential using, for instance, a minimum energy path (MEP) approach. The **ANMP** [7] algorithm constructs two anisotropic network models (ANM) about their respective end states, iteratively searches for transition state defined as the minimum energy structure lying on the cusp hypersurface of a combined two-state potential, and performs two steepest descent (SD) minimizations from the transition state to generate a sequence of conformers that represent a conformational path. Three transitions were generated using the following spring cutoff distances: 12 Å, 15 Å and 18 Å. Similarly, **MAP** [8] generates a separate ANM potential around each end state. The MAP transition is generated by first minimizing the Onsager-Machlup action assuming overdamped Langevin dynamics, then the resulting deterministic equations are solved analytically for positions and velocities using appropriate boundary conditions. Spring cutoff distances were set to 7 Å, 10 Å and 13 Å.

The AD-ENM Web Server [9, 10] offers two approaches to generating ENM transitions. Like the ANMP and MAP models, both approaches are based on ANM representations of the initial and final protein configurations. The **MENM** of the PATH-ENM server generates a double-well potential from a predefined mixing function. The energetic minima and saddle points (SPs) are computed and transitions are generated in one of two ways: (1) The steepest descent (SD) paths about each SP; (2) The path tracing the minima and SPs. The **iENM** server exploits the independence of the equation for the minima and SPs on the detailed form of the mixing function. An iterative procedure is used to solve for the universal minimum-energy path (MEP) through the SPs of an arbitrary double-well potential that includes a predefined steric collision energy. Three distinct transitions for each method were produced using an 8 Å, 10 Å and 12 Å spring cutoff distance.

The **Morph** [11] servers generate transitions using adiabatic mapping, which combines simple linear interpolation (between two structures) and CHARMM-based energy minimization to reduce severe steric clashing. Two transitions were generated using stepwise energy minimization: one transition used the 'superimpose before morphing' option, while the other relied on our CORE alignment procedure (described in S4 Text), allowing us to compare possible path differences arising from structural pre-alignment. A third transition without both energy minimization and superimposition was also performed.

We also generated a zeroth-order transition path using naive linear interpolation (**LinInt**). The path is defined by a sequence of evenly spaced conformers lying along the line formed by the separation vector between the C_α representations of the initial and final states. We used LinInt as a convenient reference path for the comparison of the higher-order methods.

References

[1] Woolf TB. Path corrected functionals of stochastic trajectories: towards relative free energy and reaction coordinate calculations. Chem Phys Lett. 1998 19 Jun;289(5–6):433–441.

[2] Perilla JR, Beckstein O, Denning EJ, Woolf TB. Computing ensembles of transitions from stable states: Dynamic importance sampling. J Comput Chem. 2011 30 Jan;32(2):196–209.

[3] Farrell DW, Speranskiy K, Thorpe MF. Generating stereochemically acceptable protein pathways. Proteins. 2010 1 Nov;78(14):2908–2921.

[4] Sfriso P, Emperador A, Orellana L, Hospital A, Gelpí JL, Orozco M. Finding Conformational Transition Pathways from Discrete Molecular Dynamics Simulations. J Chem Theory Comput. 2012;8(11):4707–4718.

[5] Sfriso P, Hospital A, Emperador A, Orozco M. Exploration of conformational transition pathways from coarse-grained simulations. Bioinformatics. 2013 15 Aug;29(16):1980–1986.

[6] Ferrara P, Apostolakis J, Caflisch A. Computer simulations of protein folding by targeted molecular dynamics. Proteins. 2000 15 May;39(3):252–260.

[7] Das A, Gur M, Cheng MH, Jo S, Bahar I, Roux B. Exploring the conformational transitions of biomolecular systems using a simple two-state anisotropic network model. PLoS Comput Biol. 2014 Apr;10(4):e1003521.

[8] Franklin J, Koehl P, Doniach S, Delarue M. MinActionPath: maximum likelihood trajectory for large-scale structural transitions in a coarse-grained locally harmonic energy landscape. Nucleic Acids Res. 2007 Jul;35(Web Server issue):W477–82.

[9] Zheng W, Brooks BR, Hummer G. Protein conformational transitions explored by mixed elastic network models. Proteins. 2007 1 Oct;69(1):43–57.

[10] Tekpinar M, Zheng W. Predicting order of conformational changes during protein conformational transitions using an interpolated elastic network model. Proteins. 2010 15 Aug;78(11):2469–2481.

[11] Krebs WG, Gerstein M. The morph server: a standardized system for analyzing and visualizing macromolecular motions in a database framework. Nucleic Acids Res. 2000 15 Apr;28(8):1665–1675.

[12] Beckstein O, Denning EJ, Perilla JR, Woolf TB. Zipping and Unzipping of Adenylate Kinase: Atomistic Insights into the Ensemble of Open $\leftrightarrow$ Closed Transitions. J Mol Biol. 2009 Nov;394(1):160–176.

[13] Phillips JC, Braun R, Wang W, Gumbart J, Tajkhorshid E, Villa E, et al. Scalable molecular dynamics with NAMD. J Comput Chem. 2005 Dec;26(16):1781–1802.
