# Supplementary material for: Path Similarity Analysis: A Method for Quantifying Macromolecular Pathways
Source: PLoS Comput Biol. 2015 Oct 21;11(10):e1004568. doi: 10.1371/journal.pcbi.1004568 (PMC4619321; doi:10.1371/journal.pcbi.1004568)
Supplement: S6 Text — This text summarizes the considerations involved in structurally aligning conformer snapshots prior to running path similarity analysis on a set of transition paths. We provide specific details and motivations as to the alignment procedures used for AdK and DT trajectories. (PDF) [file pcbi.1004568.s006.pdf]

Supporting Information

S6 Text. Details of structural alignment procedures for protein alignment prior to path similarity analysis.

This text summarizes the considerations involved in structurally aligning conformer snapshots prior to running path similarity analysis on a set of transition paths. We provide specific details and motivations as to the alignment procedures used for AdK and DT trajectories.

Structural alignment considerations

In general, a simulation snapshot will be a (protein) structure having an arbitrary orientation and center-of-mass translation. Since the rmsd between two conformations of the same structure will depend on their relative orientation and separation, it is necessary to employ an alignment procedure to ensure a unique rmsd will be computed for the pair. The best-fit rmsd between a structure pair is computed by first aligning the centers of mass and finding the rotation (matrix) that minimizes the rmsd. Though this approach is common, we discuss several reasons why the best-fit rmsd may not be the best choice of point metric for PSA.

Best-fit rmsd as a point metric

To perform PSA of a collection of transition paths, we must compute path similarities for all unique pairs of paths. For each pair of paths P and Q, there are p and q snapshots, respectively. Using the rmsd as the point metric, the Hausdorff and Fréchet distance between P and Q both require pq rmsd calculations. If the best-fit rmsd is to be used, then pq optimizations must be performed, corresponding to pairwise alignment of all unique pairs of structures between P and Q.

While it may be thought that computing the best-fit rmsd be computed on a pairwise basis, there are two drawbacks to this approach: The first issue has to do with computational cost, as rmsd optimization, though relatively fast using the Quaternion Characteristic Polynomial (QCP) algorithm [1, 2], can quickly become computationally expensive. Given N transition paths, each having s snapshots, to be compared with PSA, there are $N(N-1)/2$ unique path comparisons. For each comparison (of two paths), there are s^2 unique pairs of conformers, one conformer each, between them. The pairwise best-fit rmsd approach would then necessitate $N(N-1)s^2/2$ optimizations: the cost grows proportionally to the product of square of the number of paths and the product of the numbers of time steps in each pair of paths. Comparing an ensemble of hundreds of trajectories, each composed of hundreds of conformer snapshots, can easily demand upwards of a billion best-fit rmsd optimizations. The second problem is that the pairwise-minimal rmsd will not generally preserve the triangle inequality and thus will not behave as a proper metric on configuration space [3]. Intuitively, it can be seen that, since a triplet of trajectories will have three unique rotational alignments between each of the three combinations of unique trajectory pairs, and since each rotation depends only

on the trajectories in the pair, pairwise-minimal rmsd measurements need not obey the transitive property and can thus violate the triangle inequality.

The Hausdorff and Fréchet metrics are only proper metrics provided that they are defined in terms of a (proper) point metric. Thus, Hausdorff and Fréchet calculations will not preserve metric properties if the best-fit rmsd is used. One may argue that the metric requirement may be relaxed for the path metrics without affecting the quality of results. It is also possible that there are benefits to using the best-fit rmsd to maximize consistency with its use in the literature. We were nevertheless able to obtain sensible and consistent results using heuristic alignment schemes (discussed next) that preserve all the properties of a metric. Although the relationship of structural similarity measures and PSA is interesting and likely worth further examination, it is out of the scope of this paper.

Alignment procedures used in study

To mitigate computational costs, our current implementation of PSA utilizes a pre-alignment procedure: for each path, the rotation matrix R_i that minimizes the rmsd between frame i and a single reference structure is computed, then used to rotate frame i. Thus, the alignment of all conformer snapshots in a path P to a pre-defined reference structure scales linearly with respect to the number of snapshots, p, in a path, whereas pairwise alignment scales quadratically. Given a path ensemble, a single reference structure common to the entire ensemble is used as the basis for aligning each conformer in each path. Although a poor choice of reference structure may be suboptimal, we found that our procedure produces sensible results at much reduced computational cost and complexity. Furthermore, the use of a single reference structure for the structural superposition preserves the triangle inequality for the point metric and thus imbues δ_H and δ_F with the qualities of a proper metric for paths.

AdK trajectory alignment

In the special case of AdK, since the conformational motion is known to be primarily confined to the NMP and AMP domains and the hinges connecting them to the CORE domain, we chose to align the CORE domain of each intermediate conformation to the average of the aligned CORE C_α coordinates (using the best-fit rmsd) of the 1AKE:A and 4AKE:A structures with the center of mass of the averaged CORE at the origin. We chose to use the average CORE C_α coordinates as a putative reference structure to reduce the alignment bias of intermediate snapshots residing closer to one of the boundary conformations than the other. The alignment procedure, in the context of the comparison of transition path methods, is demonstrated in the example Python script `psa_full.py` in the PSA tutorial, which is available as open source at `github.com/Becksteinlab/PSAnalysisTutorial` under the GNU General Public License 3.

To align a given AdK conformer snapshot, it was translated so that the center of mass of its C_α CORE atoms coincided with the origin. All of the atoms of the conformer were then rigidly rotated according to the rotation matrix that generated the best-fit rmsd between the conformer's C_α CORE and the (C_α-CORE) reference structure, using the QCP algorithm [1,2] implemented in MDAnalysis [4]. The entire structure of each snapshot in each path, for all paths in a transition path comparison, was translated and rotated so as to align each C_α-CORE region to the reference C_α-CORE coordinates. Path metric calculations were then performed on the set of aligned paths, where for each structural comparison, the C_α rmsd was directly computed without any further rotation/alignment. In the hypothetical case where the C_α CORE domains of two intermediate conformers have coordinates identical to the reference coordinates, the C_α rmsd between the (entire) intermediate structures will be solely due to C_α deviations in the LID and NMP domains. Therefore, this alignment procedure produces rmsds reflecting residue displacements in the mobile LID and NMP domains, and not the CORE domains. In the case of the path-sampling methods comparison,

structural rmsd measurements were made once CORE domains were aligned. The comparison between DIMS and FRODA used the same alignment protocol outlined above with the added step of translating the center of mass of all conformer snapshots to the origin (instead of just the CORE). This was done in part to lower the rmsd and path distance measurements further to see if the effectiveness of the Hausdorff pairs comparison would be reduced; our results suggest that Hausdorff pairs analysis is still viable.

DT trajectory alignment

We found that a satisfactory alignment procedure for DT transition paths was achieved by using the average C_α coordinates of the full 1MDT:A and 1DDT:A structures to generate a reference structure. The DT path ensembles were otherwise aligned in an identical manner to the AdK ensembles: each conformer snapshot for each path was aligned to the reference prior to calculating path similarities; after aligning each path, the rmsd between conformers in different paths without any further rotations/alignments was used as the point metric.

Although DT is conceptualized as having a mobile Translocation (T) domain that moves relative the Catalytic (C) and Receptor-binding (R) domains, it was not amenable to the alignment procedure we used for AdK (see Fig. 2 in the main text). If we were to carry out an analogous procedure, we must align the C_α atoms corresponding to the C and R domains in the 1MDT:A and 1DDT:A end structures. The average coordinates of the 1MDT:A C/R C_α atoms and 1DDT:A C/R C_α atoms would then be then be used as the reference structure to which individual conformers would be aligned. We found, however, that the procedure produced larger Hausdorff and Fréchet distances than when simply aligning the C_α atoms of entire conformers to either of the end structures.

The reason that C/R domain alignment performs poorly is likely due to a confluence of several factors. First, the C and R domains were seen to fluctuate greatly throughout DIMS and FRODA simulations and were not static to the same degree as the AdK CORE. Second, the overall shape of the C and R domains taken together is somewhat cylindrical with an approximate axis running through both, while the T domain is displaced mostly orthogonally from this axis. A structure aligned to this region may be rotated relative to another in the sense that its T domain has been swung around the cylindrical R/C domains during alignment. T domains will therefore tend to be displaced if only the R and C domains are used to produce a reference. After alignment, the rmsd becomes too sensitive to the orientation of the T domain about the R and C domain. In the case of using the entire structure to produce reference coordinates, alignment is sufficiently constrained about the R/C "axis" to prevent erroneous T domain displacements.

References

[1] Theobald DL. Rapid calculation of RMSDs using a quaternion-based characteristic polynomial. Acta Crystallogr A. 2005 Jul;61(Pt 4):478–80.

[2] Liu P, Agrafiotis DK, Theobald DL. Fast determination of the optimal rotational matrix for macromolecular superpositions. J Comput Chem. 2010 May;31(7):1561–3.

[3] Crippen GM. Series approximation of protein structure and constructing conformation space. Polymer. 2003 Jul;44(15):4373–4379.

[4] Michaud-Agrawal N, Denning EJ, Woolf TB, Beckstein O. MDAnalysis: a toolkit for the analysis of molecular dynamics simulations. J Comput Chem. 2011 30 Jul;32(10):2319–2327.
