# Supplementary figures and images for: Path Similarity Analysis: A Method for Quantifying Macromolecular Pathways
Source: PLoS Comput Biol. 2015 Oct 21;11(10):e1004568. doi: 10.1371/journal.pcbi.1004568 (PMC4619321; doi:10.1371/journal.pcbi.1004568)

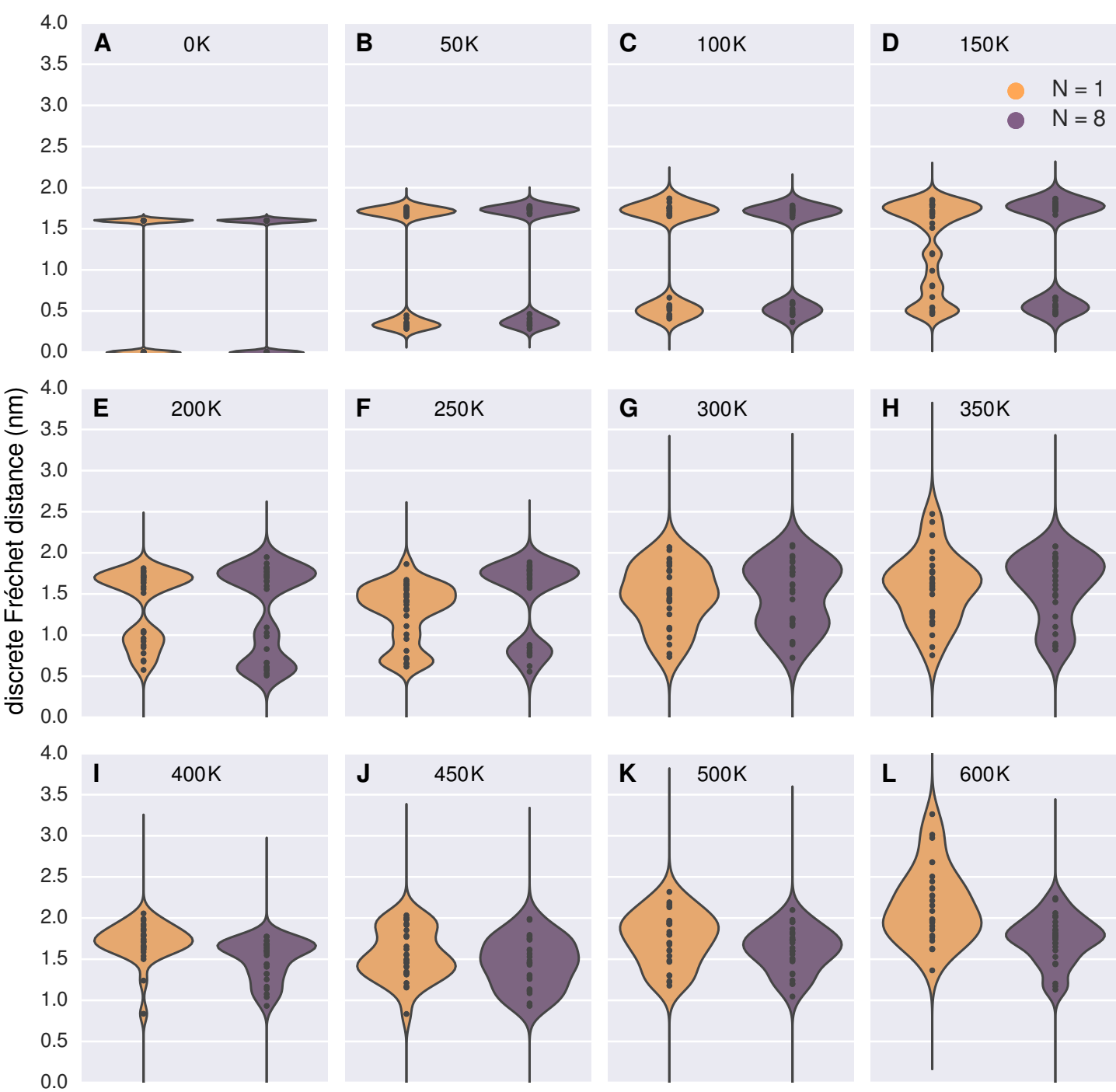

Supplement: S1 Fig — Violin plots [91] show the distributions of discrete Fréchet distances for double-barrel simulations of one particle (orange) and eight particles (purple) for temperatures ranging between 0 and 500 in 50 increments (panels A–K) and at 600 (panel L). Black points correspond to individual Fréchet distance measurements, with distance units in nm rmsd. A kernel density estimate (kde) is shown for each N, T pair to qualitatively emphasize the behaviors of the distributions across the entire temperature range; the bandwidth for each pair is explicitly set to produce two distinct distributions at low temperatures and gradually increased to generate smooth, single distributions at high temperatures. The separated distributions at low temperatures merge between 300 K to 450 K, with the eight-particle simulations merging toward higher temperatures relative to the one-particle simulations. (PDF) [file pcbi.1004568.s007.pdf]

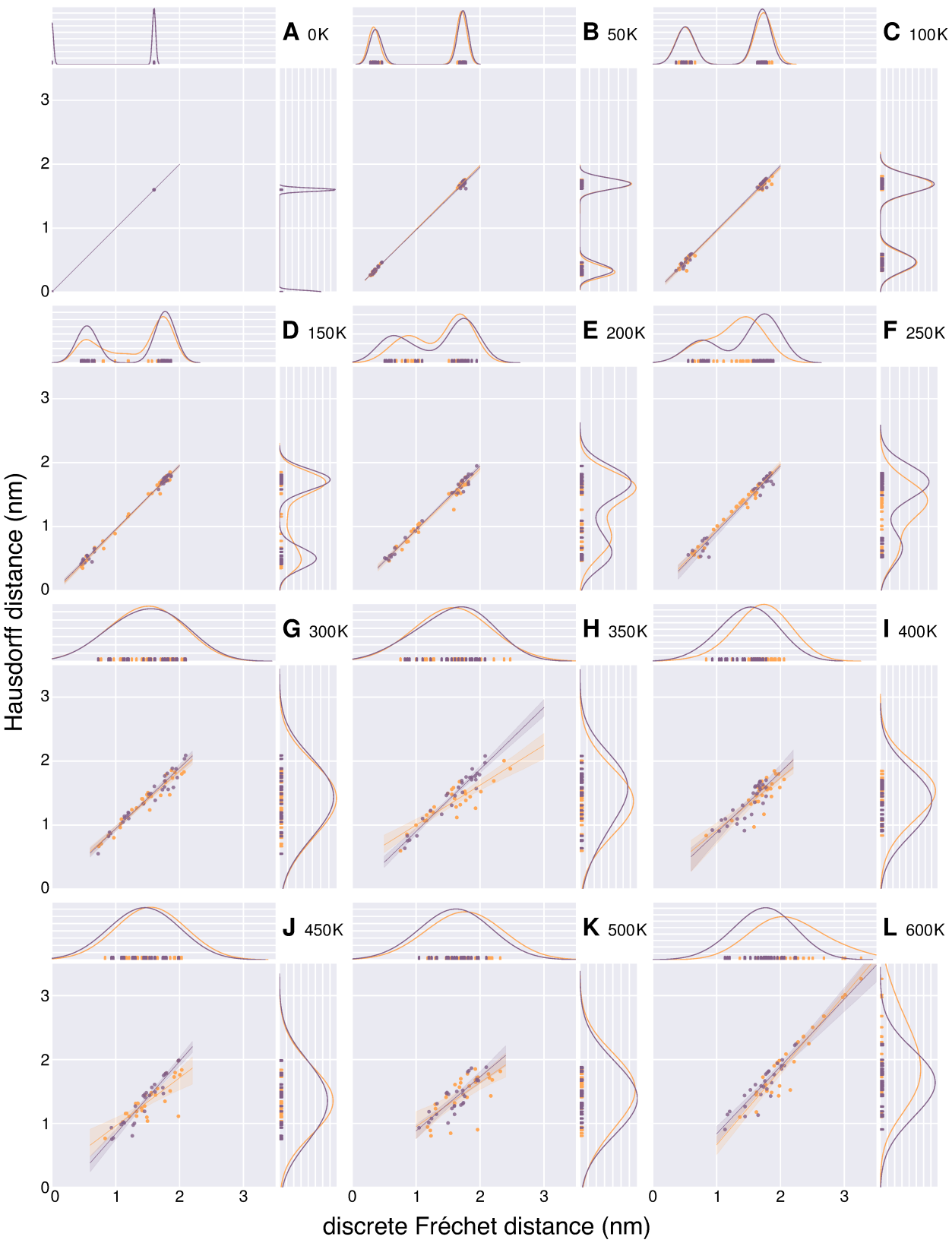

Supplement: S2 Fig — Regression analyses examining the correlation between corresponding Fréchet (horizontal axes) and Hausdorff (vertical axes) distance measurements are plotted along with the joint distributions plots for double-barrel simulations of one particle (orange points) and eight particles (purple) for temperatures ranging between 0 and 500 K in 50 K increments (panels A–K) and at 600 K (panel L). Scatter points correspond to individual Fréchet distance measurements in nm rmsd and are plotted with the line produced by linear regression. The shading about the regression lines correspond to a 95% confidence interval. Kernel density estimates (kde) are shown for each N, T pair and are computed using the same set of bandwidth constants specified in S1 Fig. The separated distributions at low temperatures merge between 300 K to 450 K, with a notable narrowing of the range of distance measurements occuring between 400 K to 450 K. (PDF) [file pcbi.1004568.s008.pdf]

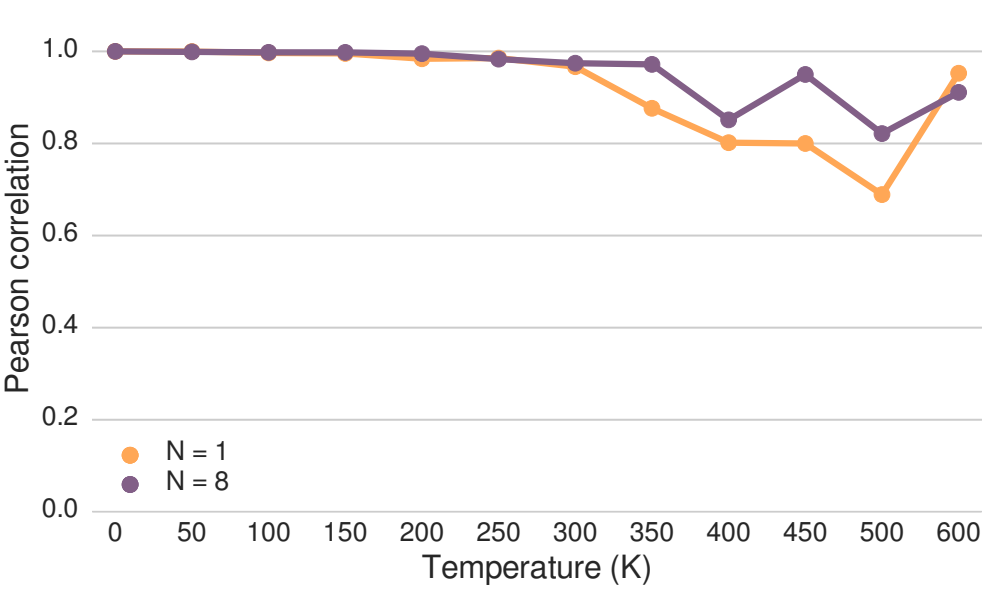

Supplement: S3 Fig — Coefficients of the Pearson correlation between Hausdorff and Fréchet distances for one- and eight-particle simulations plotted as a function of temperature. Path distances remain well correlated up to 300 K and are least correlated at 500 K, with the one-particle simulations exhibiting a substantially larger drop in correlation. At the highest temperature the central barrier becomes negligible and the simulations start to equally sample a single tube dominated by the steep repulsive walls. Therefore, the paths start becoming more similar between the N = 1 and N = 8 clusters and the correlation coefficient increases. (PDF) [file pcbi.1004568.s009.pdf]

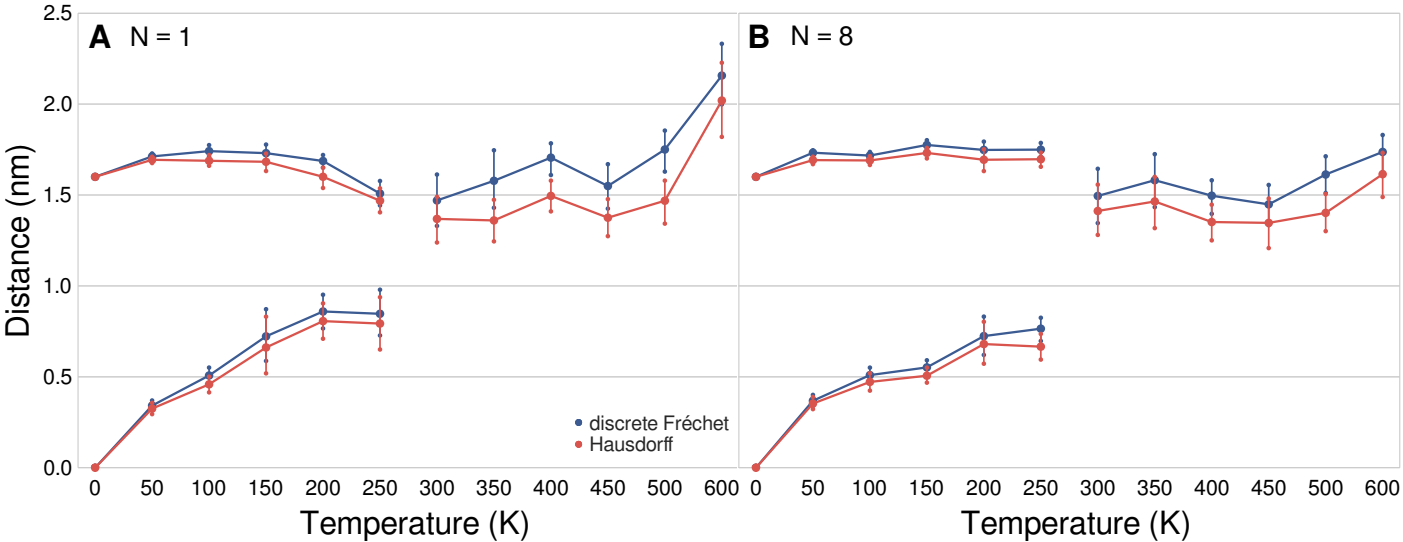

Supplement: S4 Fig — The means and standard deviations of the discrete Fréchet (blue) and Hausdorff (red) distances for double-barrel simulations of one particle (A) and eight particles (B) are shown as a function of temperature. Measurements for simulations at 250 K and below were divided into an upper and lower distribution by separating distance measurements above and below a 1.25 nm cutoff. Above the temperature cutoff, all measurements were treated as part of the same distribution. Both the Fréchet and Hausdorff metric lose the ability to distinguish between the two barrels as the paths begin to wander out of well-defined pathways when the temperature is on the order of the equivalent energy of the central barrier (2kB T at 300 K). At higher temperatures, thermal perturbations become large relative to the barrier, permitting particle clusters to explore the full width of the potential spanning both barrels so as to generate trajectories confined to a single, unified pathway. (PDF) [file pcbi.1004568.s010.pdf]

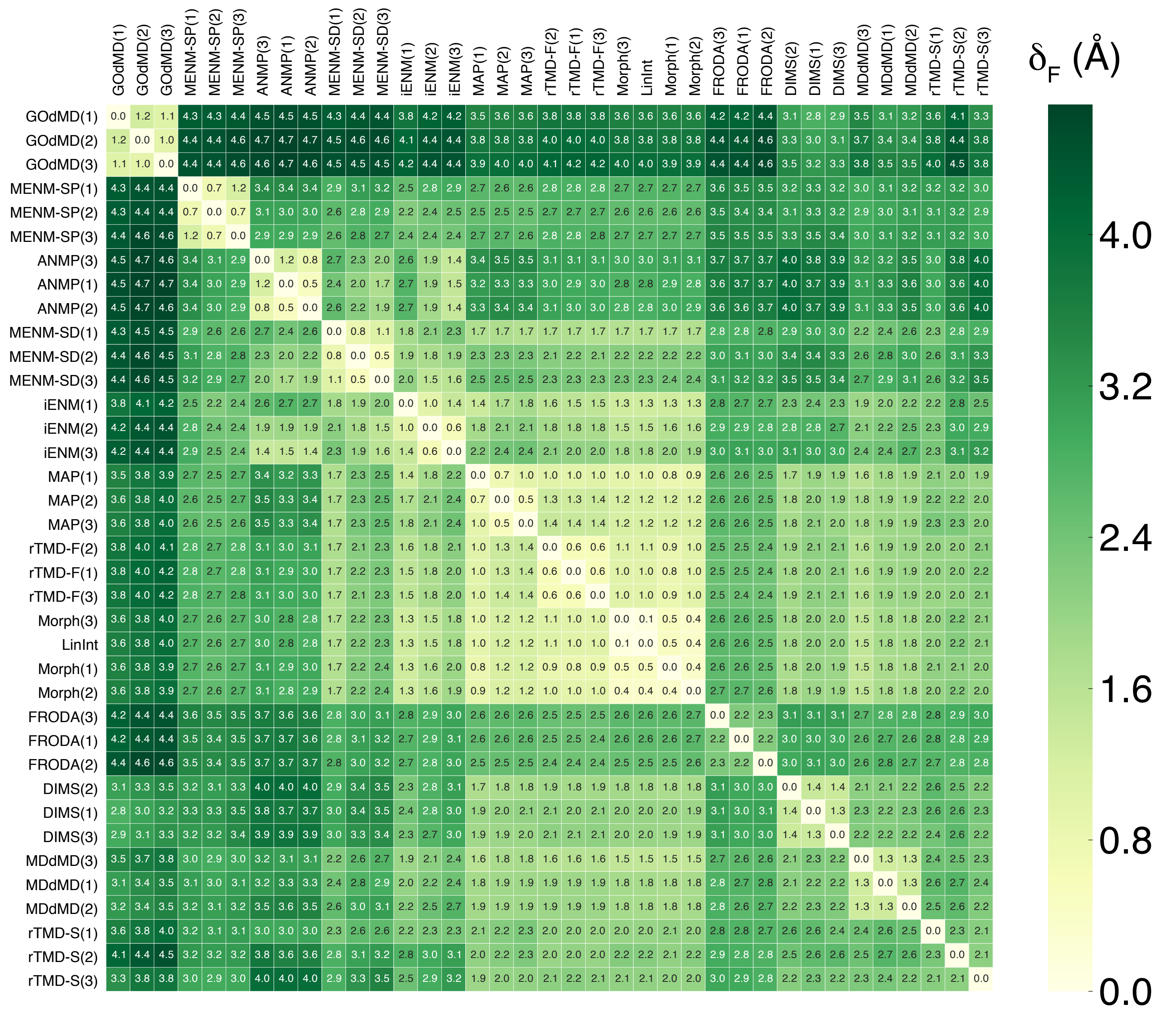

Supplement: S5 Fig — The Fréchet distance matrix from Fig 6 is shown with the numerical values of δF (rounded to one decimal) superimposed. Due to the size of the distance matrix, the high resolution image is provided as a simple means for online data exploration with the help of the zoom function of an image viewer. (PDF) [file pcbi.1004568.s011.pdf]

**A single**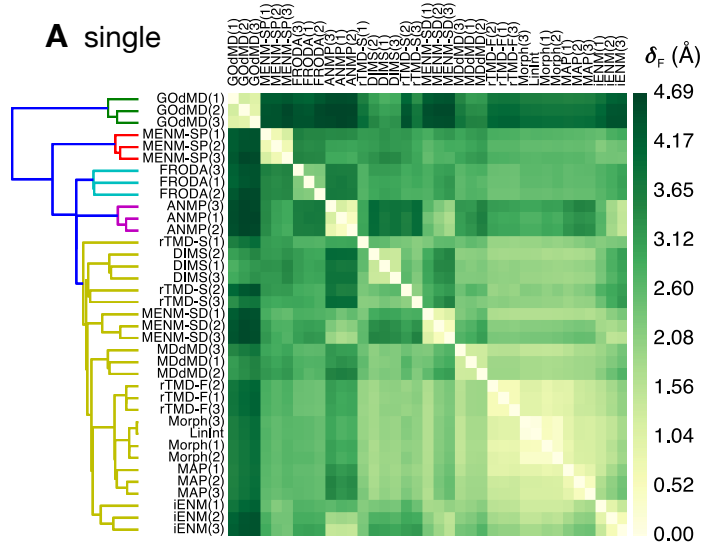**B complete**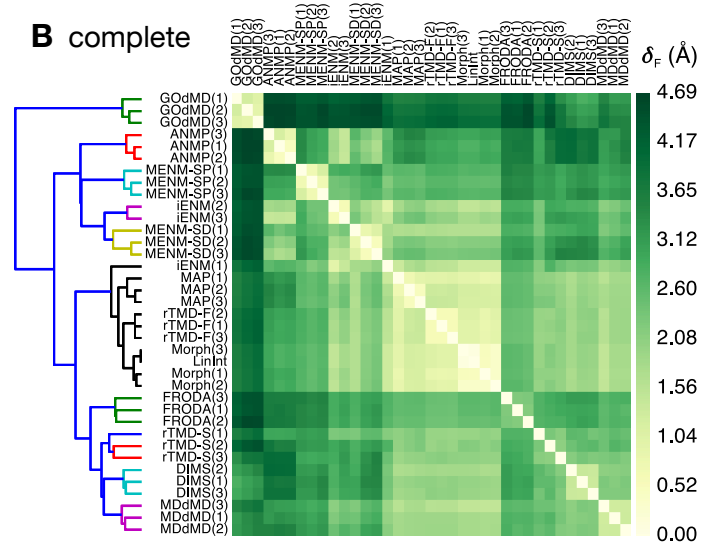**C average**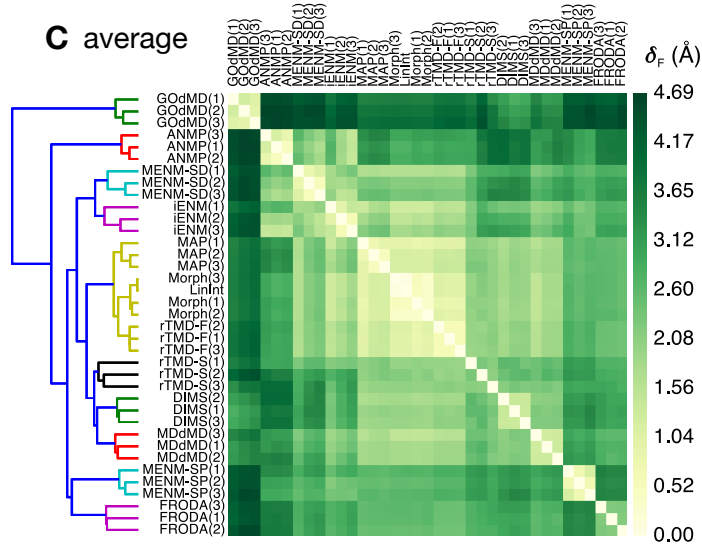**D weighted**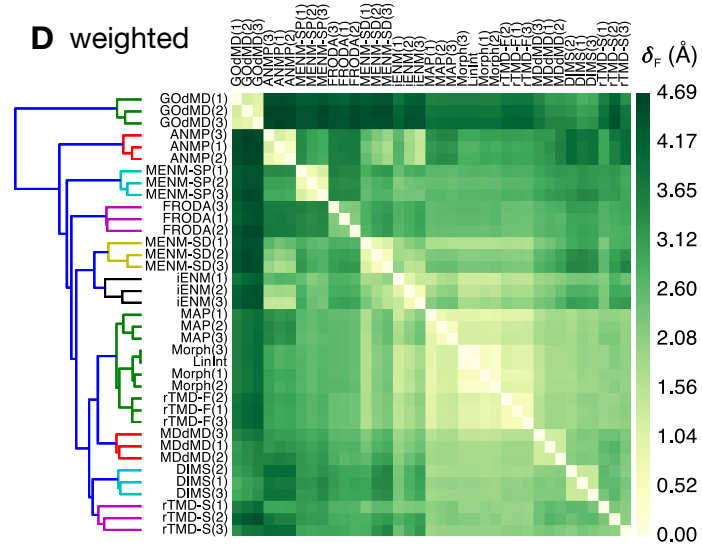

Supplement: S6 Fig — Different linkage algorithms were used to cluster the Fréchet distances produced by path-sampling methods for the AdK closed → open transition. Smaller distances (in units of Å rmsd) indicate transition paths with greater similarity. Dendrograms for each heat map correspond to the hierarchical clustering produced by the single (A), complete (B), average (C), and weighted (D) linkage algorithms, and depict a hierarchy of clusters with smaller node heights of parent clusters indicating greater similarity between child clusters. (PDF) [file pcbi.1004568.s012.pdf]

**A**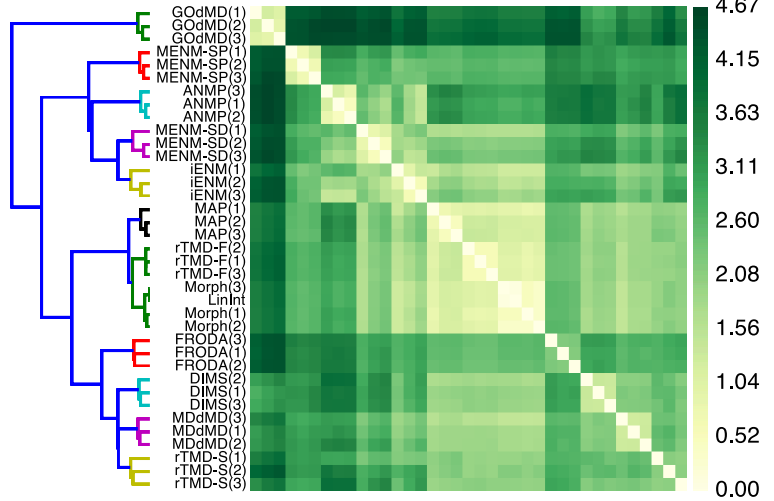**B**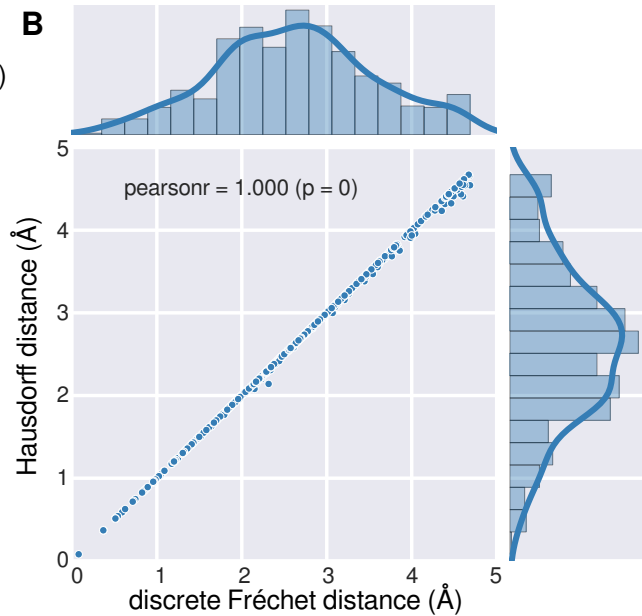

Supplement: S7 Fig — (A) Heat map for path-sampling methods for the AdK closed → open transition of Hausdorff distances produced using the Ward algorithm. Clusters are identical to the Ward clustering for Fréchet distances in Fig 6. (B) Correlation and joint distributions between discrete Fréchet versus Hausdorff distance measurements (in Å rmsd) for the AdK closed → open methods comparison. Strong linear correlation indicated by the scatter plot, with a Pearson correlation coefficient very close to unity, indicates that either metric could have been used to perform the path-sampling methods analysis with essentially identical results. A slight deviation of the scatter points below the line of unity slope is consistent with the fact that Fréchet distances are bounded from below by corresponding Hausdorff distances. (PDF) [file pcbi.1004568.s013.pdf]

discrete Fréchet distance  $\delta_F$  (Å)

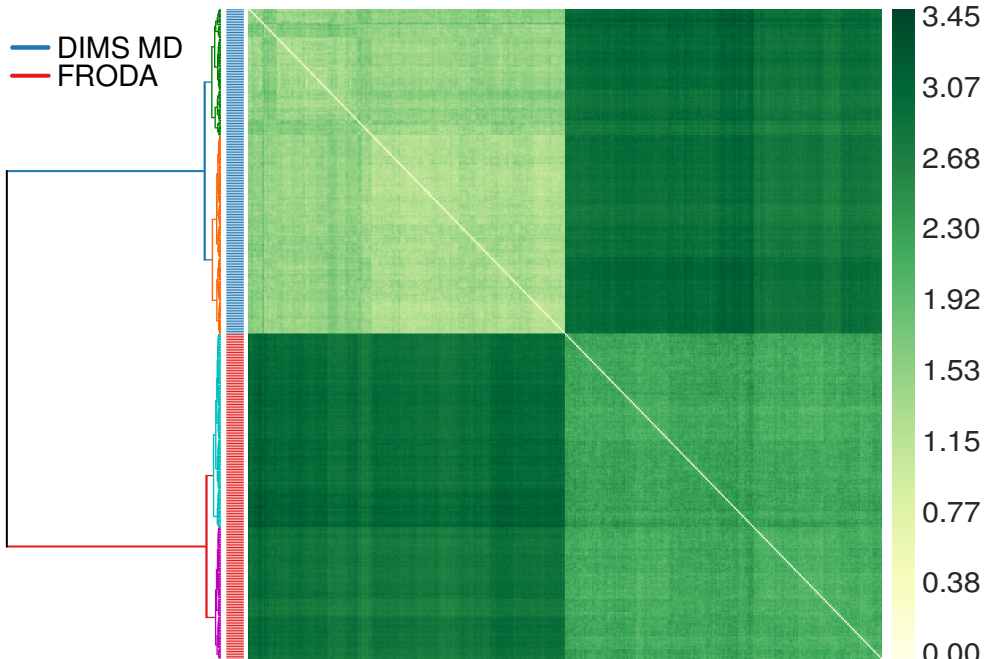

Supplement: S8 Fig — Clustered heat map comparing path ensembles of adenylate kinase (1AKE:A to 4AKE:A) transition paths produced by DIMS (red bars) and FRODA (blue bars) using the discrete Fréchet distance δF are summarized by heat map cluster analysis. Clustering was produced using the Ward algorithm in ascending distance order. (PDF) [file pcbi.1004568.s014.pdf]

discrete Fréchet distance  $\delta_F$  (Å)

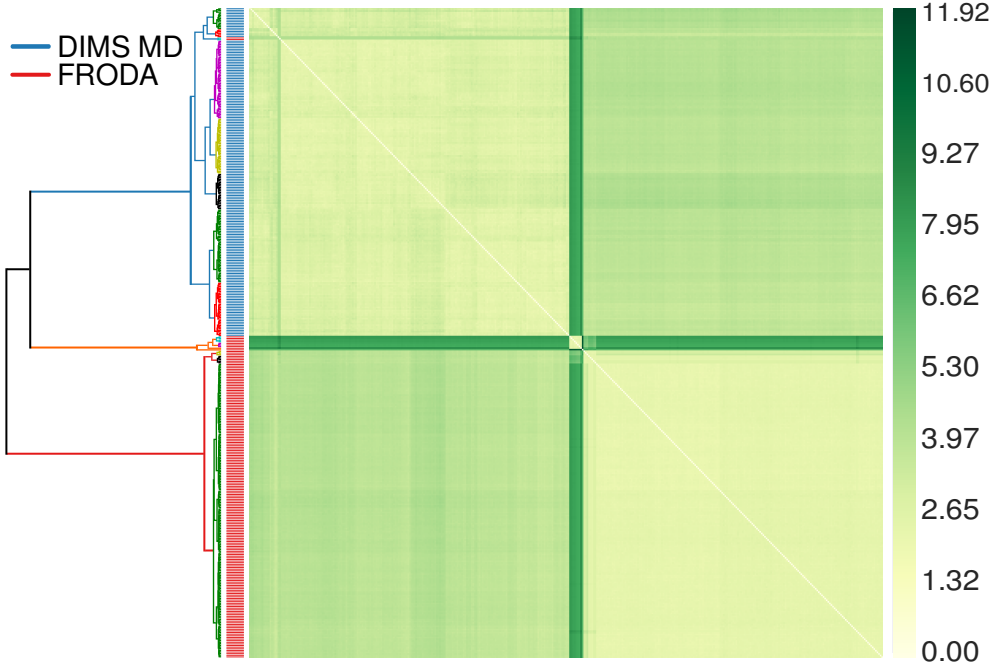

Supplement: S9 Fig — Clustered heat map comparing the raw path ensembles of diphtheria toxin (1MDT:A to 1DDT:A) transition paths produced by DIMS (red bars) and FRODA (blue bars) using the discrete Fréchet distance δF. Clustering was produced using the Ward algorithm in ascending distance order. Nine erroneous FRODA paths (orange cluster) were very distant from all other paths—all nine were removed from the ensemble and to produce the heat map dendrogram in Fig 8. (PDF) [file pcbi.1004568.s015.pdf]

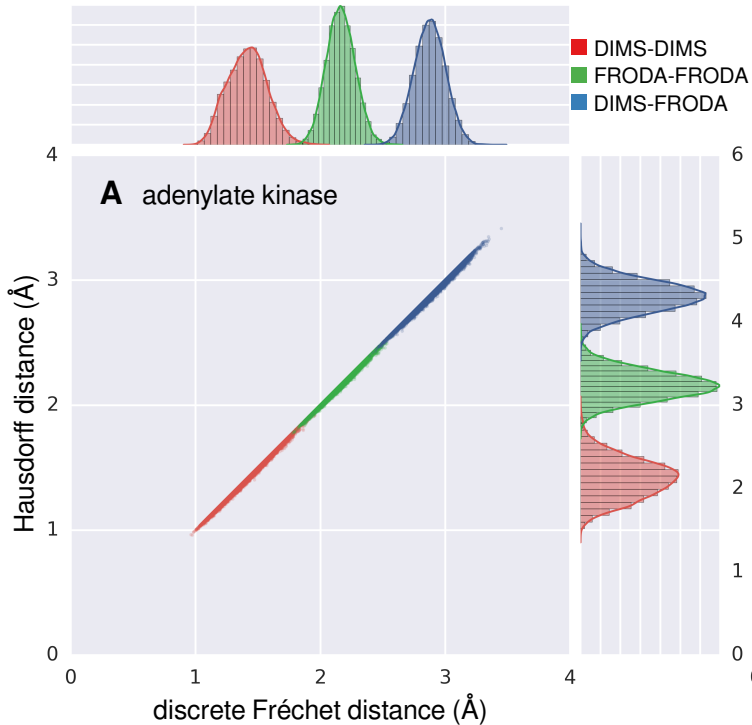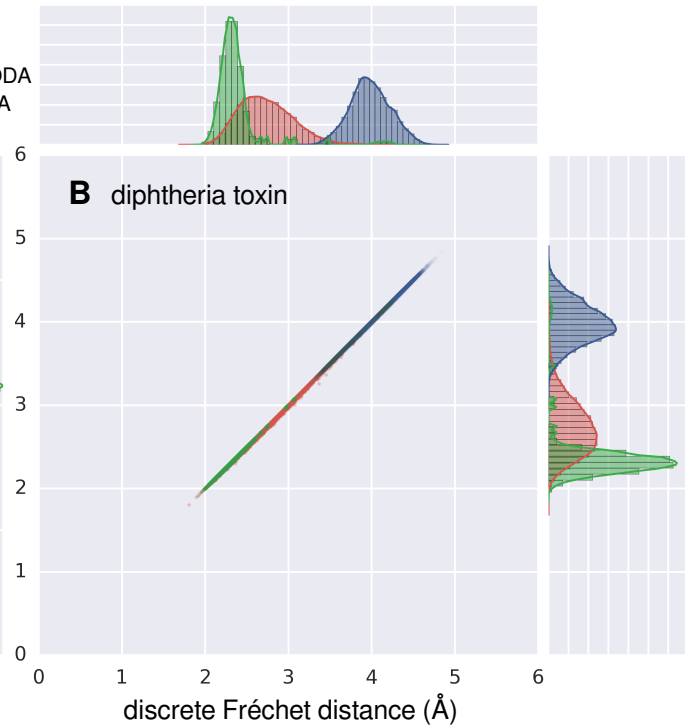

Supplement: S10 Fig — Correlations and joint distributions of discrete Fréchet versus Hausdorff distance measurements (in Å rmsd) of the AdK (A) and DT (B) ensemble analyses are shown. Measurements are divided into three separate distributions: (1) mutual distances among DIMS paths (red), (2) mutual distances among FRODA paths (green), and (3) inter-method distances measured between a DIMS and a FRODA path (blue). Both scatter plots show strong correlation between the path metrics for all the distributions, with Pearson correlation coefficients equal to unity and p-values equal to zero to two decimal places, indicating that either metric could have been used to perform the path-sampling methods analysis to obtain essentially identical results. A slight deviation of the scatter points below the line of unity slope is consistent with the fact that Fréchet distances are bounded from below by corresponding Hausdorff distances. DIMS simulations exhibited less variation than FRODA in the AdK transition, but had a larger average variation in the DT transition. In both cases, inter-method DIMS-FRODA comparisons were substantially larger than comparisons of pairs of paths produced within a single method. (PDF) [file pcbi.1004568.s016.pdf]

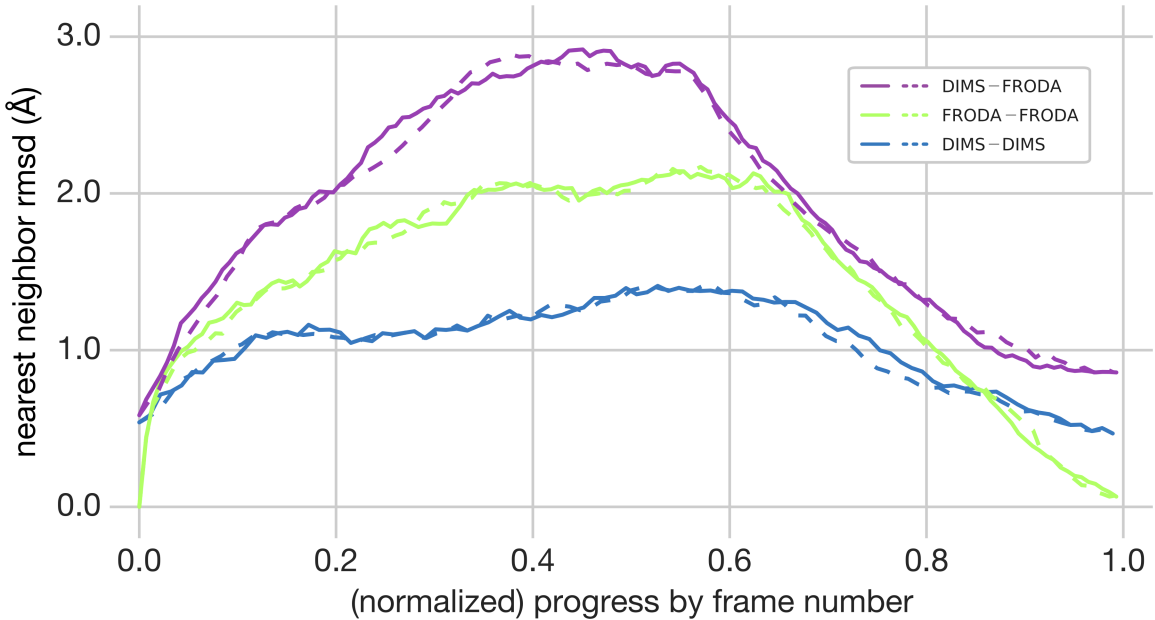

Supplement: S11 Fig — The nearest neighbor distances δh(k; Q ∣ P) (solid line, ——) and δh(k; P ∣ Q) (dashed line, ----) between pairs of paths P/Q belonging to the three median Hausdorff pairs in the AdK ensemble comparison (Fig 9A) are shown for DIMS/FRODA (purple), DIMS/DIMS (blue), and FRODA/FRODA (green). The largest value maxk, j(δh(k; Q ∣ P), δh(j; P ∣ Q)) is the actual Hausdorff distance. For illustration purposes, nearest neighbor distances are plotted as a function of frame number k normalized to the interval [0, 1] (i.e., k/∣P∣), where 0 (1) corresponds to the first (last) frame. In general, an appropriate one-dimensional order parameter should be chosen in order to plot nearest neighbor distances for structurally corresponding trajectory frames. (PDF) [file pcbi.1004568.s017.pdf]
