# Supplementary material for: Path Similarity Analysis: A Method for Quantifying Macromolecular Pathways
Source: PLoS Comput Biol. 2015 Oct 21;11(10):e1004568. doi: 10.1371/journal.pcbi.1004568 (PMC4619321; doi:10.1371/journal.pcbi.1004568)
Supplement: S1 Table — The NMP-CORE angle and LID-CORE angle for E. coli EcoAdK or models of EcoAdK based on crystal structures of homologous proteins [99] was computed from the Cα atoms in the NMP, LID and CORE domain as described in Methods. The angles are plotted in Fig 9A. (PDF) [file pcbi.1004568.s018.pdf]

Supporting Information

S1 Tab. **Numerical values for domain angles for experimental crystal structures of AdK.**

References

[1] Beckstein O, Denning EJ, Perilla JR, Woolf TB. Zipping and Unzipping of Adenylate Kinase: Atomistic Insights into the Ensemble of Open ↔ Closed Transitions. J Mol Biol. 2009 Nov;394(1):160–176.

[2] Beckstein O, Denning EJ, Woolf TB. AKeco Structure Library; 2015. Available from: `http://dx.doi.org/10.5281/zenodo.29243`.

Table 1. Domain angles for experimental crystal structures of AdK.

PDB ID	chain	NMP-CORE	LID-CORE
1AK2	A	71.6°	97.9°
1AKE	A	45.3°	106.1°
	B	44.7°	106.7°
1AKY	A	43.8°	112.1°
1ANK	A	45.5°	107.3°
	B	45.2°	106.9°
1DVR	A	67.4°	113.2°
	B	69.8°	108.9°
1E4V	A	45.1°	105.8°
	B	45.0°	105.8°
1E4Y	A	44.6°	111.5°
	B	45.1°	116.0°
1P3J	A	44.1°	109.5°
1S3G	A	45.9°	108.7°
1ZAK	A	45.9°	120.3°
	B	45.9°	114.9°
1ZIN	A	45.7°	122.9°
1ZIO	A	45.1°	124.8°
1ZIP	A	45.3°	123.5°
2AK2	A	69.4°	101.3°
2AK3	A	50.5°	154.4°
	B	53.4°	164.7°
2AKY	A	43.1°	109.0°
2AR7	A	63.2°	119.4°
	B	56.0°	131.0°
2BBW	A	56.0°	128.1°
	B	57.0°	116.7°
2C9Y	A	65.4°	89.2°
2ECK	A	45.2°	106.3°
	B	45.1°	106.8°
2EU8	A	44.3°	113.3°
	B	44.1°	111.6°
2OO7	A	44.2°	112.9°
2ORI	A	44.6°	113.0°
	B	44.3°	112.2°
2OSB	A	44.0°	113.1°
	B	44.1°	112.0°
2P3S	A	44.2°	110.8°
2RGX	A	44.7°	123.2°
2RH5	A	68.2°	141.1°
	B	68.1°	139.7°
	C	68.6°	141.9°
3AKY	A	43.7°	111.2°
4AKE	A	74.7°	145.9°
	B	74.0°	141.7°

The NMP-CORE angle and LID-CORE angle for *E. coli* AdK (AK_{eco}) or models of AK_{eco} based on crystal structures of homologous proteins [1] were computed from the C_α atoms in the NMP, LID and CORE domain as described in Methods. The structures and structural models are available in the *AKeco Structure Library* under a Creative-Commons Attribution ShareAlike License [2].
